# Loss of FBXO11 establishes a stem cell program in acute myeloid leukemia by dysregulating LONP1

**DOI:** 10.1172/JCI181943

**Published:** 2025-11-25

**Authors:** Hayle Kincross, Ya-Chi Angela Mo, Xuan Wang, Linda Chang, Gerben Duns, Franziska Mey, Jihong Jiang, Zurui Zhu, Naomi Isak, Harwood Kwan, Tammy T.Y. Lau, T. Roderick Docking, Pranav Garg, Jessica Tran, Shane Colborne, Se-Wing Grace Cheng, Shujun Huang, Nadia Gharaee, Elijah Willie, Jeremy D.K. Parker, Joshua Bridgers, Davis Wood, Ramon I. Klein Geltink, Gregg B. Morin, Aly Karsan

**Affiliations:** 1 Department of Basic and Translational Research, BC Cancer Research Institute, Vancouver, British Columbia, Canada.; 2 Experimental Medicine Program,; 3 Department of Pathology and Laboratory Medicine,; 4 Centre for Comparative Medicine, and; 5 Department of Medical Genetics, University of British Columbia, Vancouver, British Columbia, Canada.

**Keywords:** Hematology, Stem cells, Hematopoietic stem cells, Leukemias, Mitochondria

## Abstract

Acute myeloid leukemia (AML) is an aggressive cancer with very poor outcomes. To identify additional drivers of leukemogenesis, we analyzed sequencing data from 1,727 unique individual patients with AML, which revealed mutations in ubiquitin ligase family genes in 11.2% of samples from adult patients with AML with mutual exclusivity. The SKP1/CUL1/F-box (SCF) E3 ubiquitin ligase complex gene, *FBXO11*, was the most significantly downregulated gene of the SCF complex in AML. We found that FBXO11 interacts with and catalyzes K63-linked ubiquitination of LONP1 in the cytosol, to promote LONP1 entry into mitochondria. We show that depletion of *FBXO11* or *LONP1* reduced mitochondrial respiration through impaired LONP1 chaperone activity to assemble electron transport chain Complex IV. Reduced mitochondrial respiration secondary to *FBXO11* or *LONP1* depletion imparted myeloid-biased stem cell properties in primary CD34^+^ hematopoietic stem and progenitor cells (HSPCs) in vitro. In a human xenograft model, depletion of *FBXO11* cooperated with *AML1-ETO* and mutant *KRAS*^G12D^ to generate serially transplantable AML. Our findings suggest that reduced FBXO11 cooperates to initiate AML by priming HSPC for myeloid-biased self renewal through attenuation of LONP1-mediated regulation of mitochondrial respiration.

## Abstract 2

<a>Expression of </a><a>*FBXO11* </a><a>is depleted in acute myeloid leukemia, which promotes stem cell quiescence by attenuating mitochondrial metabolism through mislocalization of LONP1.</a>

## Graphical abstract

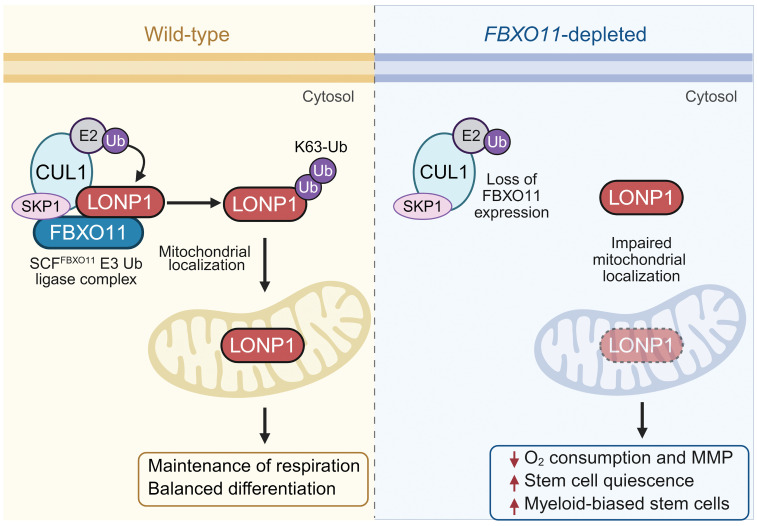

## Introduction

Despite the introduction of new targeted therapies for acute myeloid leukemia (AML), outcomes remain dismal, with 5-year survival being about 30% overall, and around 10% in those over 60 years old (1). Various sequencing studies have generated molecular classification systems for AML (2, 3). Nevertheless, in a large targeted sequencing study comprising 1,540 patients with AML, which established 11 distinct molecular subclasses of AML based on cytogenetic abnormalities and driver mutations, at least 11% of the patients could not be classified into a specific group (3). Analyses of gene expression studies have suggested that additional genetic, epigenetic, and/or clinical parameters play a role in refining genetic classification, and it has been suggested that half of all driver mutations in cancer remain to be discovered (2, 4, 5).

Disruption of the ubiquitin pathway plays an important role in cancer development, as suggested by recurrent genetic aberrations affecting E3 ligases and deubiquitinases in multiple cancers (6–9). Ubiquitination plays a crucial role in the development and function of normal hematopoietic stem and progenitor cells (HSPCs) and leukemic stem cells (LSCs), but ubiquitin gene mutations are thought to be uncommon and do not currently constitute a molecular subclass in AML (10–14).

The presence of a ubiquitin pathway mutation has generally been extrapolated to mean that the ubiquitin-proteasome system is defective (9, 15, 16), but it is clear that different ubiquitination lysine linkages may also result in activation of protein function (17). Results of clinical trials with proteasome inhibitors are conflicting and suggest that ubiquitination functions that are independent of proteasome-mediated degradation may play an important role in AML (18). TRAF6 is a clear example of a ubiquitin E3 ligase with an activating role in myeloid malignancies by mediating lysine (K)63-linked polyubiquitination (19).

A major issue in molecular characterization of AML is that it is difficult to identify subclasses that are comprised of rare mutations in many different genes across a molecular subclass. We found recurrent somatic mutations affecting multiple components of ubiquitinating enzyme complexes in the AML Personalized Medicine Project (AML PMP) cohort (2) and confirmed these findings in the Beat AML, TCGA AML, and TARGET AML datasets, totaling 1,727 unique samples (20–23). By combining mutation analyses and gene expression changes, we identified diminished function of FBXO11 — a member of the SKP1-CUL1-F-box (SCF) E3 ligase family — to be a key aberration that imparts a quiescent, myeloid-biased, self-renewing phenotype. Loss of *FBXO11* was evidenced by truncating mutations or copy number loss, and lower mRNA and protein expression in AML compared with normal HSPCs. We found FBXO11 to interact with the mitochondrial protein LONP1 in the cytosol and, through K63-linked ubiquitination, promote mitochondrial entry of LONP1. Conversely, *FBXO11* depletion, as seen in AML, restricted mitochondrial entry of LONP1, resulting in reduced assembled Complex IV of the electron transport chain and attenuation of mitochondrial respiration. Consistent with previous studies showing that reduced mitochondrial respiration activates a stem cell program (24–27), we found that *FBXO11* depletion induced a myeloid-biased, quiescent stem cell phenotype, and, in cooperation with other mutations, induced myeloid leukemia in a human xenograft model. Our results identify FBXO11 as a regulator of HSPC differentiation, and, when lost, contributes to leukemogenesis.

## Results

### Ubiquitin pathway mutations are frequent and mutually exclusive in AML.

**Figure 1:**
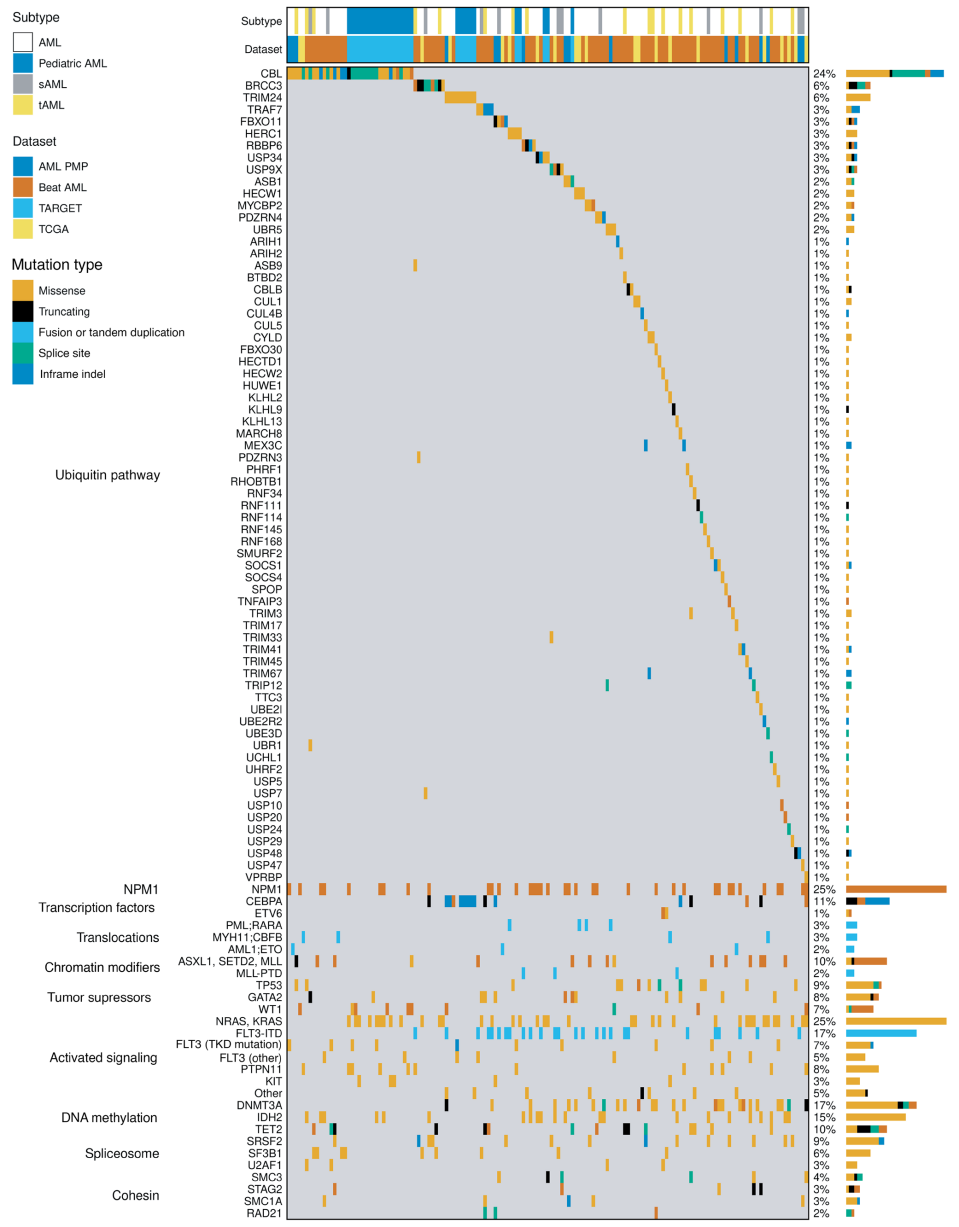
Ubiquitin pathway mutations are frequent and mutually exclusive in AML. Patients with AML with ubiquitin pathway mutations from AML PMP, TCGA, TARGET, and Beat AML data sets are represented in an oncoprint. Shown are 149 of 1,727 total patients with AML with ubiquitin pathway mutations along with cooccurring somatic mutations, representing 11.2% of adult (AML PMP, TCGA, Beat AML) and 4.5% of pediatric (TARGET) AMLs. Columns represent individual AML samples, and rows represent individual genes. Additional variants not shown are recorded in Supplemental Table 1.

We analyzed RNA-seq data from the AML PMP dataset (2) to identify recurrent mutation classes that contribute to the initiation or maintenance of AML. In addition to single nucleotide variants (SNVs) and insertion and/or deletion mutants (indels), as previously described, we identified SNVs and indels in ubiquitin pathway genes in 11.4% (16 of 140) of the AML PMP cohort (Figure 1, Supplemental Figure 1A and Supplemental Table 1; supplemental material available online with this article; https://doi.org/10.1172/JCI181943DS1) (3, 28). Extending the cohort to include AML PMP, TCGA, and Beat AML datasets for a total of 1,062 adult patients with AML revealed ubiquitin pathway gene mutations in 11.2% of cases (119 of 1062) (Figure 1 and Supplemental Table 1). In contrast, only 4.5% (30 of 665) of pediatric patients with AML had ubiquitin pathway mutations (Figure 1 and Supplemental Table 1). Approximately 94% (140 of 149) of AMLs with ubiquitin pathway mutations only had 1 mutation, consistent with mutual exclusivity (Figure 1), as seen with other mutation classes with related biochemical function such as methylation and splicing (3, 21, 29). Loss-of-function mutations, including truncating mutations and copy number loss, for members of the SKP1-CUL1-F-box (SCF) E3 ubiquitin ligase complex were identified in 8.6% (91 of 1062) of adult patients with AML (Figure 1 and Supplemental Figure 1B). Although there is evidence that deregulated ubiquitination plays a role in leukemogenesis (9, 30), these current findings implicate ubiquitin pathway mutations as an independent and distinct molecular class in AML (Figure 1).

### Expression of SCF gene FBXO11 is reduced in AML compared with normal HSPC.

**Figure 2:**
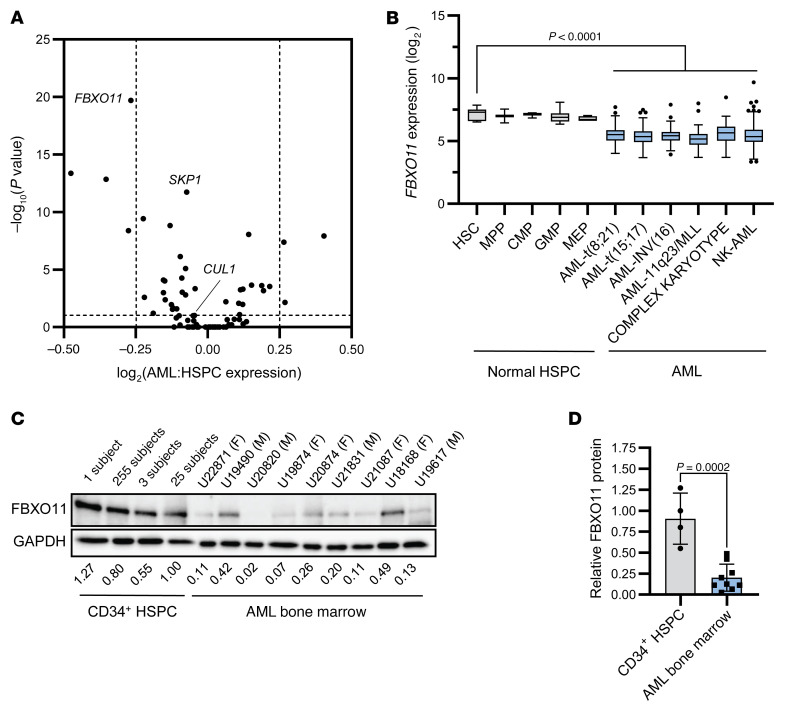
Expression of SCF gene *FBXO11* is reduced in AML compared with normal HSPCs. (**A**) Shown is a volcano plot comparing transcript expression of SCF genes. *P* values represent Bonferroni-adjusted 2-tailed *t* tests. The horizontal dotted line represents *P* = 0.05. Vertical lines represent log_2_ fold change of |0.25|. (**B**) Expression levels of *FBXO11* transcripts across different AML subtypes and normal HSPC populations from BloodSpot are represented as a box and whisker plot. The median is shown with a horizontal line, with the edges of the box corresponding to the first and third quartiles of the distribution. *P* values represent 1-way ANOVA with Dunnett’s test. (**C**) FBXO11 protein abundance in normal CD34^+^ cord blood cell pools comprising 1–255 participants (*N* = 4), and AML bone marrow samples (*N* = 9). Biological sex indicated as female (F) or male (M). Relative FBXO11 protein (fold-change) is indicated normalized to GAPDH expression, and relative to the 25-participant pool. (**D**) Quantification of FBXO11 protein abundance in AML relative to normal CD34^+^ HSPC presented in **C**. *P* value represents a 2-tailed *t* test, error bars represent SD.

To evaluate the potential implications of aberrant ubiquitination in AML secondary to gene expression changes, we compared expression of SCF genes in normal CD34^+^ HSPC and AML cells. Of the 75 SCF genes, *FBXO11* was the most significantly downregulated in AML compared with normal CD34^+^ HSPC (Figure 2A and Supplemental Table 2), which was also among the recurrently mutated ubiquitin pathway genes (Figure 1). Hence, we focused on *FBXO11*, which encodes the substrate-recognition component of the SCF^FBXO11^ E3 ubiquitin ligase complex. *FBXO11* variants occur in 1.8% of all non-AML cancers in the TCGA dataset (Supplemental Figure 2A and Supplemental Table 3). Of the 11 patients with *FBXO11* mutations across datasets, 2 patients had a truncating or frameshift mutation, and 7 patients had a single-copy deletion (Supplemental Figure 2B), suggesting haploinsufficiency of *FBXO11* in AML. Consistent with loss-of-function mutations, AML samples with *FBXO11* mutations or deletions had lower *FBXO11* transcript expression than WT cases (Supplemental Figure 2C).

To determine whether *FBXO11* expression could be regulated in AML independently of genetic variants, we evaluated *FBXO11* expression across AML subtypes. All subtypes had significantly reduced expression compared with normal CD34^+^ HSPC (Figure 2B). Accordingly, FBXO11 protein abundance was lower in AML cell lines compared with the chronic myeloid leukemia–derived K562 cell line (Supplemental Figure 2D). Importantly, bone marrow samples from patients with AML had significantly reduced FBXO11 protein abundance relative to normal cord blood CD34^+^ HSPC (Figure 2, C and D), or adult bone marrow CD34^+^ HSPC (Supplemental Figure 2, E and F). Thus, reduced abundance of FBXO11 is common in AML.

### Depletion of FBXO11 promotes the maintenance of HSPC with myeloid bias.

**Figure 3:**
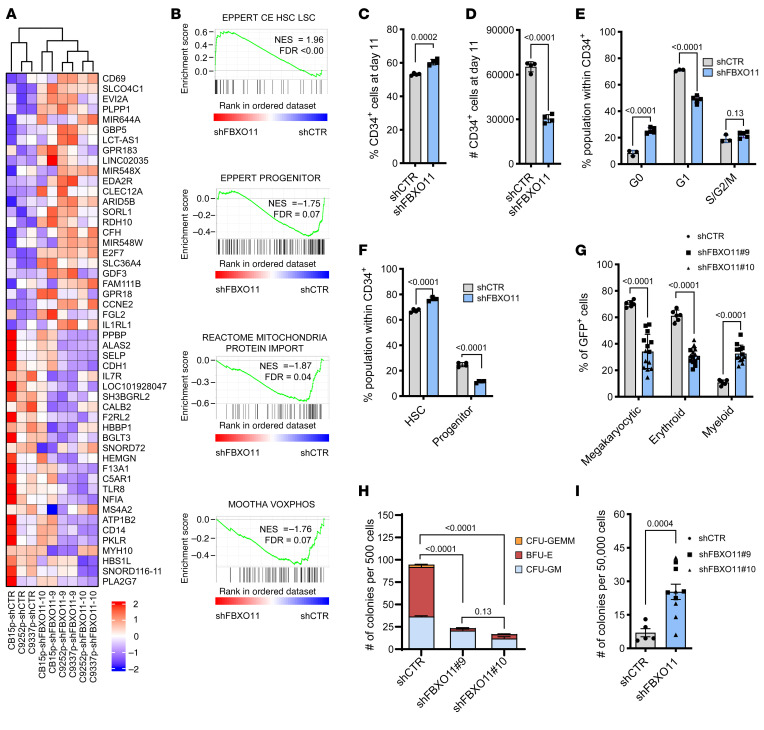
Depletion of *FBXO11* promotes the maintenance of HSPCs with myeloid bias. (**A**) Heatmap arranged according to pairwise clustering based on the 25 most upregulated and 25 most downregulated genes in *FBXO11-*depleted cells compared with control in 3 CD34^+^ HSPC pools from independent participants expressing a nontargeting control shRNA (shCTR) or targeting *FBXO11* (shFBXO11#9 and #10). At the bottom of the heatmap, the independent HSPC pool and experimental condition are labeled. (**B**) GSEA plots in CD34^+^ HSPC comparing *FBXO11* depletion (shFBXO11#9 and #10) to shCTR. Normalized enrichment score (NES) and false discovery rate (FDR) are indicated. All pathways passing FDR < 0.25 are recorded in Supplemental Table 4. (**C**) Shown are percentages and (**D**) absolute cell counts of CD34^+^ HSPCs expressing shCTR or shFBXO11 at day 7 of culture (*N* = 4). (**E**) Cell cycle state of CD34^+^ HSPC was measured by Ki-67/DAPI staining (*N* = 3 (shCTR), *N* = 5 (shFBXO11)). (**F**) Percentages of CD45RA^–^ (corresponding to cultured HSC immunophenotype) and CD45RA^+^90^–^ (corresponding to cultured hematopoietic progenitor immunophenotype) cells within the CD34^+^ population are shown (*N* = 4). (**G**) Shown are percentages of megakaryocytic (CD41^+^ CD61^+^), erythroid (CD71^+^ CD235a^+^), and myeloid (CD15^+^) cells in CD34^+^ HSPCs expressing shCTR or shFBXO11 after 14 days of culture. (**H**) Primary CFC from CD34^+^ HSPC expressing shCTR or shFBXO11 were counted 12 days after plating (*N* = 5).). *P* values represent 2-way ANOVAs with Tukey’s test. (**I**) Colony counts following secondary plating of 50,000 cells from the primary CFC assay shown in panel **H** (*N* = 5). All other *P* values represent 2-tailed *t* test, error bars represent SD.

To determine the effects of *FBXO11* depletion on human hematopoiesis, we analyzed RNA expression of human cord blood-derived CD34^+^ HSPC lentivirally transduced with short-hairpin (sh)RNA targeting *FBXO11* (shFBXO11), validated to deplete *FBXO11* transcript and protein, or a non-targeting shRNA (shCTR) (Supplemental Figure 3, A and B). Depletion of *FBXO11* in human CD34^+^ HSPC resulted in an enrichment of HSC and LSC signatures and diminishment of progenitor cell signatures (Figure 3, A and B, and Supplemental Table 4). Interestingly, several mitochondria-related and erythroid gene sets were also diminished compared with control (Figure 3B and Supplemental Table 4). Liquid culture of CD34^+^ HSPC resulted in increased CD34^+^ cell proportions with *FBXO11* knockdown compared with control (Figure 3C). As absolute cell numbers of CD34^+^ cells were lower with *FBXO11* depletion, despite making up 60.5% ± 2% of the live cell population (Figure 3, C and D), we postulated that *FBXO11* knockdown promoted quiescence as seen in dormant long-term stem cells. Cell cycle analysis confirmed that *FBXO11* depletion increased the G_0_ fraction in CD34^+^ HSPCs (Figure 3E), which was further supported by gene signatures of quiescence (Supplemental Table 4). Interestingly, although increased in the bulk cultured population of shFBXO11 cells, cell death was attenuated in the CD34^+^ fraction (Supplemental Figure 3C), suggesting a protective effect of *FBXO11* depletion in HSPCs, but a cell death-promoting effect in more mature cells.

To verify a role for *FBXO11* depletion in supporting a stem cell phenotype, we examined the immunophenotype of cultured primary CD34^+^ HSPCs exhibiting functional short-term and long-term stem cell (CD45RA^–^) or progenitor cell (CD90^–^ CD45RA^+^) markers (31). *FBXO11* depletion increased the stem cell-like population, at the expense of the progenitor-like population (Figure 3F and Supplemental Figure 3D). In this culture system, all CD34^+^ cellular subpopulations showed an increase in the G_0_ fraction, consistent with *FBXO11* depletion, inducing quiescence in HSPC (Figure 3E and Supplemental Figure 3E).

To examine cell output in *FBXO11*-depleted cells, we cultured CD34^+^ HSPC following knockdown of *FBXO11*. After 14 days in liquid culture *FBXO11*-depleted cells displayed myeloid-biased output with expansion of CD15^+^ myeloid cells, but reduction in erythroid (CD71^+^/CD235a^+^) and megakaryocytic (CD41^+^/CD61^+^) populations compared with control (Figure 3G). *FBXO11* knockdown resulted in reduced primary colony forming cell (CFC) counts, and colonies generated were almost exclusively myeloid (Figure 3H). Secondary replating of the colonies resulted in increased relative clonogenic activity in *FBXO11-*knockdown cells (Figure 3I), consistent with activation of a quiescent HSPC population. In primary CFC assays, there was significant reduction of erythroid colonies (Figure 3H), in keeping with reduced erythroid gene expression signatures and reduced erythroid representation in liquid culture (Figure 3G, Supplemental Table 4), and consistent with a known role for FBXO11 in erythropoiesis (32). Together, these results suggest that *FBXO11* depletion in human CD34^+^ cells results in a quiescent, myeloid-biased HSPC population with stem cell characteristics.

### FBXO11 promotes mitochondrial trafficking of LONP1.

**Figure 4:**
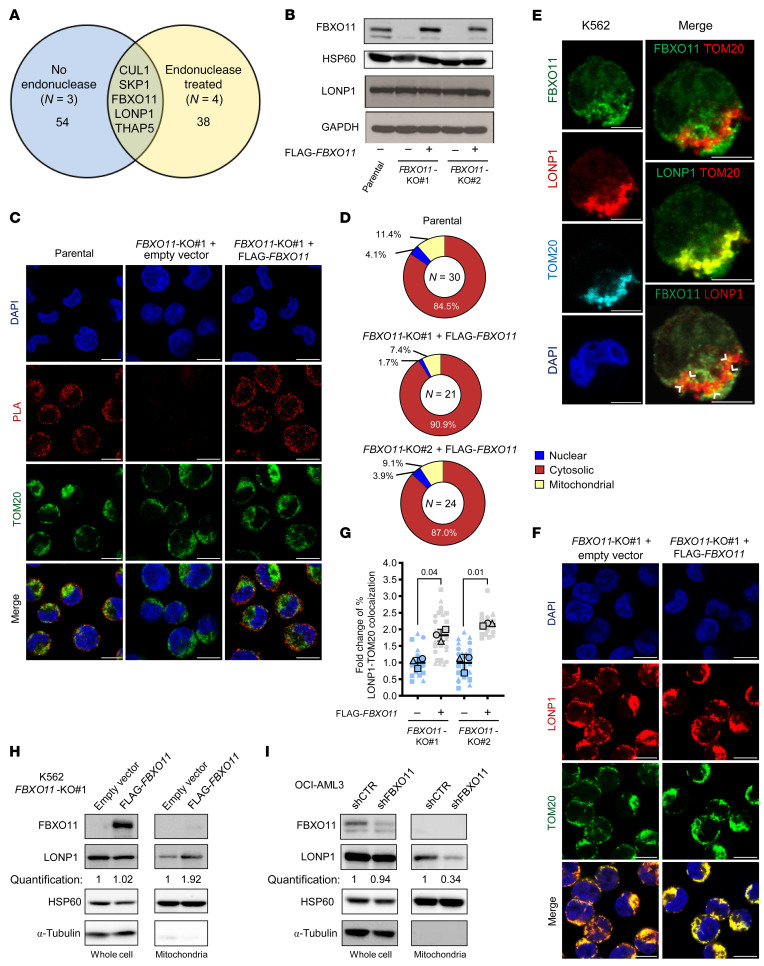
FBXO11 promotes mitochondrial trafficking of LONP1. (**A**) Proteins coimmunoprecipitated with FLAG-FBXO11 in K562 cells with (*N* = 3) or without endonuclease treatment (*N* = 4). (**B**) Whole cell lysates from K562 parental cells or *FBXO11*-KO lines expressing empty vector or FLAG-tagged *FBXO11* (FLAG-*FBXO11*) were immunoblotted as indicated. (**C**) Representative images of proximity ligation assay (PLA) for interaction of FBXO11-LONP1 in K562 parental cells, *FBXO11*-KO#1 transduced with empty vector or FLAG-*FBXO11* cells, coimmunostained for TOM20 and DAPI (scale bars: 10 μm). (**D**) Mean percentage of nuclear, mitochondrial, and cytosolic FBXO11-LONP1 PLA puncta in single cells represented in **C** (and Supplemental Figure 6A) from 3 independent experiments (SD = 2.6%–5.3% (Parental) and 3.1%–4.8% (*FBXO11*-KO + FLAG-*FBXO11*). (**E**) Immunofluorescence staining of indicated endogenous proteins in K562 cells. Pairwise overlays show colocalization in yellow of indicated proteins. FBXO11 + LONP1 colocalization is marked with arrowheads (scale bars: 5μm). (**F**) Immunofluorescent staining for LONP1 localization in *FBXO11*-KO#1 cells transduced with empty vector or FLAG-*FBXO11* and coimmunostained with TOM20 and DAPI (scale bars: 10 μm). (**G**) Superplot of fold-change of LONP1 + TOM20 colocalization for *FBXO11*-KOs + FLAG-*FBXO11* relative to empty vector in single cells presented in **F** (and Supplemental Figure 6B). The means of independent replicates are indicated by unique bordered shapes and data from individual cells with the corresponding borderless shape (*N* = 3). *P* values represent paired 2-tailed *t* test, error bars represent SD. (**H**) Immunoblot for LONP1 expression in whole cell and isolated mitochondrial lysates from *FBXO11*-KO#1 K562 cells transduced with empty vector or FLAG-*FBXO11* (*N* = 2) or (**I**) OCI-AML3 cells expressing shCTR or shFBXO11#9 (*N* = 3). Quantification represents mean LONP1 protein in FLAG-*FBXO11-* or shFBXO11-expressing cells relative to empty vector or shCTR in the indicated lysate.

To identify substrates of the SCF^FBXO11^ complex that could be dysregulated following depletion of *FBXO11* in AML, we immunoprecipitated FLAG-tagged FBXO11 (FLAG-*FBXO11*) in K562 cells and identified coimmunoprecipitating proteins using tandem mass spectrometry. We detected enrichment of 54 proteins, among which were a large proportion of RNA binding proteins (*N* = 3, Figure 4A, Supplemental Figure 4A, and Supplemental Table 5). To ensure these interactions were direct protein-protein interactions and not mediated by RNA intermediates (33), we repeated the experiment with endonuclease treatment which yielded enrichment of 38 proteins (*N* = 4, Figure 4A, Supplemental Figure 4B, and Supplemental Table 5). We identified 5 common coimmunoprecipitating proteins between these 2 independent experiments, including components of the SCF^FBXO11^ complex, SKP1, CUL1, and FBXO11 itself (Figure 4A). Additional proteins included the mitochondrial protease and chaperone LONP1, and the transcriptional repressor THAP5 (Figure 4A). FBXO11 interaction with LONP1 was confirmed by reciprocal coimmunoprecipitation and immunoblotting (Supplemental Figure 5), but we were not able to confirm FBXO11 interaction with THAP5. To our knowledge, a role for FBXO11 in regulating LONP1 or mitochondrial function has not been previously reported. As we observed significant downregulation of mitochondrial pathways in *FBXO11*-depleted CD34^+^ HSPCs (Figure 3B and Supplemental Table 4), our findings suggest that dysregulation of LONP1 may be an uncharacterized consequence of *FBXO11* depletion.

To understand the impact of FBXO11 on LONP1, we generated 2 independent *FBXO11* knockout (KO) clones in K562 cells using CRISPR/Cas9 gene targeting. Surprisingly, LONP1 protein abundance was not affected by *FBXO11*-KO, nor by reconstitution of FLAG-*FBXO11* at levels similar to the endogenous FBXO11 protein, suggesting that LONP1 is not targeted by SCF^FBXO11^ for proteasomal degradation (Figure 4B). To define the subcellular compartment of the FBXO11-LONP1 interaction, we used a proximity ligation assay, which is able to identify proteins localized within 40 nm of each other. Interaction of endogenous FBXO11 and LONP1 occurred primarily in the cytosol, external to the nucleus and the mitochondria, in parental K562 cells (Figure 4, C and D). As expected, knockout of *FBXO11* eliminated FBXO11-LONP1 proximity, and reconstitution of FLAG-*FBXO11* recapitulated these interactions mainly in the cytosol similar to endogenous FBXO11 in parental cells (Figure 4, C and D and Supplemental Figure 6A).

Given that LONP1 is recognized to be a mitochondrial protein, we further explored the subcellular localization of endogenous FBXO11 and LONP1 by confocal microscopy in parental K562 cells. FBXO11 was found to be primarily cytosolic, while the majority of LONP1 localized to the mitochondria (Figure 4E). However, there was colocalization of FBXO11 and LONP1 adjacent to the mitochondria, which was also suggested by the proximity ligation assays (Figure 4, C and D). To investigate whether depletion of *FBXO11* alters localization of LONP1, we examined the topology of these proteins. In *FBXO11*-KO cells LONP1 protein accumulated in the cytosol, while reconstitution of FLAG-*FBXO11* resulted in a greater than 1.8-fold increase in mitochondrial LONP1 localization (Figure 4, F and G, and Supplemental Figure 6B). We confirmed that reconstitution of FLAG-*FBXO11* in *FBXO11-*KO cells did not alter whole-cell LONP1 protein abundance, but specifically increased mitochondrial LONP1 by greater than 1.9-fold, using mitochondrial fractionation and immunoblotting (Figure 4H). Importantly, there was no difference in mitochondrial FBXO11 upon reconstitution of FLAG-*FBXO11* (Figure 4H), suggesting that cytosolic interactions of FBXO11 with LONP1 result in mitochondrial relocalization of LONP1. Using another myeloid cell line, OCI-AML3, we depleted *FBXO11* by shRNA, and correspondingly observed mitochondrial-specific depletion of LONP1 compared with control (Figure 4I). Collectively, these data demonstrate that FBXO11 promotes mitochondrial localization of its interacting protein LONP1.

### The mitochondrial translocation sequence and ATPase domains of LONP1 are essential for interaction with FBXO11.

**Figure 5:**
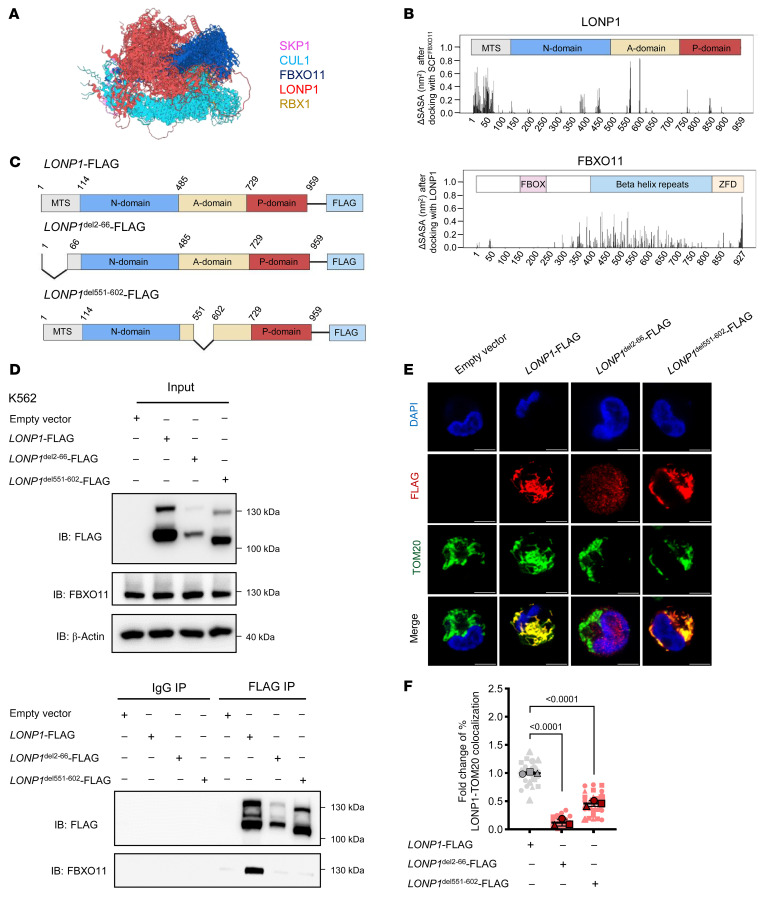
The N-terminus of LONP1 is essential for interaction with FBXO11. (**A**) Representative model of LONP1 docking with the SCF^FBXO11^ complex predicted by AlphaFold3. (**B**) The change in solvent accessible surface area (**Δ**SASA) in nm^2^ averaged across 5 models for each amino acid of LONP1 occluded by FBXO11, and of FBXO11 occluded by LONP1, after docking with SCF^FBXO11^. For LONP1, the mitochondrial translocation sequence (MTS), N-terminal domain (N-domain), ATPase domain (A-domain), and protease domain (P-domain) are indicated. For FBXO11, the F-box domain (FBOX), β helix repeats, and zinc finger domain (ZFD) are indicated. (**C**) Cartoon of domains of WT LONP1-FLAG, and the deletion mutants LONP1^del22–66^-FLAG and LONP1^del551–602^-FLAG. (**D**) Immunoblots of FLAG and FBXO11 following coimmunoprecipitation of the indicated WT or mutant *LONP1*-FLAG constructs from K562 cells. (**E**) Immunofluorescent staining for localization of WT or mutant *LONP1*-FLAG constructs in K562 cells costained with TOM20 and DAPI (scale bars: 10 μm). (**F**) Superplot of fold-change in FLAG-TOM20 colocalization for the indicated WT or mutant *LONP1*-FLAG constructs presented in **E**. The means of independent replicates are indicated by unique bordered shapes and data from individual cells with the corresponding shape (*N* = 3). *P* values represent 1-way ANOVA with Dunnett’s test, error bars represent SD.

To identify the amino acids responsible for FBXO11-LONP1 interaction, we used AlphaFold3 (34) to dock LONP1 with the SCF^FBXO11^ complex (Figure 5A). The 2 regions of LONP1 predicted to be in closest proximity to FBXO11, and thus likely to interact, were the N-terminal region of the mitochondrial translocation sequence (MTS) and part of the ATPase domain (Figure 5B). Using a C-terminal FLAG-tagged LONP1 (*LONP1*-FLAG), we generated deletions to each of these interacting regions ((*LONP1*^del2–66^-FLAG, *LONP1*^del551–602^-FLAG); Figure 5C). Using coimmunoprecipitation followed by immunoblotting, we confirmed that endogenous FBXO11 coimmunoprecipitated with WT LONP1-FLAG as expected, but neither the *LONP1*^del2–66^-FLAG nor *LONP1*^del551–602^-FLAG mutants were able to coimmunoprecipitate FBXO11 (Figure 5D).

As we had previously observed that *FBXO11* depletion resulted in reduction of mitochondrial translocation of LONP1 (Figure 4, F–I, and Supplemental Figure 6B), we evaluated the mitochondrial localization of these LONP1 constructs by immunofluorescence. While WT LONP1-FLAG almost completely localized to mitochondria, approximately 90% of the LONP1^del2–66^-FLAG mutant failed to localize to the mitochondria (Figure 5, E and F), which was unsurprising, as the deleted region of this LONP1 mutant contains part of the MTS (35, 36). The LONP1^del551–602^-FLAG mutant also displayed a reduction in mitochondrial localization (approximately 55%) despite retention of the MTS sequence (Figure 5, E and F), similar to what we observed for endogenous LONP1 in *FBXO11*-KO cells (Figure 4, F and G, and Supplemental Figure 6A). Overall, these data suggest that the N-terminal and ATPase domains of LONP1 are necessary for interaction with FBXO11, which provides further support that FBXO11 and LONP1 interact in the cytosol to promote LONP1 import into the mitochondria.

### FBXO11 depletion impairs LONP1-mediated mitochondrial respiration in AML.

**Figure 6:**
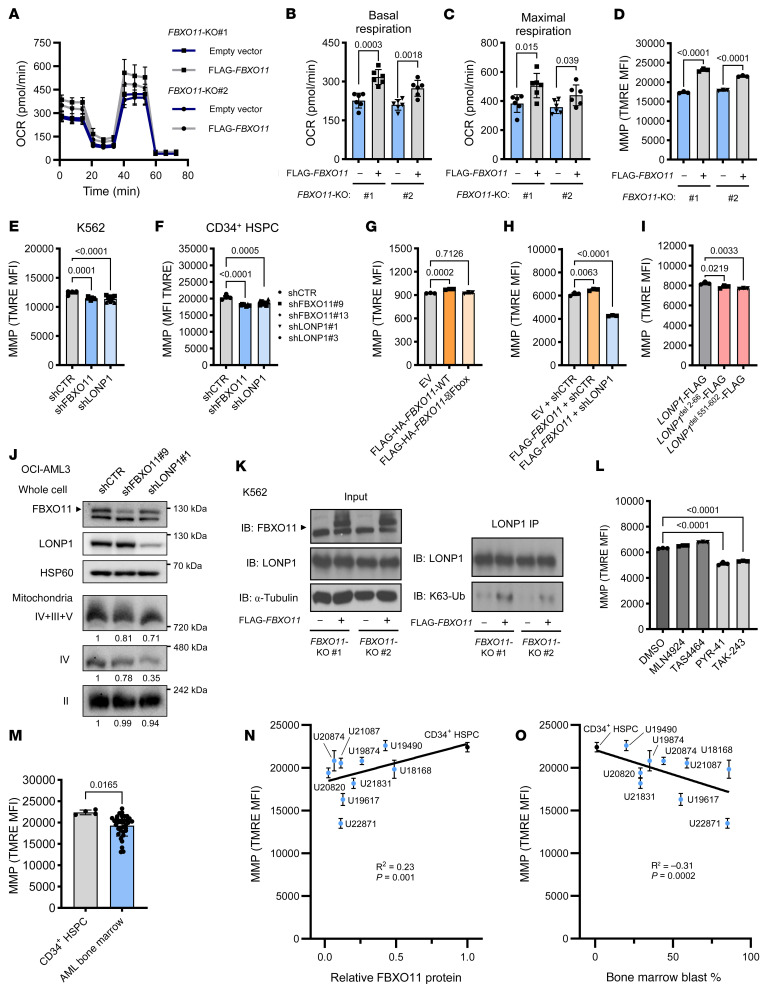
FBXO11 deficiency impairs LONP1 maintenance of mitochondrial respiration in AML. (**A**) Oxygen consumption rate (OCR) measured by the Agilent Seahorse XF Cell Mito Stress Test in K562 *FBXO11*-KO cells expressing empty vector (EV) or FLAG-*FBXO11* with quantification of (**B**) basal and (**C**) maximal respiration (*N* = 6). (**D**) Mitochondrial membrane potential (MMP) of K562 *FBXO11*-KO cells measured by flow cytometry (*N* = 3). *P* values represent 2-tailed *t* tests. MMP of (**E**) K562 cells and (**F**) CD34^+^ HSPCs expressing shRNA targeting *FBXO11* or *LONP1* or a nontargeting control (shCTR) (*N* = 4–5). (**G**) MMP measured in K562 cells expressing WT FBXO11 or FBXO11 lacking the F-box domain (FBXO11-ΔFbox) (*N* = 3). (**H**) MMP measured in K562 cells expressing EV with shCTR, or FLAG-*FBXO11* coexpressed with shCTR or shLONP1 (*N* = 3). (**I**) MMP measured in K562 cells expressing *LONP1*-FLAG or *LONP1*^del22–66^-FLAG and *LONP1*^del551–602^-FLAG (*N* = 3). (**J**) Immunoblots of whole cell FBXO11 and LONP1 levels in OCI-AML3 cells expressing shFBXO11, shLONP1 or shCTR, and assembled ETC complexes (II–V) assayed by blue native page electrophoresis followed by immunoblotting of OCI-AML3 mitochondrial lysates. (**K**) Immunoblots of K63-linked polyubiquitination (K63-Ub) of LONP1 immunoprecipitated from K562 *FBXO11*-KO lines expressing empty vector or FLAG-*FBXO11* (*N* = 3). (**L**) MMP measured in K562 cells expressing FLAG-*FBXO11* treated with vehicle (DMSO), enzyme inhibitors of neddylation (MLN4924 or TAS4464), or ubiquitination (PYR-41 or TAK-243) (*N* = 3). (**M**) MMP of CD34^+^ HSPC from a 25-participant cord blood pool and 9 bone marrow samples from patients with AML presented in Figure 2C (*N* = 3-6). *P* value represents 2-tailed *t* test. (**N**) Correlation of AML sample MMP versus relative FBXO11 protein abundance of the sample or (**O**) reported bone marrow blast percentage at the time of clinical sampling. *P* and R values represent simple linear regression. All other *P* values represent 1-way ANOVA with Dunnett’s test, error bars represent SD.

LONP1 is a mitochondrial protease and chaperone protein that has been reported to mediate assembly and subunit switching for electron transport chain (ETC) complexes (37–39). Consequently, LONP1 loss reduces oxygen consumption rate and mitochondrial membrane potential (MMP) (40, 41). Quiescent long-term HSCs that exhibit long-term engraftment and self-renewal potential have been recurrently demonstrated to be in a low-MMP state (24, 26, 42, 43). Importantly, uncoupling the electron transport chain reduces MMP and promotes quiescence and self renewal of HSC under conditions that normally induce rapid differentiation (25). As we observed downregulated mitochondrial signatures in *FBXO11*-depleted CD34^+^ HSPC (Figure 3B and Supplemental Table 4), we hypothesized that impaired mitochondrial import of LONP1 by *FBXO11* depletion would reduce mitochondrial respiration and MMP, thereby inducing a quiescent stem cell state (Figure 3, B–G). Concordant with this hypothesis, we observed significantly lower basal and maximal respiration rates in *FBXO11*-KO cells compared with *FBXO11*-KO cells reconstituted with FLAG-*FBXO11* (Figure 6, A–C). Indeed, *FBXO11*-KO cells expressing empty vector also showed reduced MMP compared with cells reconstituted with FLAG-*FBXO11* (Figure 6D). As expected, knockdown of *FBXO11* or *LONP1* reduced MMP in 2 myeloid cell lines as well as primary CD34^+^ HSPC (Figure 6, E and F, and Supplemental Figure 3B and Supplemental Figure 7, A and B). We confirmed that the reduction in MMP could not be accounted for by a reduction of mitochondrial mass in *FBXO11-* or *LONP1-*depleted HSPCs or *FBXO11*-KO or FLAG-*FBXO11*-reconstituted cells, as determined by MitoTracker staining and TOM20 abundance (Supplemental Figure 7, C and D).

Long-term HSCs and CD34^+^ HSPC with greater in vivo reconstitution potential display reduced MMP, and disruption of MMP promotes self-renewal in HSCs (27, 44, 25). This is consistent with our data showing that *FBXO11* depletion promotes a quiescent stemness phenotype, which is associated with long-term HSC (Figure 3, B, E, and I, Supplemental Table 4). In contrast, overexpression of WT FLAG-HA-*FBXO11*, but not an F-box domain mutant (FLAG-HA-*FBXO11*-ΔFbox), which fails to assemble the SCF^FBXO11^ complex (45) and disrupts interaction with LONP1 (Supplemental Figure 8), increased MMP, as expected (Figure 6G). To address whether LONP1 is required for the functional effects of FBXO11, we knocked down *LONP1* in cells expressing FLAG-*FBXO11*. *LONP1* knockdown abrogated the increased MMP induced by FLAG-*FBXO11*, indicating that LONP1 is required downstream of FBXO11 to promote mitochondrial respiration (Figure 6H). As expected, K562 cells with enforced expression of either the *LONP1*^del2–66^-FLAG or *LONP1*^del551–602^-FLAG mutants that fail to translocate into the mitochondria had significantly lower MMP than WT *LONP1*-FLAG (Figure 6I). These data suggest that depletion of *FBXO11* with consequent loss of mitochondrial-localized LONP1 (Figure 4, F–I) results in defects in the ETC, which is responsible for maintaining respiration and MMP.

To identify the cause of reduced MMP following depletion of *FBXO11* or *LONP1*, we evaluated the dysregulation of LONP1 activity on the ETC by native blue gel electrophoresis and immunoblotting, which allows for determination of the levels of complete ETC complexes rather than their individual subunits (46). Following knockdown of *FBXO11* or *LONP1* in OCI-AML3 cells, we observed depletion of assembled Complex IV (Figure 6J). We confirmed in our K562 *FBXO11*-KO lines that reconstitution of FLAG-*FBXO11* increased assembled Complex IV (Supplemental Figure 9). We also observed a modest decrease in assembled supercomplex of Complex IV with Complexes III and V with *FBXO11* or *LONP1* depletion in OCI-AML3 cells (Figure 6J). As Complex IV consumes oxygen while pumping protons across the inner mitochondrial membrane to generate MMP, these results are consistent with both the reduced oxygen consumption and MMP observed following *FBXO11*-depletion (Figure 6, A–F, and Supplemental Figure 7B).

As LONP1 protein abundance was not affected by alteration of FBXO11 levels (Figure 4, B, H, and I) and FBXO11 activates mitochondrial LONP1 function (Figure 6, A–H), we assayed for K63-linked ubiquitination of LONP1 by immunoprecipitation of LONP1 under denaturing conditions (47, 48) (Supplemental Figure 10) and immunoblotted for this protein-activating posttranslational modification (Figure 6K). FLAG-*FBXO11* expression increased K63-linked ubiquitination of LONP1 in *FBXO11*-KO lines (Figure 6K). In order to determine whether ubiquitination is responsible for FBXO11-driven mitochondrial function, K562 cells were lentivirally transduced to express FLAG-*FBXO11* to increase basal MMP levels and then treated with ubiquitin E1-activating enzyme inhibitors (Figure 6L). As SCF^FBXO11^ is reported to act as both an E3 ubiquitin ligase as well as an E3 NEDD8 ligase (45), we also treated these cells with NEDD8-activating enzyme inhibitors to determine if interference with either of these posttranslational modifications mimicked the effect of *FBXO11* depletion. We found that treatment with ubiquitination inhibitors PYR-41 or TAK-243 significantly reduced MMP, as was observed with *FBXO11* depletion (Figure 6L). However, treatment with neddylation inhibitors MLN4924 or TAS4464, at concentrations that did not block K63-linked ubiquitination (Supplemental Figure 11A), did not reduce MMP (Figure 6L). As would be predicted, proteasome inhibitors also did not reduce FBXO11-mediated MMP elevation (Supplemental Figure 11B), suggesting that FBXO11-mediated K63-ubiquitination–linked LONP1 activation, rather than proteasomal degradation, is required for regulating mitochondrial function. K63-linked ubiquitination has been shown to play a role in protein cellular localization (49, 50), and, hence, this finding is consistent with data in Figure 4, F–I and showing a requirement for FBXO11 in relocating LONP1 to the mitochondria.

To determine whether mitochondrial respiration was associated with FBXO11 protein abundance in primary samples from patients with AML, we assayed MMP in AML bone marrow samples as well as normal CD34^+^ HSPC. AML samples had significantly reduced MMP compared with CD34^+^ HSPC (Figure 6M), and there was a significant correlation between FBXO11 protein abundance and MMP (Figure 6N), as well as significant negative correlation between leukemic cell burden and MMP in these samples (Figure 6O). These data are consistent with FBXO11 ubiquitinating LONP1 through K63 linkages, allowing mitochondrial localization and maintenance of respiration through properly assembled ETC Complex IV. Conversely, reduced *FBXO11,* as seen in primary AML cells, reduces mitochondrial respiration by attenuating mitochondrial localization of LONP1.

### FBXO11 drives mitochondrial respiration and loss of stem cell phenotype through LONP1.

**Figure 7:**
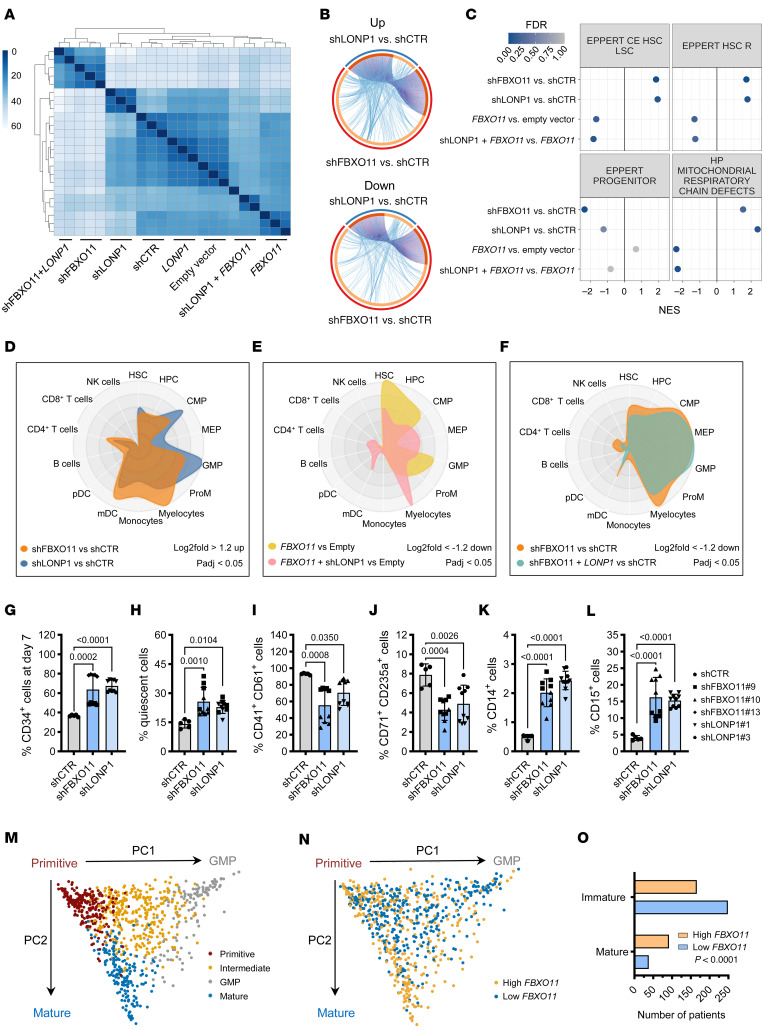
FBXO11 drives mitochondrial respiration and loss of stem cell phenotype through LONP1. (A) Heatmap representing pair-wise Euclidian distances between samples based on expression of all detected genes in RNA-seq data from CD34^+^ HSPC expressing the indicated constructs with each row representing an independent replicate (*N* = 3, except shFBXO11+*LONP1* (*N* = 2)). (**B**) Circos plots comparing genes upregulated (Up) or downregulated (Down) with knockdown of *FBXO11* (red portion of outer ring) or *LONP1* (blue portion of outer ring) in CD34^+^ HSPCs. Dark orange portions of the inner ring represent shared genes between *FBXO11* and *LONP1* knockdown are connected by purple lines, and amber portions represent genes unique to each condition. (**C**) Enrichment trends of indicated gene sets summarized for the comparisons listed. (**D**–**F**) Cell radar plots generated using normal human hematopoiesis (HemaExplorer) data from the BloodSpot database comparing the significantly up- or downregulated genes from the indicated conditions to the hematopoietic populations indicated. (**G**) CD34^+^ HSPC expressing shCTR, shFBXO11 or shLONP1 assessed for CD34 cell surface expression and (**H**) G_0_ phase of the cell cycle by Ki-67 staining on day 7 of culture, and for (**I**) megakaryocyte (CD41^+^CD61^+^), (**J**) erythroid (CD71^+^CD235a^+^), and (**K** and **L**), myeloid (CD14^+^ or CD15^+^) cell surface markers after 14 days of culture (*N* = 4-5). *P* values represent 1-way ANOVA with Dunnett’s test, error bars represent SD. (**M**) Principal component analysis of combined deconvoluted cellular hierarchies for samples from patients with AML from the TCGA, BEAT-AML, and Leucegene cohorts (*N* = 864). (**N**) Samples from patients with AML in **M** stratified by *FBXO11* expression into tertiles within their datasets with the top (High *FBXO11*) and bottom (Low *FBXO11*) tertile projected by principal component analysis. PC, principal component. (**O**) Cellular hierarchy cluster distribution for *FBXO11* high versus low AML samples in **N** (Immature = Primitive + Intermediate + GMP hierarchies). *P* value represents χ^2^ test.

To confirm that FBXO11 regulates the stem cell state through LONP1, we performed RNA-seq on primary CD34^+^ HSPCs expressing shRNAs targeting *FBXO11* or *LONP1* alone, or combined with enforced *LONP1* or FLAG-*FBXO11* expression (Supplemental Figure 12). Samples expressing *LONP1* showed similar transcriptomic states as control shRNA or empty expression vectors (Figure 7A), suggesting that overexpression of *LONP1* is not sufficient to induce functional effects without ubiquitination by FBXO11. Similarly, samples with *FBXO11* knockdown and enforced *LONP1* expression (shFBXO11 + *LONP1*) clustered with *FBXO11* knockdown samples (Figure 7A), again consistent with the idea that LONP1 requires ubiquitination by FBXO11 to enter the mitochondria and function; thus, expressing LONP1 in the absence of FBXO11 does not result in overt differences in transcriptomic state.

Given the finding that LONP1 requires FBXO11 for its function, we compared differentially expressed genes for shFBXO11 and shLONP1. We found that 63.3% of the upregulated genes (309 of 488) and 52.4% of the downregulated genes (253 of 483) in *LONP1*-knockdown CD34^+^ HSPC were shared with *FBXO11* knockdown HSPC (Figure 7B). However, these transcripts represented only 21% (309 of 1,470) of the significantly upregulated and 19.8% (253 of 1275) of downregulated genes seen with *FBXO11* knockdown CD34^+^ HSPCs (Figure 7B). These findings are compatible with the expectation that FBXO11 ubiquitinates multiple proteins, while LONP1 activity is dependent on FBXO11-mediated ubiquitination.

We next performed GSEA to confirm that the stem cell phenotype and mitochondrial defects induced by *FBXO11* depletion are dependent on LONP1 function. Both *FBXO11* and *LONP1* knockdown positively enriched for HSC/LSC gene sets as well as mitochondrial respiratory chain defects (Figure 7C). FLAG-*FBXO11* expression had the opposite effect, with negative enrichment of these gene sets. However, *LONP1* depletion in FLAG-*FBXO11*–expressing CD34^+^ HSPC reversed the transcriptional effect of FLAG-*FBXO11* expression, indicating that FBXO11-directed loss of stemness is mediated by LONP1 activity. An inverse of these transcriptional trends can be seen for the hematopoietic progenitor gene set, as noted previously (Figure 3, B and F, and Supplemental Table 4). These results further support the role of LONP1 as a mediator of FBXO11-driven mitochondrial respiration and stem cell differentiation.

Next, we examined whether *FBXO11* or *LONP1* knockdown enriched for a stem cell transcriptomic state and myeloid bias, as seen in Figure 3. As shown in the cell radar plot, genes upregulated with knockdown of *FBXO11* or *LONP1* shared similar transcriptomic profiles, with enrichment of genes associated with HSC and myeloid cell populations, including common myeloid progenitors (CMP), granulocyte-monocyte progenitors (GMP), myelocytes, and myeloid dendritic cells (mDC) (Figure 7D). Aligning with these trends, genes downregulated with FLAG-*FBXO11* expression were strongly enriched in HSC, CMP, and GMP-associated genes, but *LONP1* knockdown attenuated this effect and resulted in enrichment of the megakaryocyte-erythroid progenitor state, as expected (Figure 7E). As noted, the predicted cell states with *FBXO11* depletion combined with *LONP1* overexpression highly resembled that of *FBXO11* depletion alone, supporting a model of LONP1-driven mitochondrial respiration that is dependent on FBXO11, but not the converse (Figure 7F).

To confirm the transcriptomic trends observed with *FBXO11* and *LONP1* depletion in a functional model, we assayed CD34^+^ HSPC in liquid culture. On day 7 of culture, *FBXO11-* or *LONP1-*depleted primary HSPC showed an increased proportion of CD34^+^ and quiescent cells relative to control (Figure 7, G and H). On day 14, *LONP1* depletion had a similar effect as *FBXO11* depletion on primary HSPC differentiation, causing a reduction in the CD41^+^CD61^+^ megakaryocytic and CD71^+^CD235a^+^ erythroid populations (Figure 7, I and J) and an increase in the CD14^+^ and CD15^+^ myeloid populations (Figure 7, K and L), corroborating our transcriptional findings.

To determine whether *FBXO11* expression was associated with differentiation in samples from patients with AML, we leveraged a framework for characterizing cellular hierarchies in primary AML samples based on bulk RNA-seq (51). Briefly, gene expression was used to deconvolute the cellular hierarchy of the sample using single cell RNA-seq references for leukemic and nonleukemic hematopoietic populations, which separates AML samples into 4 clusters based on their position in the cellular hierarchy (Figure 7M). We applied these methods to the 864 samples originally used (51), from the TCGA, Beat AML, and Leucegene cohorts (21, 20, 52), and stratified the samples into tertiles based on their *FBXO11* expression (Figure 7N). While individual mutations in single genes were unable to cluster samples within these hierarchies (51), we found that the *FBXO11-*low AML samples were significantly enriched in the clusters with high proportions of immature signatures (Primitive + Intermediate + GMP) at the expense of samples in the mature cluster (Figure 7O). Collectively, these findings suggest that *FBXO11* depletion induces an immature myeloid-biased state in both normal CD34^+^ HSPC and in AML cells.

### FBXO11 depletion cooperates with AML1-ETO and KRAS^G12D^ to generate human myeloid leukemia.

To determine whether *FBXO11* deficiency cooperates with driver mutations to initiate AML in vivo, we revisited our analyses of AML samples. One patient with an *FBXO11* mutation had a cooccurring *AML1-ETO* (*RUNX1-RUNX1T1*) fusion (Figure 1), and we found that ubiquitin pathway mutations cooccur with *RAS* mutations (Supplemental Figure 13). Previous studies have shown that the combination of canonical full-length *AML1-ETO* with mutant *RAS* is not sufficient to transform human CD34^+^ cells, likely due to a lack of sufficient self-renewal activity (53). As there is no known combination of mutants with *AML1-ETO* that results in human leukemia (53), we asked whether the combination of *AML1-ETO*, activated *KRAS*, and *FBXO11* depletion could initiate human leukemia.

**Figure 8:**
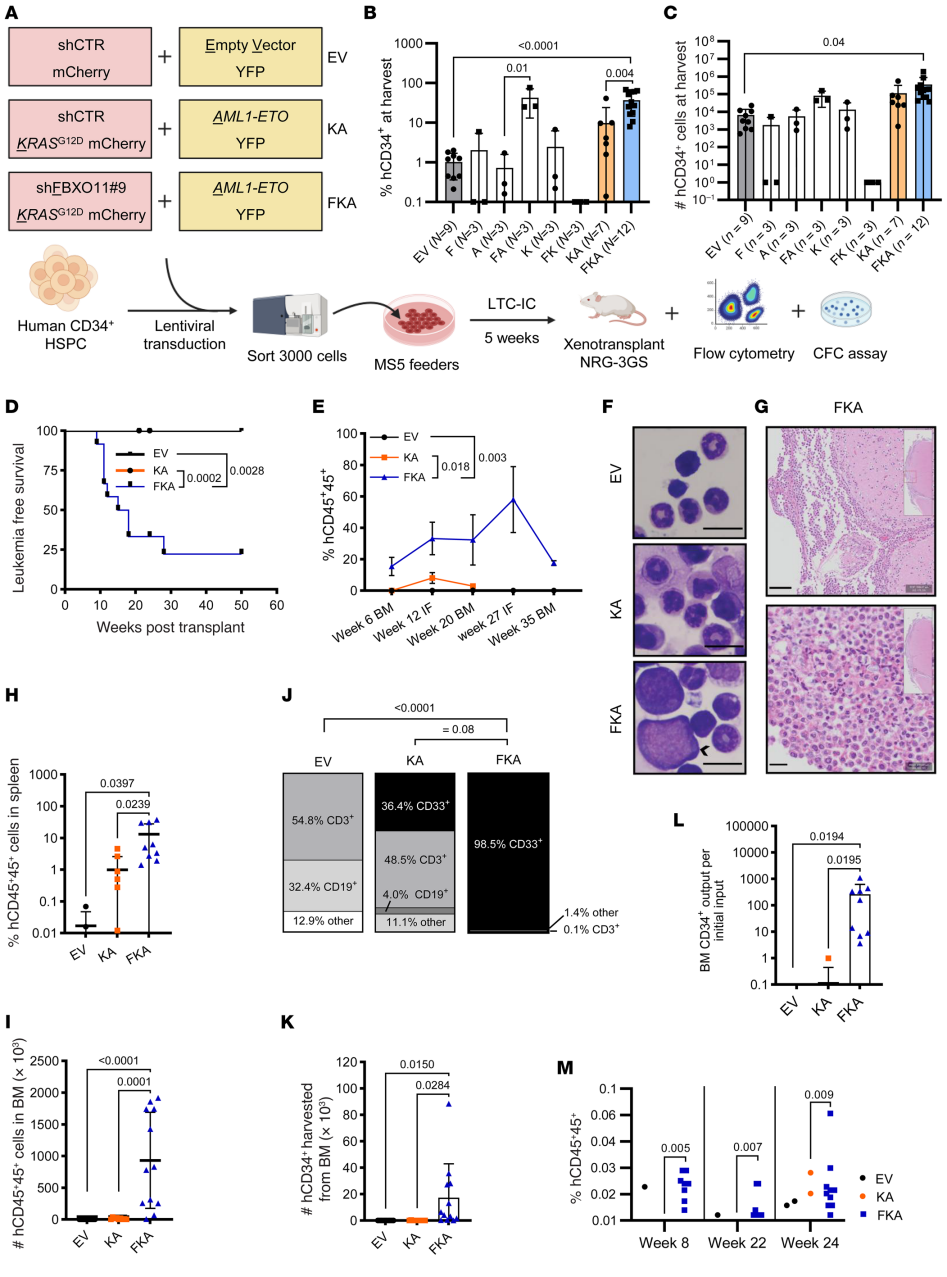
*FBXO11* depletion cooperates with *AML1-ETO* and *KRAS*^G12D^ to generate human myeloid leukemia. (**A**) Schematic of the vector combinations transduced into CD34^+^ HSPC and experimental design of xenotransplantation. (**B**) Percent and (**C**) total live cell counts of CD34^+^ cells measured on day 40 of feeder cell cultures following cotransduction of vectors show in **A**. (**D**) Kaplan-Meier curve for mice transplanted with cells harvested in **B** (*P* = 0.0028 for EV versus FKA, and *P* = 0.0002 for KA versus FKA (Mantel-Cox test)). (**E**) CD45^+^ cell chimerism of transplanted, cotransduced CD34^+^ cells from **B** in the noninjected bone (BM) or injected bone (IF) (*P* = 0.0033 for EV versus FKA, *P* = 0.018 for KA versus FKA [Šidák’s multiple comparisons test]) (for **D** and **E**), EV (*N* = 13); KA (*N* = 9); and FKA (*N* = 12)). (**F**) Representative bone marrow smears at experimental endpoint (scale bar: 10 μm). (**G**) Brain sections of FKA mice with neurologic signs at endpoint showing myeloid infiltrates (scale bars: 100 μm top image; 20 μm bottom image). (**H**) Human CD45^+^ hematopoietic cell engraftment in the spleen at endpoint as percentage of total viable spleen cells (*N* = 5 (EV, 3 not detected (ND)), *N* = 9 (KA, 1 ND), *N* = 12 (FKA)). (**I**) Number of human CD45^+^ hematopoietic cells engrafted in the bone marrow at endpoint. (*N* = 13 (EV, 7 ND), *N* = 9 (KA), *N* = 12 (FKA)). (**J**) Percentage of lymphoid (CD3^+^, CD19^+^), myeloid (CD33^+^), and other (CD3^–^CD19^–^CD33^–^) cells within the viable human CD45^+^ cell population in bone marrow cells at endpoint. (*N* = 5). (**K**) Total CD34^+^ HSPC numbers in the bone marrow quantified at endpoint (*N* = 13 (EV, 13 ND), *N* = 9 (KA, 8 ND), *N* = 12 (FKA)). (**L**) Bone marrow output of CD34^+^ cells per transplanted CD34^+^ cell following LTC-IC (*N* = 13 (EV, 13 ND), *N* = 9 (KA, 8 ND), *N* = 12 (FKA)). (**M**) Hematopoietic cell engraftment in secondary transplants. *P* values represent Fisher’s exact tests. All other *P* values represent 1-way ANOVA with Dunnett’s test, error bars represent SD.

We transduced CD34^+^ HSPC with combinations of these variants: empty vector (EV), *FBXO11* shRNA (F), *AML1-ETO* (A), F+A (FA), *KRAS*^G12D^ (K), F+K (FK), K+A (KA), or all three (FKA) (Figure 8A and Supplemental Figure 14A). We then flow sorted for the coexpressed fluorescent markers and placed the cells into long-term culture on MS5 cells (Figure 8A). Following long-term coculture, *FBXO11* knockdown alone resulted in reduced cell numbers (Supplemental Figure 14B), but significant enrichment of the proportion of CD34^+^ cells when combined with A or KA relative to corresponding controls (Figure 8, B and C, and Supplemental Figure 14, B and C).

Following long-term culture, we xenotransplanted EV, KA, and FKA cells into NOD.*Rag1^–/–^;*γ*c^null^* (NRG) mice expressing human *IL3*, human *GM-CSF*, and human *SCF* (NRG-3GS) (Figure 8A). FKA, but not KA, xenotransplantation of NRG-3GS mice resulted in significantly reduced overall survival (Figure 8D). However, of the mice that survived, those with transplanted FKA cells had significantly longer and higher human hematopoietic reconstitution compared with KA- and EV-transplanted mice, suggesting that *FBXO11* depletion improves long-term in vivo repopulating ability of human HSPCs (Figure 8E and Supplemental Figure 15, A–D).

Morphological examination of FKA mice at endpoint revealed blast cells in the bone marrow and leptomeningeal infiltration with myeloid progenitor cells, consistent with myeloid leukemia (Figure 8, F and G, and Supplemental Figure 16). FKA mice had significantly increased human hematopoietic cell reconstitution in the spleen and bone marrow (Figure 8, H and I), greater than 98% of which were CD33^+^ myeloid cells, which is similar to human AML (Figure 8J and Supplemental Figure 17A). At endpoint, we observed a loss of CD3^+^ T cells at the expense of myeloid expansion only in FKA mice (Figure 8J and Supplemental Figure 17A). FKA-transplanted mice also had greater numbers of CD34^+^ bone marrow cells at endpoint (Figure 8K and Supplemental Figure 17B). Human CD34^+^ cell output posttransplant per initial CD34^+^ cells placed in long-term culture revealed approximately 6,000-fold expansion of FKA HSPCs, consistent with the acquisition of self-renewing properties, while KA expression did not result in CD34^+^ cell expansion (Figure 8L and Supplemental Table 6).

Serial marrow transplantation resulted in enhanced reconstitution of human hematopoietic populations with FKA cells compared with controls, indicating maintenance or expansion of self-renewing hematopoietic cells (Figure 8M). Taken together, these results demonstrate that *FBXO11* depletion improves long-term in vivo human cell reconstitution, consistent with induction of a myeloid-skewed, long-term self-renewing stem cell state that cooperates with *KRAS*^G12D^ and *AML1-ETO* to generate myeloid leukemia.

## Discussion

**Figure 9:**
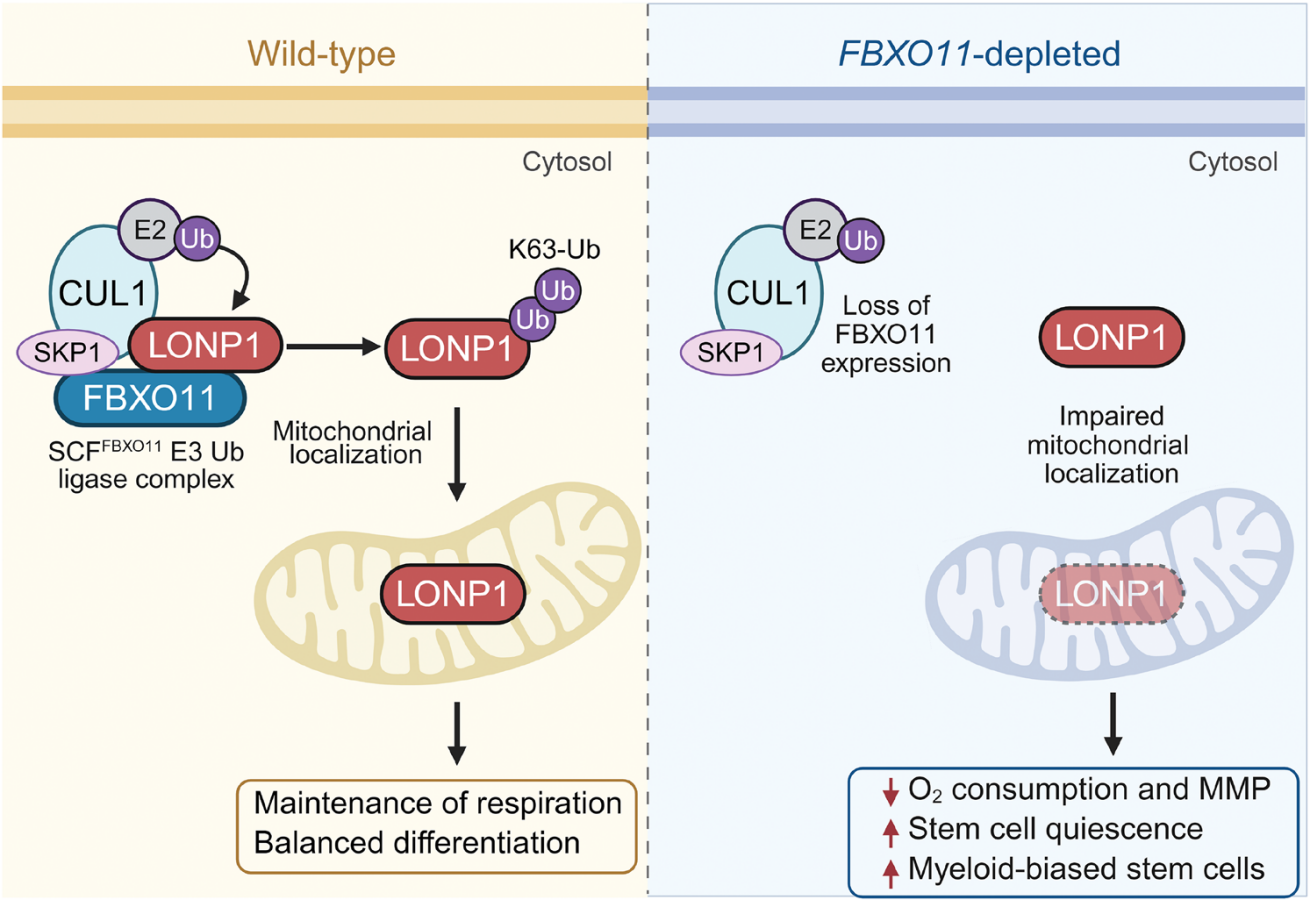
Model for FBXO11 regulation of LONP1 in HSPC. In WT cells, the SCF^FBXO11^ complex catalyzes K63-linked ubiquitination of LONP1, which allows entry of the protein into the mitochondria to maintain respiration and balanced differentiation of hematopoietic cells. However, when FBXO11 is depleted, LONP1 is not K63 ubiquitinated and mitochondrial entry is restricted. This results in decreased oxygen consumption and stem cell quiescence, with myeloid bias.

In this work we highlight ubiquitin pathway mutations as a molecular subclass of AML mutations. We implicate *FBXO11* depletion in leukemogenesis and identify a connection between ubiquitination and mitochondrial function through FBXO11-mediated regulation of the mitochondrial protease and chaperone, LONP1, independent of the proteasome system (Figure 9).

Dysregulation of LONP1 has been associated with colon cancer and melanoma, but has not been studied in the context of AML (40, 54, 55). Our data show that FBXO11 promotes K63-linked ubiquitination of LONP1 to relocate LONP1 into the mitochondria and regulate respiration, and this process becomes dysregulated when *FBXO11* is depleted. Importantly, we demonstrate that LONP1 mutants that cannot bind FBXO11 also display dysregulated mitochondrial localization, supporting the requirement of FBXO11 to relocate LONP1 to the mitochondria. Nevertheless, we cannot completely rule out an alternative mechanism that secondarily affects LONP1 mitochondrial localization. This activating effect of FBXO11 contrasts with other FBXO11 targets previously reported. For instance, SCF^FBXO11^ has been shown to target BCL6, CRL4, and Snail family transcription factors for ubiquitination and subsequent proteasomal degradation (56–58). SCF^FBXO11^-mediated neddylation of p53 also inhibits p53 transcriptional activity (45). Our findings also provide an explanation as to why *LONP1* was not detected in a screen for identifying mitochondrial contributors to leukemogenesis, as the readout in that study was reduced leukemia cell viability with target gene knockdown (59). In our model, it is reduced LONP1 function due to *FBXO11* depletion, not LONP1 activation, that is responsible for leukemogenesis.

Our transcriptomic and functional data identify FBXO11 as an upstream regulator of LONP1 and link *FBXO11* and *LONP1* depletion in regulating mitochondrial function. The mitochondrial respiratory chain defects are consistent with the reduced MMP observed in long-term self-renewing stem cells (24–26, 42, 43). Our findings suggest that reduction of assembled Complex IV of the ETC is responsible for decreased MMP in *FBXO11-*depleted cells. Complex IV depletion has been demonstrated with changes in expression or pathogenic mutations of LONP1 in cells of the lung, heart, liver and muscle, as well as fibroblasts (37–39), but we did not observe differences in Complex II assembly, although this has been previously reported in prostate cancer cells (41). This finding aligns with previous work showing that AML LSC are dependent on Complex II activity (60), and hence we expect that if *FBXO11* depletion is cooperating to initiate AML, Complex II would need to be retained.

While mitochondrial metabolism has recently been a focus in stem cell research, there is no consensus on how exactly reduced mitochondrial function promotes a stem cell state or contributes to leukemogenesis. Metabolic changes and products of metabolic pathways have been shown to impact the epigenome, chromatin remodeling, and nuclear transcription, suggesting one mechanism by which reduced mitochondrial function might promote a stem cell state (61, 62); however, further studies would be required to confirm this.

The role of mitochondria in AML is complex. Our data suggest that reduction, but not total loss of mitochondrial activity, through *FBXO11* or *LONP1* depletion in normal HSPC imparts myeloid-biased stem cell characteristics, with additional activating mutations contributing to AML initiation and maintenance. Indeed, previous studies have shown that AML LSC have reduced oxidative phosphorylation compared with normal HSPCs and are restricted in their use of metabolic fuels (63). LSCs are dependent on amino acids to propel oxidative phosphorylation for their survival, and further blockade of mitochondrial function, such as with the combination of the BCL-2 inhibitor venetoclax and the hypomethylating agent azacytidine, results in cell death (60, 64). Chemotherapy-resistant AML blasts do not utilize amino acids in this manner and instead are uniquely dependent on increased fatty acid oxidation to fuel oxidative phosphorylation (64, 65). Targeting mitochondrial structure and organization has been demonstrated to impair leukemia maintenance and increase sensitivity of resistant AML cells to venetoclax treatment (66). Importantly, there is emerging evidence that Complex IV deficiency, as observed with FBXO11 depletion, may be a wider feature of AML cells than previously recognized (67–70).

Loss of ETC function can result from the accumulation of misfolded ETC proteins shown to occur with *LONP1* loss, or from loss of LONP1 chaperone activity required for proper folding of some ETC proteins (38, 41, 71). We found that both *FBXO11* and *LONP1* depletion reduced assembled Complex IV and MMP, and that FBXO11 acts through K63-linked ubiquitination, regulating relocalization of LONP1 into the mitochondria. Long-term HSCs and CD34^+^ cells that have greater reconstitution potential are both characterized by low MMP (25, 27, 44). Disruption of MMP also promotes self renewal in HSCs, indicating that low MMP is essential for maintaining a stem cell state (25). While *FBXO11* depletion has previously been shown to impart apoptotic effects (72), our work shows that this is not true for HSPC. Rather, *FBXO11*-depleted HSPC are protected from apoptosis and remain in G_0_ of the cell cycle. These findings are consistent with the stem cell characteristics we observed with *FBXO11* depletion, both functionally and at the transcriptional level. Indeed, the stem cell phenotype induced by *FBXO11* depletion is sufficient to cooperate with *AML1-ETO* and *KRAS*^G12D^ to produce a synthetic serially transplantable leukemia from normal CD34^+^ cells.

In this work, we suggest that mutations of ubiquitin pathway genes may represent a molecular subclass in AML. We draw a connection between the ubiquitin pathway and mitochondrial respiration. In particular, we show that LONP1 is a target that is activated, rather than degraded, following FBXO11-mediated ubiquitination, and this acts as a regulator of hematopoiesis. Importantly, we show that depletion of *FBXO11* cooperates with *AML1-ETO* and activated *KRAS*^G12D^ to generate serially transplantable leukemia in a xenograft model. This work suggests that altered gene expression independent of genetic variants may implicate ubiquitin system defects in a much wider cohort of patients with AML**.**

## Methods

### Sex as a biological variable.

All male and female primary AML samples were included in all analyses. Validation of loss of FBXO11 protein in primary samples (Figure 2C) was confirmed in 4 male and 5 female samples, and against one adult female and male samples (Supplemental Figure 2E). One female (K562) and one male (OCI-AML3) cell line were used for validating key phenotypes in vitro. For our in vivo studies, only female mice were used as recipients as they engraft HSPC at higher efficiency than males.

### Mutation calling for oncoprint inclusion.

Patient mutations were determined pathogenic and included in the oncoprint if they had a REVEL score greater than 0.250 and CADD score greater than 10.5. Germline variants were excluded. Hugo Gene Nomenclature Committee (HGNC) gene data was used to generate a list of ubiquitination genes to query, and the list was manually filtered to retain genes functionally relevant to the ubiquitin pathway. For determining the REVEL score threshold of 0.25 for pathogenicity, data for pathogenic solid tumor variants were obtained from *Cheng et al*. (Memorial Sloan Kettering-Integrated Mutation Profiling of Actionable Cancer Targets [MSK-IMPACT]) (73), and variants that were reported less than 5 times in the Catalogue of Somatic Mutations in Cancer (COSMIC), were excluded. Pathogenic variants associated with human hematological cancers were obtained from Jaiswal et al. (74), and variants that were reported less than 5 times in COSMIC were excluded. All mutated ubiquitin pathway genes are ranked by the frequency of their occurrence, and then alphabetically, among genes with an equal number of occurrences (Figure 1).

### Odds ratio calculations.

Odds ratios were calculated as “odds of a mutation occurring in a sample with an ubiquitin pathway mutation” vs “odds of a mutation occurring in a sample without ubiquitin pathway mutations”.

The odds ratio for mutations in gene X was calculated by dividing the number of patients with a mutation in gene X and at least 1 UPS mutation by the number of patients with at least 1 UPS mutation. Then, that result was divided by the result of the number of patients with mutations in gene X without a UPS mutation divided by the number of patients with no UPS mutations.

### Isolation of human HSPCs.

Human HSPC isolation was performed by the Stem Cell assay core at BC Cancer Research Institute. Anonymized normal human cord blood samples were obtained with informed consent from normal full-term cesarean section deliveries in accordance with procedures approved by the Research Ethics Board of the University of British Columbia and samples from a single day were pooled for further processing. CD34^+^ HSPCs were obtained at greater than 95% purity from pooled collections using a 2-step Rosette-Sep/EasySep human CD34^+^ selection kit (STEMCELL Technologies) according to the manufacturer’s protocols and/or FACS sorting. CD34^+^ HSPCs were thawed by dropwise addition into HBSS + 10% FBS supplemented with DNase (100 μg/mL final concentration). CD34^+^ HSPCs were then seeded into 96-well round bottom plates (cell density at 100,000 cells/100 μL) and prestimulated in StemSpan SFEM (STEMCELL Technologies #09650) supplemented with StemSpan CC100 (STEMCELL Technologies #02690), 750 nM SR1 (STEMCELL Technologies #72344), and 35 nM UM171(STEMCELL Technologies #72912) overnight.

### Cell lines.

HEK293T, K562, HL60, and AML-193 were purchased from the ATCC. MOLM-13, Kasumi-1, OCI-AML3, OCI-AML2, and OCI-AML5 were purchased from the Leibniz Institute DSMZ. HEK293T were maintained in DMEM supplemented with 10% FBS. OCI-AML2 was cultured in α Minimum Essential Medium supplemented with 10% FBS. K562, HL60, MOLM-13, OCI-AML3, OCI-AML5, and AML-193 cell lines were cultured in RPMI 1640 medium supplemented with 10% FBS, or 20% FBS for Kasumi-1. Media were supplemented with 2 ng/ml GM-CSF (BioLegend 572902) for OCI-AML5, or 0.005 mg/ml insulin (Sigma-Aldrich 19278), 0.005 mg/ml transferrin (Sigma-Aldrich T1147), and 5 ng/ml GM-CSF for AML-193. All of the cell lines were confirmed by SNP array and STR authentication (Genetica DNA Laboratories) and routinely tested negative for mycoplasma (MycoAlert, Lonza).

### Ex vivo cord blood liquid culture for differentiation assay.

Transduced cells were sorted into 96-well plates at 10,000 cells/well into 100 μL in StemSpan SFEM supplemented with StemSpan CC100, 20 ng/mL recombinant human thrombopoietin (hTPO) (BioLegend #597402), DNase (100 μg/mL final concentration), and antibiotics (penicillin and streptomycin). Cells were incubated in a humidified atmosphere at 37°C in room air supplemented with CO_2_ to achieve a final concentration of 5%.

### Cell differentiation analysis.

CD34^+^ HSPCs were harvested from culture and washed with PBS with 2% FBS before staining with antibodies at the dilutions listed in Supplemental Table 7 for 1 hour on ice. Cells were pelleted at 300*g* for 5 minutes, resuspended in PBS with 2% FBS and 1 μg/mL DAPI, and filtered before flow cytometry analysis.

### Long-term initiating cell (LTC-IC).

Two-to-three days before cell sorting for long-term coculture with MS5 feeder cells, frozen and X-ray–irradiated feeder cells were thawed via dropwise addition into 9 mL DMEM with 15% FBS (15% DMEM) (Invitrogen 12100-046). The cell mixture was spun down at 300*g* for 5 minutes and the supernatant was decanted. The cell pellet was then resuspended in 6 mL 15% DMEM (no antibiotics) and plated at 1 mL/dish in collagen-coated 35 mm dishes (collagen was neutralized with DMEM medium before cell plating). The feeder cells were incubated in a 37° C incubator overnight. The media was replaced with 2 mL fresh 15% DMEM medium the next day. On the day of CD34^+^ HSPCs sorting, the media was removed and replaced with 2.5 mL of LTC media (STEMCELL Technologies #05150) supplemented with 10 μM Hydrocortisone (STEMCELL Technologies #07904).

Weekly half-medium changes were performed by removing one half of the medium containing cells and replacing with freshly prepared H5100 with 10 μM hydrocortisone over the 40-day culture period.

### Xenotransplantation.

NRG-3GS mice (NOD.Cg-*Rag1tm1Mom Il2rgtm1Wjl* Tg(CMV-IL3,CSF2,KITLG)1Eav/J)) were bred and maintained in the Animal Resource Centre of the British Columbia Cancer Research Centre under pathogen-free conditions.

Female NRG-3GS mice 8–15 weeks of age were sublethally irradiated with 850 cGy (cesium irradiation, medium dose rate) on the day of intrafemoral injection. 100,000 cells harvested from LTC were injected per mouse. For transplant analysis of ex vivo cultured cells, bone marrow aspirates were performed every 6 weeks posttransplant and human chimerism was tracked by flow cytometry analysis (using a BD LSR Fortessa analyzer or BD FACSymphony) and analyzed with the following: APC-anti-CD45 2D1 (1:50; Invitrogen 17-9459-42), APC/Cy7 anti-CD45 HI30 (1:50; BioLegend 304014), AF700-anti-CD34 (1:50; BD biosciences 561440), PE/Cy7-anti-CD33 (1:100; Thermo fisher 25-0338-42), PE/Cy7-anti-CD15 (1:100; BD biosciences 560827), BV650-anti-CD19 (1:100; BD biosciences 560827), SB600-anti-CD3(1:50; Invitrogen 83-0037-42), DAPI (1 μg/mL final concentration, Sigma-Aldrich D9542).

Bone marrow aspirates were performed on the noninjected femur (BM, weeks 6, 20, and 35 weeks posttransplant) or the injected femur (IF, weeks 12 and 27 weeks post-transplant) to determine human cell chimerism. Engraftment is considered long term if a detection threshold of greater than 0.01% CD45^+^ cells is exceeded at 20 weeks.

Transplanted mice were sacrificed at predefined, humane clinical morbidity endpoints. At endpoint, human chimerism in bone marrow (cells flushed from femora, pelvises, and tibiae), spleen, and liver were tracked by flow cytometry analysis (using a BD LSR Fortessa analyzer or BD FACSymphony) and analyzed with the following: APC-anti-CD45 2D1 (1:50; Invitrogen 17-9459-42), APC/Cy7-anti-CD45 HI30 (1:50; BioLegend 304014), AF700-anti-CD34 (1:50; BD biosciences 561440), PE-anti-CD33 (1:50; eBioscience 8012-0337-120), PE/Cy7-anti-CD15 (1:100; BD biosciences 560827), BV480-anti-CD14 (1:50; BD biosciences 566190), and DAPI (1 μg/mL final concentration, Sigma-Aldrich D9542).

For secondary transplants, viably frozen bone marrow harvested at primary transplant endpoints were thawed and added dropwise into 8 mL thawing medium (PBS with 10% FBS and 100 μg/mL DNase final concentration). Cells were spun down at 400*g* for 5 minutes to remove the supernatant. Cells were counted, and the total amount of harvested bone marrow for each EV and KA mice were transplanted into each sublethally irradiated recipient NRG-3GS by tail vein injection. Half of the harvested bone marrow from each donor FKA mouse was transplanted into each FKA recipient mouse. Secondary transplanted mice were monitored, processed, and analyzed following the procedures for primary transplants.

### Immunoprecipitation.

For coimmunoprecipitations for identifying protein-protein interactions, cells were washed in PBS then lysed with FLAG-modified RIPA buffer (50 mM Tris-HCl pH 7.6, 150 mM NaCl, 1 mM EDTA, 1% Triton X-100, protease inhibitor cocktail 1 tablet/1 mL at 1:100 [Sigma-Aldrich 11697498001], phosphatase inhibitor cocktail 1:100 [Sigma-Aldrich P0044-5ML], 1 mM Na3VO4 and 10 mM NaF) for 1 hour at 4°C on a rotating platform, then passed 10 times through a 22-G needle. The lysate was centrifuged at 16,000*g* for 10 minutes at 4°C. The supernatant was collected and protein quantification was performed using the BCA assay (BioRad DC Protein assay kit, Bio-Rad 5000112). 1 mg of protein per condition was used for coimmunoprecipitation and the volume was made up to 1 mL with the lysis buffer. The lysate was precleared with Protein G agarose beads (EMD Millipore 16-266) for FLAG immunoprecipitation or Protein A agarose beads (EMD Millipore 16-125) for LONP1 immunoprecipitation on a rotating platform at 4°C, overnight (16 hours) for FLAG immunoprecipitation or for 3 hours for LONP1 immunoprecipitation. Beads were spun down at 1500*g* for 1 minute, and the lysate was transferred to a fresh tube with 50 μL equilibrated ANTI-FLAG M2 Affinity Gel (Sigma-Aldrich A2220-1ML) for FLAG immunoprecipitation, or with Protein A agarose beads incubated with 2 μg anti-LONP1 antibody (ThermoFisher Scientific 15440-1-AP) overnight (16 hours) and incubated on a rotating platform at 4°C for 1 hour for FLAG immunoprecipitation, and for 3 hours for LONP1 immunoprecipitation. The beads were washed with wash buffer (50 mM Tris-HCl pH 7.6, 150 mM NaCl, 0.05% Triton X-100) 5 times with 1 min 1500*g* spins in between, and eluted in 1× Laemmli sample buffer at 95°C for 15 minutes.

For LONP1 immunoprecipitation to identify posttranslational modifications, cells were lysed with a stringent denaturing RIPA buffer (47, 48) (25 mM Tris, 150 mM NaCl, 1% NP40, 0.5% sodium deoxycholate, 0.1% sodium dodecyl sulfate (SDS)) instead of FLAG-modified RIPA buffer.

### Primary AML samples and CD34^+^ HSPC media for measuring mitochondrial membrane potential.

Primary AML samples and CD34^+^ HSPC were cultured in medium previously described (75). STEMdiff APEL2 (STEMCELL Technologies 05275), 2% FBS (Sigma-Aldrich F1051), 2% Chemically Defined Lipids (Thermo Scientific 11905031) supplemented with 25 ng/mL VEGFA (STEMCELL Technologies 78159.1), 25 ng/mL VEGFC (STEMCELL Technologies 78202.1), 25 ng/mL bFGF (STEMCELL Technologies 78134.1), 25 ng/mL hSCF (STEMCELL Technologies 78062), 25 ng/mL Flt3 (STEMCELL Technologies 78009) and 10 ng/mL TPO (STEMCELL Technologies 78210.1), 10 ng/mL EPO (STEMCELL Technologies 78007), 10 ng/mL IL3 (STEMCELL Technologies 78040.1), 10 ng/mL IL6 (Cedarlane Labs 206-IL-200/CF). 50% media change was performed on day 3, 24 hours before TMRE staining.

### Statistics.

For comparisons between two groups, unpaired 2-tailed Student’s t-test was used, with Bonferroni correction for multiple comparisons where appropriate. A paired 2-tailed Student’s *t*-test was used to evaluate paired biological replicates (Figure 4G). 1-way ANOVA with a Dunnett test for multiple comparisons was used for comparison of multiple groups to a single control or a Šidák multiple comparisons test for multiple group comparisons. 2-way ANOVA with a Tukey test for multiple comparisons was used to compare colony type and frequency in primary CFC assays (Figure 3H). Relationships between variables were determined by linear regression. Relationships between two categorical variables were determined by χ2 or Fisher’s exact test. Survival in xenotransplants was evaluated by Kaplan-Meier with the Mantel-Cox test used to determine significance. Experiments were performed in at least 3 biological replicates unless otherwise indicated. All statistical tests were performed using GraphPad Prism version 8.0 (San Diego, California). All data are presented as mean ± SD. Statistical significance was set at a threshold of *P* < 0.05 and FDR < 0.25.

### Study approval.

All use of human primary CD34^+^ HSPC were approved by the Research Ethics Board of the University of British Columbia. All xenotransplantation experimental procedures were approved by the University of British Columbia Animal Care Committee.

### Data availability.

Microarray and RNA-seq data were deposited into GEO (GSE183844). All data values are available in supplemental materials or the Supporting data values document. Additional data supporting the findings of this study are available from the corresponding author on request.

## Author contributions

H Kincross, YCAM, XW, LYTC, GD, FM, JJ, ZZ, NI, JT, NG, JDKP, and RKG contributed to the performance of the experiments and/or analysis of the data. H Kwan, TTYL, TRD, PG, SH, EW, and JB curated data and performed bioinformatics assays. SC and SWGC performed mass spectrometry analyses. DW performed and interpreted pathology on murine specimens. RKG, GBM, and AK provided advice and interpreted the data. AK conceived the study, secured funding, and supervised the project. HK, YCAM, and AK wrote the manuscript. H Kincross and YCAM contributed equally to the study. H Kincross completed the final manuscript.

## Funding support

- Canadian Institutes of Health Research (PJT-162131 and PJT-183924) (to AK).
- The Terry Fox Research Institute (Project #1074) (to AK).
- Tier 1 Canada Research Chair in Myeloid Cancers (to AK).
- Killam Doctoral New Scholar Award (to H Kincross).

## Supplementary Material

Supplemental data

Unedited blot and gel images

Supplemental tables 1-7

Supporting data values

## Version 1 11/25/2025

In-Press Preview

## Version 2 01/16/2026

Electronic publication
