# Supplementary material for: Loss of FBXO11 establishes a stem cell program in acute myeloid leukemia by dysregulating LONP1
Source: J Clin Invest. 2025 Nov 25;136(2):e181943. doi: 10.1172/JCI181943 (PMC12807481; doi:10.1172/JCI181943)
Supplement: Supplemental data [file jci-136-181943-s193.pdf]

Supplemental Information

Loss of FBXO11 function establishes a stem cell program in acute myeloid leukemia through dysregulation of LONP1

Hayle Kincross[1,2,*], Ya-Chi Angela Mo[1,3,*], Xuan Wang[1], Linda Chang[1], Gerben Duns[1], Franziska Mey[1], Jihong Jiang[1], Zurui Zhu[1], Naomi Isak[1], Harwood Kwan[1], Tammy T.Y. Lau[1], T. Roderick Docking[1,2], Pranav Garg[1], Jessica Tran[1], Shane Colborne[1], Se-Wing Grace Cheng[1], Shujun Huang[1], Nadia Gharaee[1], Elijah Willie[1], Jeremy D.K. Parker[1], Joshua Bridgers[1], Davis Wood[4], Ramon Klein Geltink[3], Gregg B. Morin[1,5], Aly Karsan[1,2,3,**]

[1] Department of Basic and Translational Research, BC Cancer Research Institute, Vancouver, British Columbia, Canada.
[2] Experimental Medicine Program, University of British Columbia, Vancouver, British Columbia, Canada.
[3] Department of Pathology and Laboratory Medicine, University of British Columbia, Vancouver, British Columbia, Canada.
[4]Centre for Comparative Medicine, University of British Columbia, Vancouver, British Columbia, Canada.
[5]Department of Medical Genetics, University of British Columbia, Vancouver, British Columbia, Canada.
[*]These authors contributed equally.

[**]Corresponding Author: Aly Karsan, BC Cancer Research Institute
675 West 10th Avenue, Vancouver, British Columbia V5Z 1L3, Canada
Phone: 604.675.8033; e-mail: akarsan@bcgsc.ca

Declaration of interests

The authors declare no conflict of interest exists.

SUPPLEMENTAL METHODS AND MATERIALS

BloodSpot data analysis

Transcriptomic data for SCF genes from the BloodSpot database (https://servers.binf.ku.dk/bloodspot/) were taken from GSE42519 and GSE13159 datasets. Data for *FBXO11* expression taken from the same datasets, with data for the 219208_at probe.

Immunoblotting

Cells were washed with PBS and lysed for 30 min on a rotating platform at 4°C in RIPA buffer (25 mM Tris, 150 mM NaCl, 1% NP40, 0.5% sodium deoxycholate, 0.1% sodium dodecyl sulfate (SDS), protease inhibitor cocktail, phosphatase inhibitor cocktail, 1 mM Na3VO4 and 10 mM NaF). Lysed cells were centrifuged for 16,000 x g for 10 min at 4°C, and the supernatant was collected for protein quantification by BCA. Samples were mixed with Laemmli sample buffer incubated at 95°C on a heat block for 15 min.

Proteins were separated by SDS-polyacrylamide gel electrophoresis at 120 V at room temperature and transferred to nitrocellulose membrane at 100 V at room temperature for 1 hour for proteins of interest under 55 kDa, and at 35 V at 4°C for 16 hours for proteins of interest over 55 kDa. After transfer, blots were rinsed briefly with tris-buffered saline with 0.1% Tween 20 (TBST), and then blocked for 1 hour at room temperature in 5% skim milk in TBST. Membranes were washed 3 times for 10 minutes each with TBST.

Blots were probed with primary antibodies (FBXO11 antibody at 1:1000 (Bethyl Laboratories A301-178A), GAPDH antibody at 1:10,000 (Sigma-Aldrich G8795-200UL), HSP60 1:1000 (Cell Signaling Technology 4870S), LONP1 antibody at 1:1000 (ThermoFisher Scientific 15440-1-AP), K63 linkage specific antibody 1:1000 (Cell Signaling Technology 5621S), NEDD8 antibody at 1:1000 (Cell Signaling Technology 2745S), α-tubulin at 1:1000 (Sigma-Aldrich T5168-.2ML) TOM20 at 1:1000 (New England Biolabs 42406S), FLAG at 1:1000 (Invitrogen, MA1-91878), Total OXPHOS 1:1000 (Abcam, ab110411), Vinculin 1:1000 (Cell Signaling Technology 13901S), β-Actin 1:10,000 (Sigma-Aldrich A1978) were prepared in TBST with 3% bovine serum albumin (BSA), based on manufacturer recommended incubation times. Membranes were washed 3 times for 10 minutes each with TBST and probed with appropriate HRP secondary antibody (anti-Rabbit Immunoglobulin/HRP, Dako P044801 or anti-mouse Immunoglobulin/HRP, Dako P0260) at 1:1000 in TBST with 5% skim milk. Blots were developed with Western Lightning Plus, Chemiluminescent Substrate (PerkinElmer NEL105) and imaged with a BioRad ChemiDoc MP Imaging system.

shRNA constructs and CRISPR clones

K562 control and *FBXO11* knockout CRISPR clones were generated by Dr. Gerben Duns. Guide RNAs (gRNAs) were cloned into the pSpCas9n(BB)-2A-GFP (PX461) plasmid (Addgene #48140) and delivered to parental K562 cells by Amaxa nucleofection using the Amaxa Cell line nucleofector kit V (Lonza VCA-1003). GFP^+ transfected cells were single-cell sorted into 96-well plates, expanded. *FBXO11* targeting was confirmed by Sanger sequencing, and FBXO11 depletion was confirmed by immunoblot. shRNAs used are in the pLKO.1 vector, from the MISSION library, and received from the UBC Centre for High-Throughput Biology, and GFP or mCherry was inserted in place of the puromycin resistance marker. shFBXO11#13 was designed in house and cloned into the pLKO backbone from annealed oligos using the AgeI and EcoRI restriction sites.

***FBXO11* CRISPR gRNAs**

gRNA sequence: AACCACTGTAGGGTTAGCATAGG

Target exon: Exon 12/23 (antisense, positive strand)

Human shRNAs

shCTR

Target sequence: CCTAAGGTTAAGTCGCCCTCGC

shFBXO11 #9

TRC Clone ID: TRCN0000004303

Target exon: Exon16/23 (isoform 4 NM_001190274.2)

Target sequence: GAGTGCTAGAAGACAATGATA

shFBXO11 #10

TRC Clone ID: TRCN0000004304

Target exon: Exon 5/23 (of isoform 4 NM_001190274.2)

Target sequence: GAGAGTTTCCAGCAGTTGTAT

shFBXO11 #13

Target exon: 3’ UTR region (of isoform 4 NM_001190274.2)

Target sequence: AAGGTCAGTTGACACTATGAA

Oligo sequence:

F: CCGGAAGGTCAGTTGACACTATGAACTCGAGTTCATAGTGTCAACTGACCTTTTTTC

R: AATTGAAAAAAAGGTCAGTTGACACTATGAACTCGAGTTCATAGTGTCAACTGACCTT

shLONP1 #1

TRCN0000046793, Exon 11/18 (isoform 1 NM_004793.4)

Target sequence: CCAGTGTTTGAAGAAGACCAA

shLONP1 #3

TRCN0000046797 Exon 2/18 (isoform 1 NM_004793.4)

Target sequence: CCAGCCTTATGTCGGCGTCTT

Lentiviral constructs and transduction

High-titer lentiviral supernatants were produced by transient transfection of 293T/17 cells with second-generation packaging/envelope vectors pRRE (Addgene #12251), pREV (Addgene #115989), and pMD2.g (Addgene #12259) using CalPhos^{TM} mammalian transfection kit (Clontech #631312) and incubated for 16 hours, followed by ultracentrifuge concentration (25,000 rpm for 90 min at 4°C; Beckman SW32Ti rotor).

CD34^{+} HSPCs were transduced in 96-well plates (Thermo Scientific #12-565-65) with concentrated lentivirus. After 6 hours of incubation, cells were washed once with PBS and transferred to culture medium (StemSpan^{TM} SFEM with StemSpan^{TM} CC100 to culture medium at a 1 in 100 dilution, 750 nM SR1 and 35nM UM171). Following 72-hour ex vivo culture, transduced cells were then isolated by sorting for reporter fluorophore expression from lentiviral constructs on a Fusion sorter (Becton Dickenson) using purity mode.

RNAseq extraction and RNAseq

CD34^{+} HSPCs were cultured for 24 hours in StemSpan^{TM} SFEM supplemented with StemSpan™ CC100, 20 ng/mL recombinant hTPO, 750 nM SR1, 35 nM UM171, DNase (100 ug/mL final concentration), and antibiotics after sorting for lentivirally transduced cells as described above before pelleting at 300 x g for 5 min. Cell pellets were resuspended in TRIzol^{TM} reagent (ThermoFisher #15596026). Total RNA was extracted according to manufacturer guidelines. For RNA precipitation, 5 µg glycogen (Roche #10901393001) was added per sample. 40 ng of RNQA per sample was submitted based on quantification by NanoDrop^{TM}. Downstream processing, quality control, and RNAseq were performed at Michael Smith laboratories facilities by 1 x Illumina seq with 150bp PET Indexed Lanes. cDNA was labelled using the Biotin

Allonamide Triphosphate protocol. Target hybridization was performed at 45°C, for 16-18hr using the FS450_0001 fluidics protocol for washing. Data was acquired using the Affymetrix GeneChip Scanner 3000. CEL files were imported into R, and the probe expression values were normalized using the Robust Multichip Average (RMA) method from the 'oligo' (v.1.50.0) package. Probes that were lowly expressed, defined as not having normalized expression values greater than 4 in at least 3 samples, were filtered out. Probes that were ambiguously mapped to multiple genes were filtered out.

Ki-67 staining

CD34$^+$ HSPC were fixed and permeabilized with the BD Fixation and Permeabilization kit (#554714) according to manufacturer protocol. Fixed and permeabilized cells were stained with PE anti-human Ki-67 antibody (BioLegend #350504) diluted 1:10 in the BD Perm/Wash buffer from the kit for 30 min on ice. Unbound antibody was washed with the Perm/Wash buffer. Pelleted cells were resuspended in BD Perm/Wash buffer with 2 µg/ml DAPI and incubated for 10 min in the dark, on ice. Cells were centrifuged at 1500 x g for 5 min and resuspended in PBS with 2% FBS and strained for flow cytometry analysis.

Colony-forming cell (CFC) assay

For primary CFCs, 500 of the sorted lentivirally transduced CD34$^+$ HSPCs were seeded into 1 mL of MethoCult™ H4434 Classic media (STEMCELL Technologies #04434) supplemented with StemSpan™ CC100 with antibiotics, and plated in 35mm CFC dishes (STEMCELL Technologies #27150). Cells were incubated for 10 days before colonies were counted.

For secondary CFCs, the media containing the cells were collected from the CFC dishes and transferred into 15mL tubes. PBS with 2% FBS was used to rinse the plates and pooled with the collected media. Cells were spun down at 300 x g for 5min, washed with PBS with 2% FBS. After cell counting, the cells were seeded into fresh CFC dishes in fresh MethoCult™ H4434 Classic media supplemented with StemSpan™ CC100 and antibiotics at 50k cells/mL in each plate. Colonies were counted after 10 days.

Quantitative tandem mass spectrometry

K562 cells lentivirally expressing FLAG-FBXO11 were lysed in modified RIPA buffer with 10 µM MG132 (ApexBio A2585-25) as described above. Whole cell lysate was incubated with Anti-FLAG® M2 Magnetic Beads (Sigma-Aldrich M8823-1ML) or IgG beads for 3 hours then washed with lysis buffer 3 times. Three replicates per condition were directly eluted with SP3 elution buffer (200 mM HEPES pH 8.0, 10% SDS, 200 mM DTT). Four replicates each were treated with Benzonase® (EMD Millipore 70664-3)

before elution. TMT labelling was performed by the mass spectrometry core at the Genome Sciences Centre. 8-plex TMT labels were used. Quantification results were calculated based on published methods (1, 2).

For the set of immunoprecipitations performed without Benzonase®, proteins had normalized protein enrichment score (PESn) greater than 0.6 and were enriched in 2 of the 3 replicates by at least 1.1-fold were shortlisted. For the immunoprecipitations performed with Benzonase® treatment, proteins that had PESn greater than 0.45 and were enriched in 3 of 4 replicates by at least 1.2-fold were shortlisted. The shortlisted proteins from each experiment were intersected to identify commonly immunoprecipitated proteins.

Immunofluorescence staining, proximity ligation assay, and confocal microscopy

Coverslips (Fisher Scientific 12-545-81) were coated with 0.01% poly-L-lysine solution (Sigma-Aldrich P8920-100ml) for 2 hours, washed twice with ddH_2O, and dried overnight. Cells were incubated in fresh medium for 1 hour on the poly-L-lysine coated coverslips before being washed with PBS, fixed with 3% paraformaldehyde, and permeabilized with 0.01% Triton-X. Samples were blocked with PBS supplemented with 1% BSA, 10 mM $MgCl_2$, and 1 mM $CaCl_2$ for 1 hour at room temperature. Samples were stained with primary antibodies to FBXO11 (1:50, Abnova H00080204-M01) and LONP1 (1:100, Thermo Fisher 15440-1-AP) or FLAG at 1:200 (Invitrogen, MA1-91878), for 2 hours, and secondary antibodies (1:500 goat anti-mouse Alexa Fluor 488, Fisher Scientific A11008 / goat anti-rabbit Alexa Fluor 546, Fisher Scientific A11035) for 1 hour at room temperature.

The Duolink Proximity Ligation Assay (PLA) was performed according to the manufacturer's protocol. Instead of secondary antibody staining described above, samples stained with primary FBXO11 (mouse) and LONP1 (rabbit) antibodies were incubated with Anti-Rabbit PLUS (Sigma-Aldrich DUO92002) and Anti-Mouse MINUS (Sigma-Aldrich DUO92004) *in situ* probes, ligated and amplified using red detection reagents (Sigma-Aldrich, DUO92008).

After final washes following Alexa fluor-conjugated antibody staining or PLA, all samples were co-immunostained with Alexa Fluor 647 conjugated TOM20 antibody (1:1000, Abcam ab209606) for 2 hours at room temperature to identify mitochondria. All samples were then mounted on slides with ProLong Gold Antifade Mountant with DAPI (Thermo Fisher P36935).

All samples were imaged on a Leica SP5 laser scanning confocal on 63X/1.4 oil objective and processed in ImageJ (Fiji). Colocalization was determined by measuring the area of fluorescence for the indicated proteins and their overlap area in single cells using composite RGB images.

Mitochondria Fractionation and isolation by differential centrifugation

Mitochondria were isolated from *FBXO11*-KO and OCI-AML3 cells as described (3), with the protocol scaled down for 1 x 10^8 cells. Briefly, cells were pelleted and washed with PBS before being swollen for 10 minutes with hypotonic buffer and ruptured by using a Dounce homogenizer. The homogenized supernatant was centrifuged 3 times at 1300 x g for 5 minutes, and then at 17,000 x g for 15 minutes to pellet the mitochondria. Mitochondria were washed once and pelleted again, before being lysed with RIPA buffer as described above.

Identifying amino acids of LONP1 responsible for interacting with SCF^{FBXO11} by AlphaFold3

Structures of protein complexes containing FBXO11, SKP1, CUL1 and RBX11 together with LONP1 were predicted using AlphaFold 3 (4). To identify residues involved in the interface of two proteins, the solvent accessible surface area (SASA) of each residue was calculated using the Gromacs (5) (version 2018.1) implementation of the double cube lattice method (6). The difference between the SASA of a given amino acid in the presence of all proteins and when one of the proteins is excluded from the calculation gives the contribution of that protein to the occlusion of that amino acid. To combine SASA results from multiple predicted models, a weighted average, ⟨ΔSASA⟩ was calculated using ipTM scores of the models as weights. The available structures were visualized in UCSF ChimeraX (7). To enable visual comparisons of relative orientations, the SKP1 chain was aligned across all models. Residues 1-150 on FBXO11 and 1-23 on RBX1 were observed to be disordered and were omitted from visual representations for clarity. These disordered residues are consistent with predictions from IUPred3 (8).

Generating LONP1 deletion mutant constructs

Specific LONP1 amino acids were selected for deletion based on each amino acid's SASA score after docking with the SCF^{FBXO11} complex. Primers to generate deletions from our pWPT-LONP1-FLAG-E2A-mcherry vector were designed using Takara Bio's primer design tool for deletion mutagenesis. Cloning of the deletion mutants was performed using In-Fusion Snap Assembly (Takara Bio, 638945) per the manufacturer's protocol.

Seahorse XF Cell MitoStress Test

FBXO11-KO clone cells were in the growth phase at the time of the assay. The cells were counted and seeded at 80,000 cells per well in the 96-well Agilent Seahorse XF Cell Mito Stress Test (Agilent

103010-100) assay plate. The assay was performed as outlined in the manufacturer's protocol and run on the Seahorse XFe bioanalyzer. Oligomycin was added at 20 min, Carbonyl cyanide 4-(trifluoromethoxy)phenylhydrazone (FCCP) at 40 min, and rotenone and antimycin A at 60 min. Analysis and interpretation were done according to the manufacturer's protocol.

Measuring mitochondrial membrane potential

Mitochondrial membrane potential (MMP) was measured by TMRE (Cedarlane labs ENZ-52309). Cells were cultured in their regular culture media and conditions with 400 nM TMRE for 30 min. Cells were spun down at 400 x g for 5 min to remove the media and TMRE, and the cell pellets were resuspended in PBS with 2% FBS and 1 µg/mL DAPI and kept on ice before analysis by flow cytometry. Stained cells were resuspended in PBS with 2% FBS and 1 µg/mL DAPI for flow cytometry analysis.

Mitochondrial mass measurements

MitoTracker™ Deep Red FM (Fisher Scientific M22426) was used to measure mitochondrial mass in HSPC. Cell staining was performed according to the manufacturer's protocol. Cells were stained for 30 min, then washed with PBS with 2% FBS and resuspended in PBS with 2% FBS and 1 µg/mL DAPI for flow cytometry analysis.

Native gel electrophoresis

Mitochondria were isolated by differential centrifugation from K562 *FBXO11*-KO reconstituted with empty vector or FLAG-FBXO11, or OCI-AML3 cells expressing shFBXO11 or shLONP1 demonstrated to induce respiratory chain defect signatures in CD34$^+$ hematopoietic stem and progenitor cells (HSPC, Fig 6c). Mitochondrial proteins were extracted from mitochondria pellets with digitonin (Invitrogen, BN2008) and separated on a 3-12% bis-tris poly acrylamide gel (Fisher Scientific, BN1001BOX) as described (9) before transferring for 16hours at 30V onto a PVDF membrane. Electron transport chain complexes were identified by blotting with an anti-OXPHOS antibody cocktail (Abcam, ab110411).

Ubiquitin, neddylation activating enzyme, and proteasome inhibitor treatment

Ubiquitin activating enzyme (UAE) inhibitors PYR-41 (Sigma-Aldrich N2915-5MG) was used at 50 nM, and TAK-243 (also known as MLN7243, Selleck Chemicals S8341) at 0.1 µM. Neddylation activating enzyme (NAE) inhibitors MLN4924 (Cayman Chemical Company 15217) was used at 50 nM, and TAS4464

(Selleck Chemicals 15217) was used at 50nM. Proteasome inhibitors MG132 (ApexBio A2585) was used at 1µM, and Bortezomib (Selleck Chemicals S1013) at 50nM. Concentrations were determined by published concentrations used on K562 cells or other myeloid cell lines, and inhibitory effect on global ubiquitination or neddylation levels were confirmed by western blot. Cells were seeded at 300k cells/mL and treated with the above inhibitors added directly to culture media for 16 hours. Inhibitor stock solutions were prepared in DMSO.

RNAseq analysis

RNAseq read alignment was performed using STAR aligner, and count generation was done with Salmon. Variance stabilizing transformation was performed to allow comparison between samples. Transcript per million (TPM) values were filtered to remove genes that have low counts (TPM < 0.5, equivalent to a count of 10 for the dataset) for at least 2 samples. Differential gene expression analysis was performed on the RNAseq data using DeSeq2 package with R. Gene Set Enrichment Analysis (GSEA) was performed using the Hallmark, C2, and C5 gene set collections from MSigDB. GSEA was performed using pre-ranked gene lists for each comparison presented, where the gene list was ranked based on the log2 fold change value. CellRadar plots from Figure 7 were generated using the public user interface provided by the Karlsson lab (https://karlssong.github.io/cellradar/). The same definition of differentially expressed genes was used, and HSPC data was taken from the BloodSpot HemaExplorer human normal hematopoiesis dataset.

Deconvolution of AML sample hierarchies from bulk RNAseq and stratification by *FBXO11* expression

Deconvolution of 864 AML sample hierarchies were performed using the original samples and code provided by Zeng et al. (10) (https://github.com/andygxzeng/AMLHierarchies). Following deconvolution and projection by Principal Component Analysis (PCA) patients were stratified into tertiles within their datasets (TCGA, Beat AML, Leucegene) based upon their *FBXO11* expression, and their tertile identifier were projected onto the PCA. The sample's original hierarchy from Zeng et al. was identified for each *FBXO11* expression tertile.

Histology

Mouse brain samples were fixed in 10% buffered formalin and stored at 4°C for a week before paraffin embedding. H&E and myeloperoxidase staining of brain samples were performed at the BC

Cancer Vancouver Center Pathology department. Bone marrow smears were stained with Wright-Giemsa staining solution.

Calculating $CD34^+$ cell output for xenotransplants

The number of human $CD34^+$ cells generated per input $CD34^+$ cell after the LTC-IC and xenotransplant process was calculated based on the assumption below that: the ratio of the number of $CD34^+$ cells harvested from the LTC (**A**) to the number of $CD34^+$ cells that were injected into the mice (**B**) equaled the number of $CD34^+$ cells that would have been harvested at xenotransplant endpoint if the total amount of $CD34^+$ cells harvested from the LTC were injected into the mice(**C**) to the number of $CD34^+$ cells that were actually harvested at xenotransplant endpoint (**D**). Since equal numbers (100,000) of cells harvested from LTC were injected into recipient mice for xenotransplantation, the number of $CD34^+$ transplanted varied between conditions (Supplemental Table 6).

To calculate the number of $CD34^+$ cells generated by the end of the experiment per $CD34^+$ cell input into the LTC-IC experiment, we divided "X" by the number of LTC-IC input $CD34^+$ cells, which was the same for each condition.

$$\frac{A}{B} = \frac{C}{D}$$

$$\frac{\#CD34^+ \text{ cells at LTC harvest}}{\text{In vivo } CD34^+ \text{ input cells injected}} = \frac{X}{\text{Average } \#CD34^+ \text{ cells in BM at endpoint}}$$

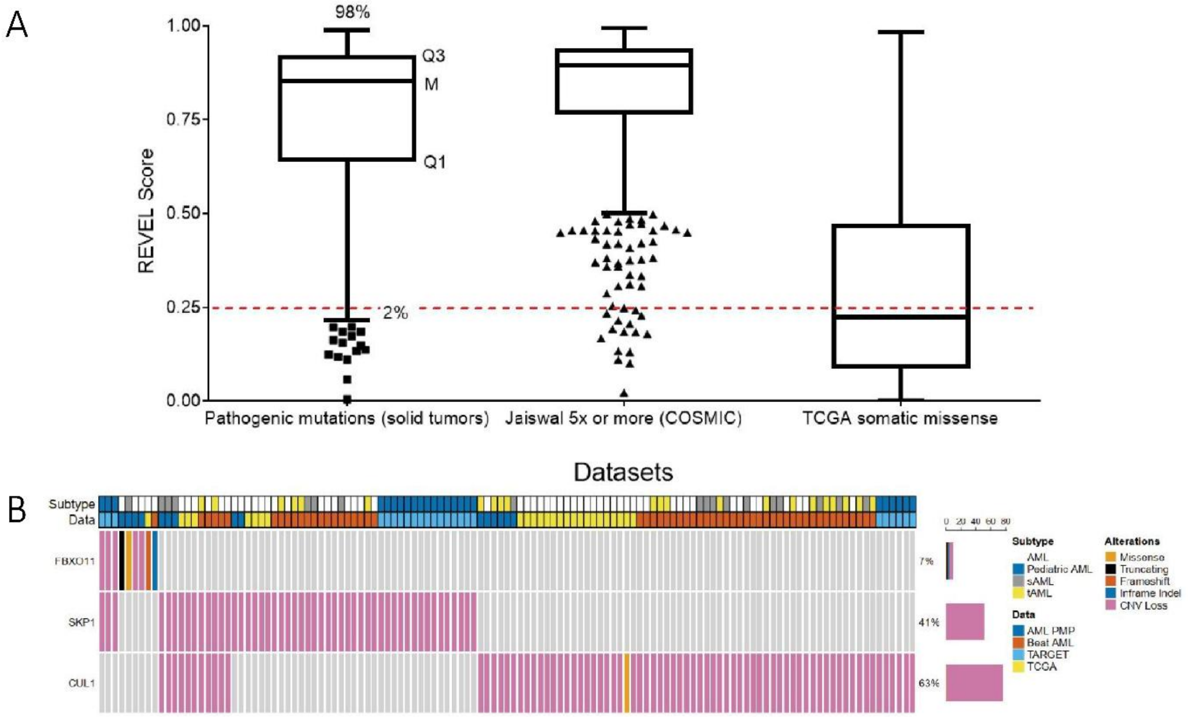

Supplemental Figure 1. Mutations affecting the ubiquitin pathway occur frequently in AML.
A, REVEL scores for different mutation datasets were compared to identify a threshold for pathogenicity prediction. Mutations plotted include: (1) Known actionable pathogenic missense variants in solid tumors (*n* = 146 variants). (2) Pathogenic variants associated with human hematological cancers (Jaiswal) ((11) (*N* = 149 variants). (3) Somatic missense mutations in AML identified in TCGA (12) (*N* = 1233 mutations). Applying a minimal REVEL threshold of 0.250, represented by the red dotted horizontal line, removes more than half of all identified somatic missense TCGA variants, while retaining 98% of known pathogenic variants. **B**, Single nucleotide, indel and copy number variants (CNV) affecting the SCF^{FBXO11} complex are summarized in the oncoprint. In the combined AML PMP, TCGA, Beat AML and TARGET pediatric AML datasets, 8.7% of adult (91/1062) and 7.1% of all 1727 AML patients (123/1727) and had a mutation or copy loss of a gene affecting the SCF^{FBXO11} complex.

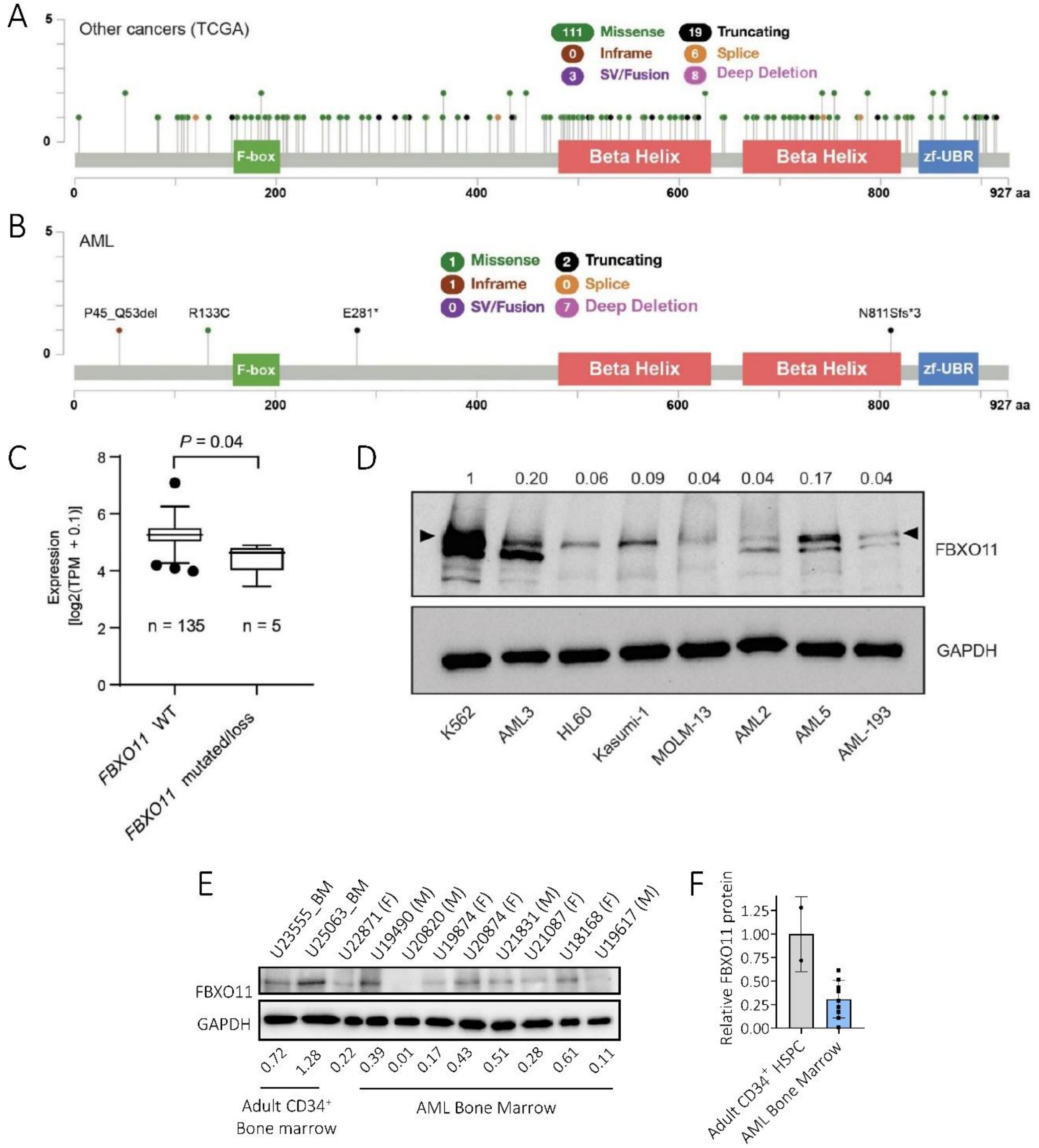

Supplemental Figure 2. *FBXO11* is recurrently mutated in multiple cancers.
A, *FBXO11* mutation types, locations, and frequencies identified in all TCGA cancer samples (n = 10,753) excluding AML samples are shown (cBioPortal). **B**, *FBXO11* mutation types, locations, and frequencies identified in TCGA, Beat AML, TARGET pediatric AML, and AML PMP samples. **C**, *FBXO11* transcript expression in AML PMP samples with mutated/deleted (*FBXO11* mutated/loss) or WT *FBXO11* represented as a box and whisker plot. **D**, Indicated myeloid cell lines were immunoblotted for FBXO11 expression. **E**, FBXO11 expression of purified $CD34^+$ adult bone marrow HSPC compared to the same 9 AML bone marrow samples presented in **Figure 2C**, normalized to GAPDH. **F**, Quantification of FBXO11 levels for samples presented in (**E**). *P* values represent two-tailed *t*-tests and error bars represent s.d.

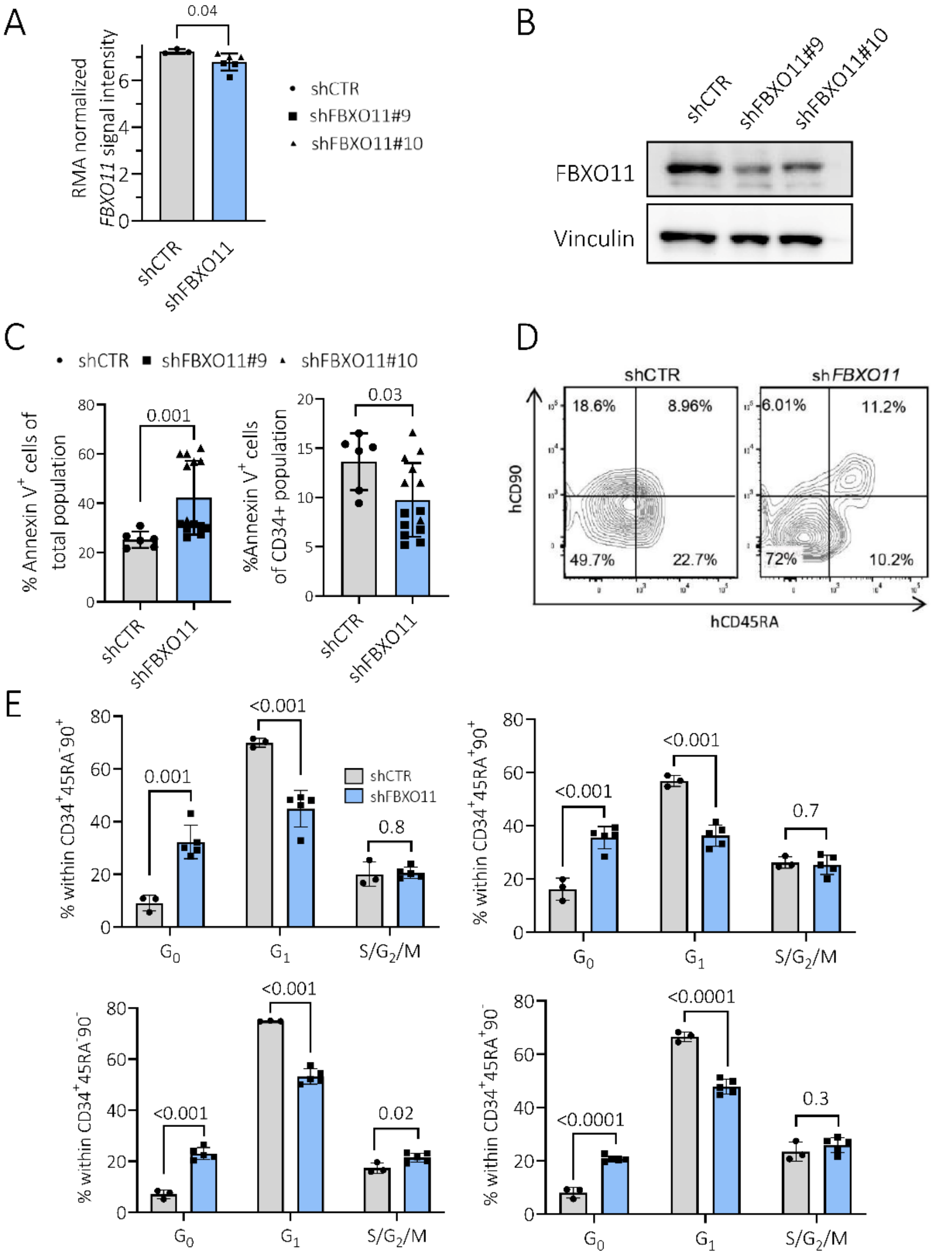

Supplemental Figure 3. CD34⁺ HSPC with *FBXO11* depletion have reduced cell death.
A, Robust multiarray averaging (RMA)-normalized *FBXO11* signal intensity in CD34⁺ HSPC samples (*N* = 3). **B**, Validation of FBXO11 protein depletion by shRNA in K562 cells. **C**, The Annexin V⁺ population within total (left) or CD34⁺ (right) cells was analyzed by flow cytometry (*N* = 6 (shCTR), *N* = 7 (shFBXO11)). **D**, Representative plot of cultured CD34⁺ HSPC analyzed for expression of long-term and short-term HSC ($CD90^{+/-}CD45RA^{-}$) or hematopoietic progenitor ($CD90^{-}CD45RA^{+}$) markers. **E**, Cell cycle states of the indicated HSPC populations were determined by Ki-67 and DAPI staining (*N* = 3 (shCTR), *N* = 5 (shFBXO11)). *P* values represent two-tailed *t*-tests and error bars represent s.d.

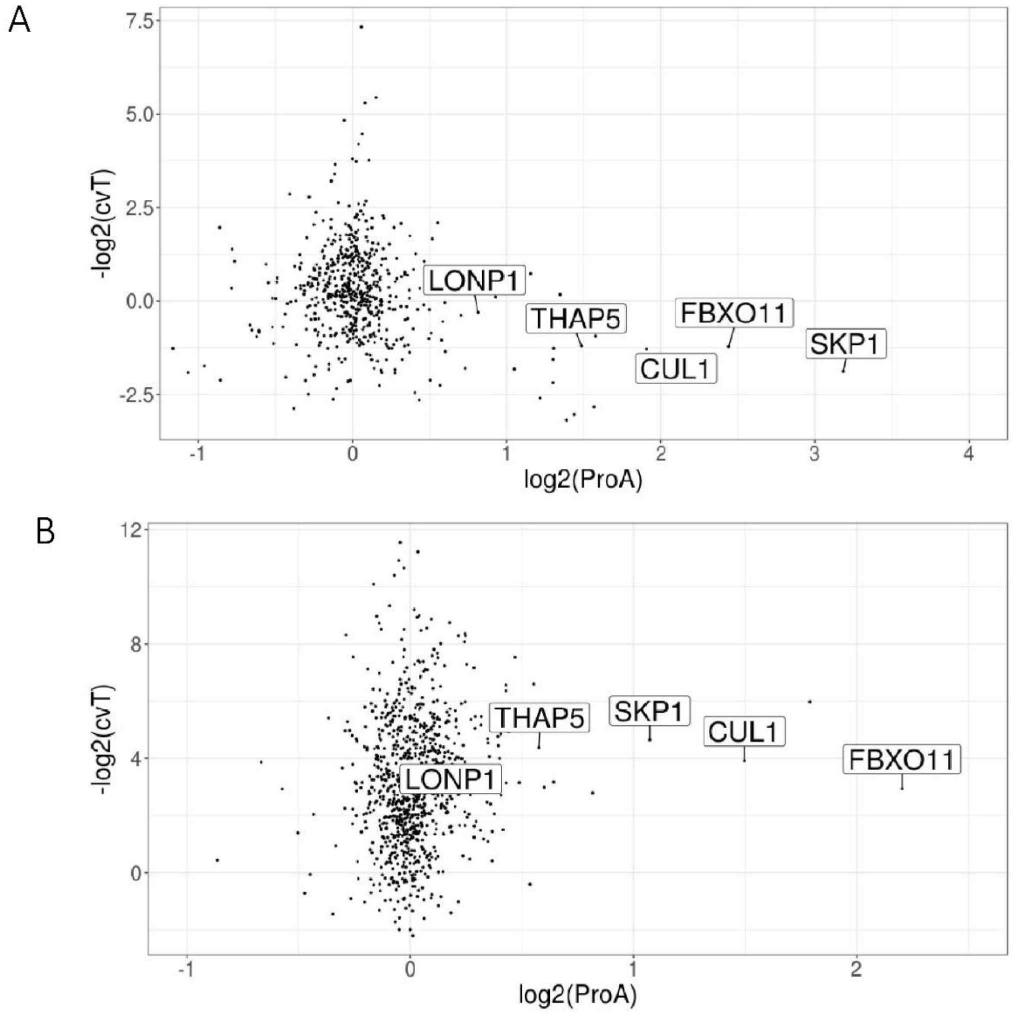

Supplemental Figure 4. Proteins co-immunoprecipitating with FLAG-FBXO11.

Mean protein abundance ($\log_2$(ProA)) between replicates and transformed coefficient of variation (cvT) of FLAG-FBXO11 co-immunoprecipitated proteins (**A**), without endonuclease (*N* = 3) and (**B**), with endonuclease (*N* = 4), detected by quantitative tandem mass spectrometry. All detected proteins are plotted with the targets detected in both experimental conditions are labelled.

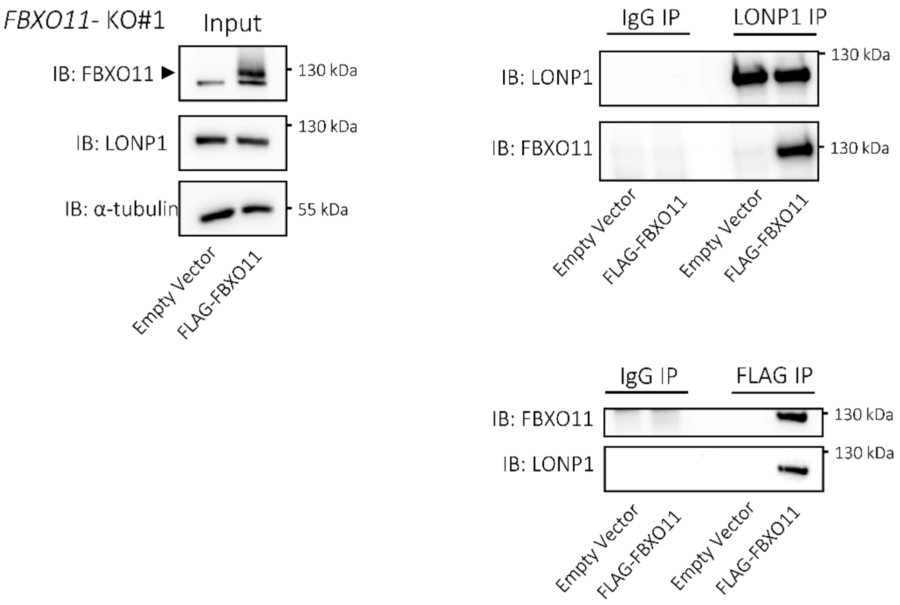

Supplemental Figure 5. **LONP1 reciprocally co-immunoprecipitates with FBXO11.**
Co-immunoprecipitation (IP) of LONP1 by LONP1 IP or FLAG-FBXO11 by FLAG IP in *FBXO11*-KO K562 cells expressing empty vector or FLAG-FBXO11 followed by immunoblotting (IB) for LONP1 and FBXO11.

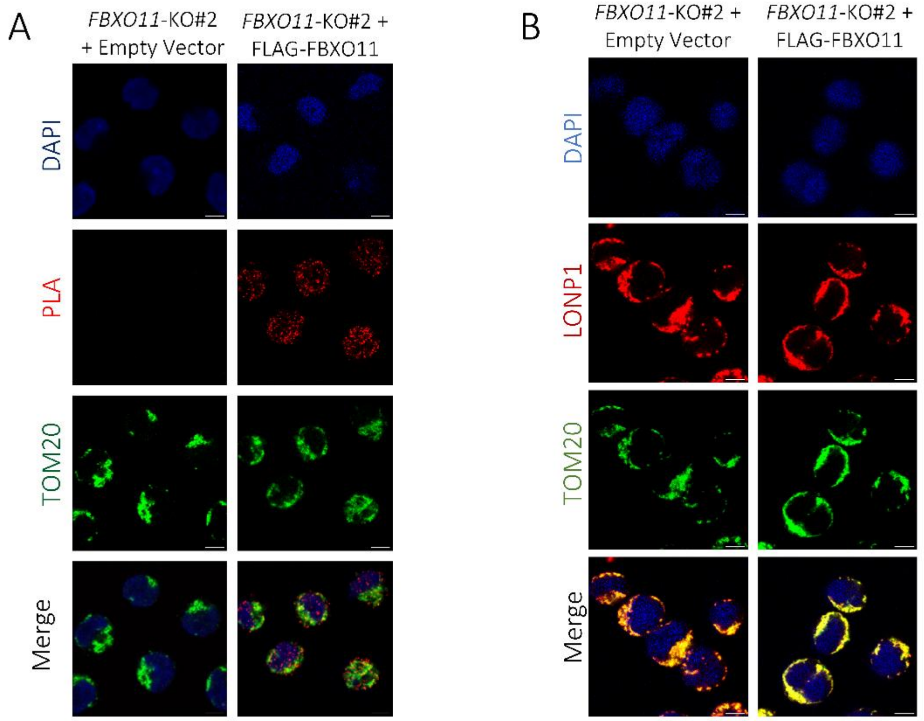

Supplemental Figure 6. FBXO11 facilitates LONP1 localization in *FBXO11*-KO#2 cells.
Representative images of (**A**) FBXO11-LONP1 proximity ligation assay quantified in **Figure 4D** and (**B**) LONP1 mitochondrial localization in *FBXO11*-KO#2 expressing empty vector or FLAG-FBXO11 quantified in **Figure 4F**.

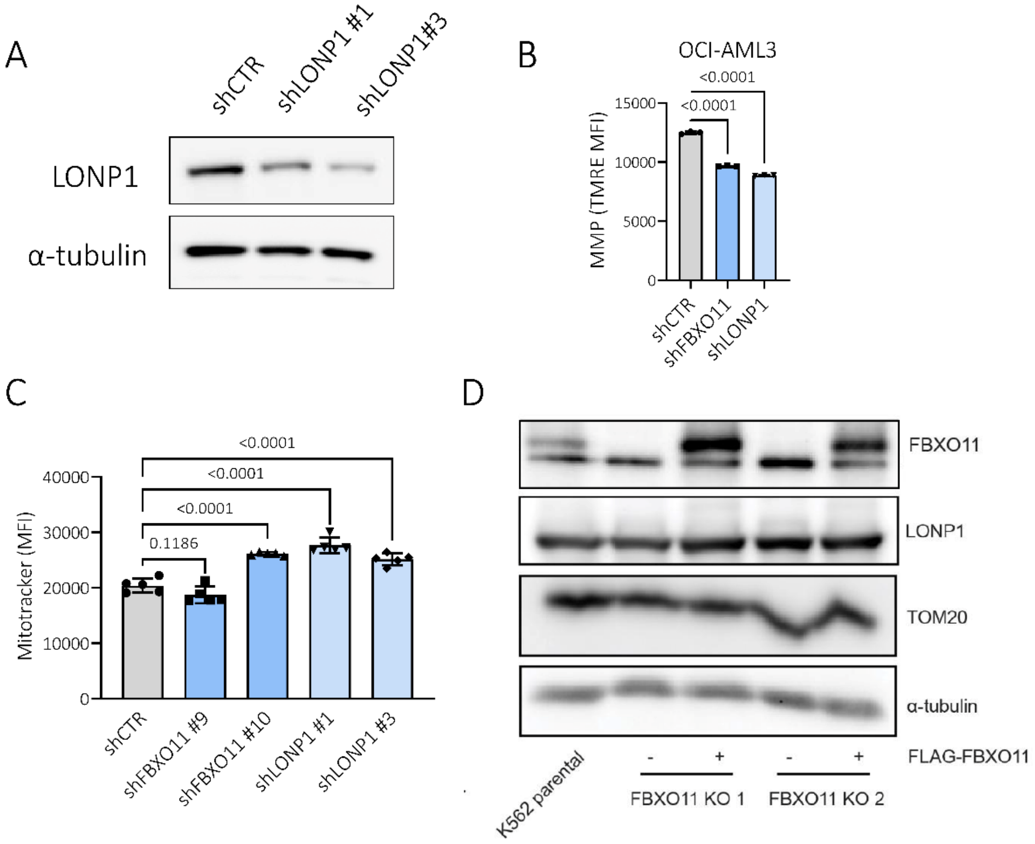

Supplemental Figure 7. **Mitochondrial mass does not decrease with *FBXO11* or *LONP1* knockdown.**
A, LONP1 protein abundance in shLONP1 K562 cells. **B**, MMP measured in OCI-AML3 cells expressing a non-targeting control (shCTR), shFBXO11 or shLONP1 (N = 3). **C**, Mitochondrial mass of $CD34^{+}$ HSPC expressing a non-targeting shCTR or shFBXO11 or shLONP1 measured using the MitoTracker dye by flow cytometry (N = 5). P values represent one-way ANOVA with Dunnett's test, error bars represent s.d. **D**, Whole cell lysates from K562 parental cells (K562) or *FBXO11*-KO cells expressing empty vector or FLAG-FBXO11 immunoblotted for TOM20.

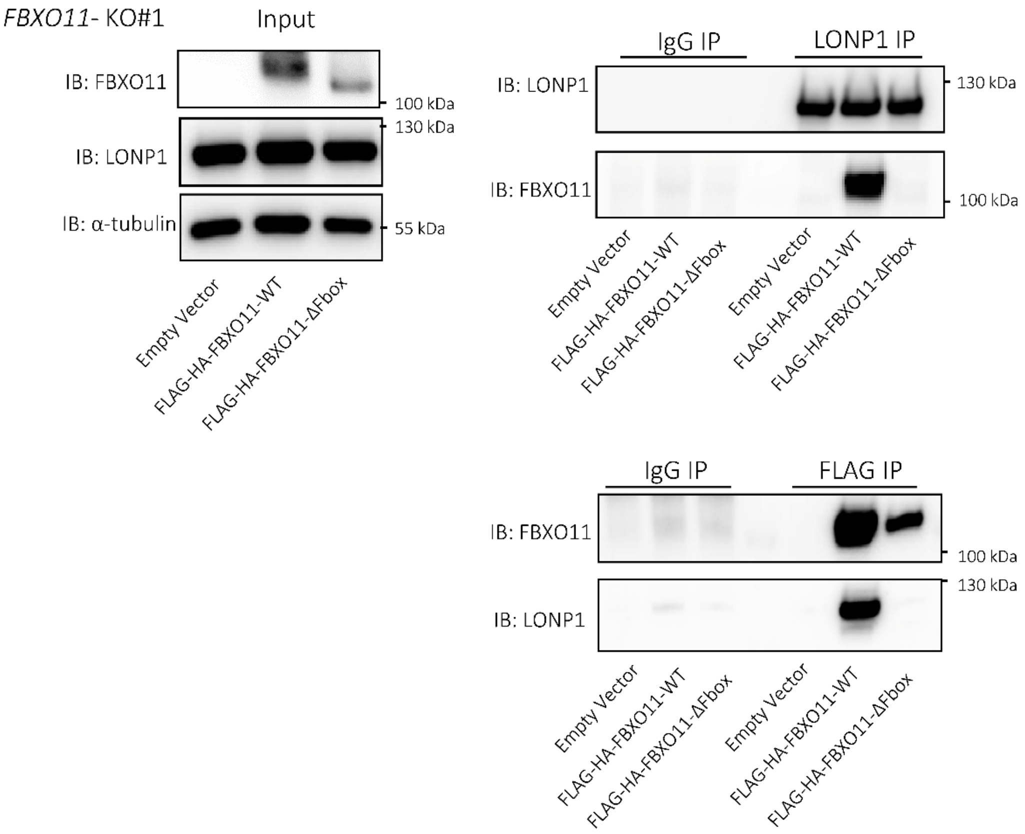

Supplemental Figure 8. FBXO11-ΔFbox does not interact with LONP1.
Whole cell lysates from K562 *FBXO11*-KO cells expressing empty vector, WT FLAG-*FBXO11*, or an *FBXO11* mutant lacking the F-box domain (*FBXO11*-ΔFbox) was immunoprecipitated with LONP1 or FLAG-tag antibody and the immunoprecipitated proteins or whole cell lysates (Input) were immunoblotted (IB) for FBXO11, FLAG, and α-tubulin.

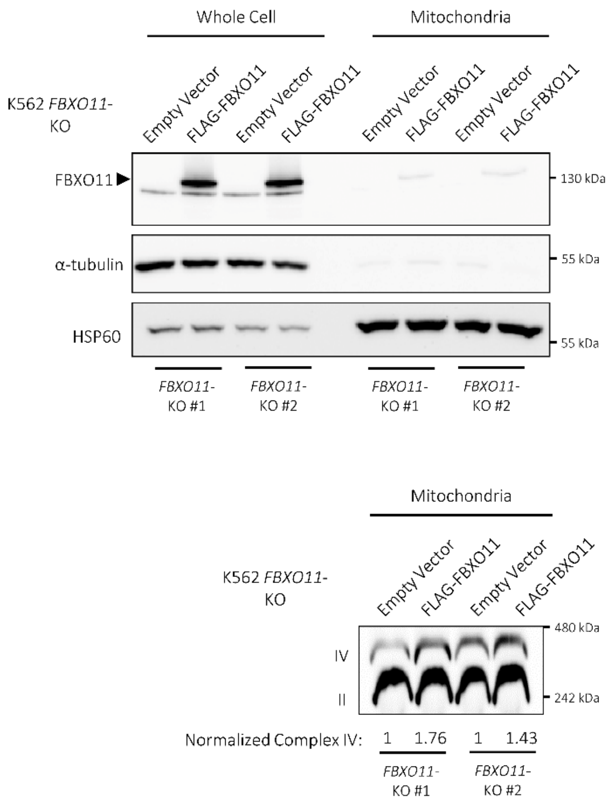

Supplemental Figure 9. Complex IV assembly is reduced in *FBXO11*-KO cells.

Immunoblot for FBXO11 expression in whole cell and mitochondrial lysates from K562 *FBXO11*-KO cells transduced with empty vector or FLAG-FBXO11 (N = 2). Assembled electron transport chain complexes assayed by digesting equal amounts of isolated mitochondria in digitonin and performing blue native gel electrophoresis followed by immunoblotting. Mitochondria were immunoblotted with α-tubulin and HSP60 to confirm mitochondrial purity. Quantification represents mean of 2 independent experiments.

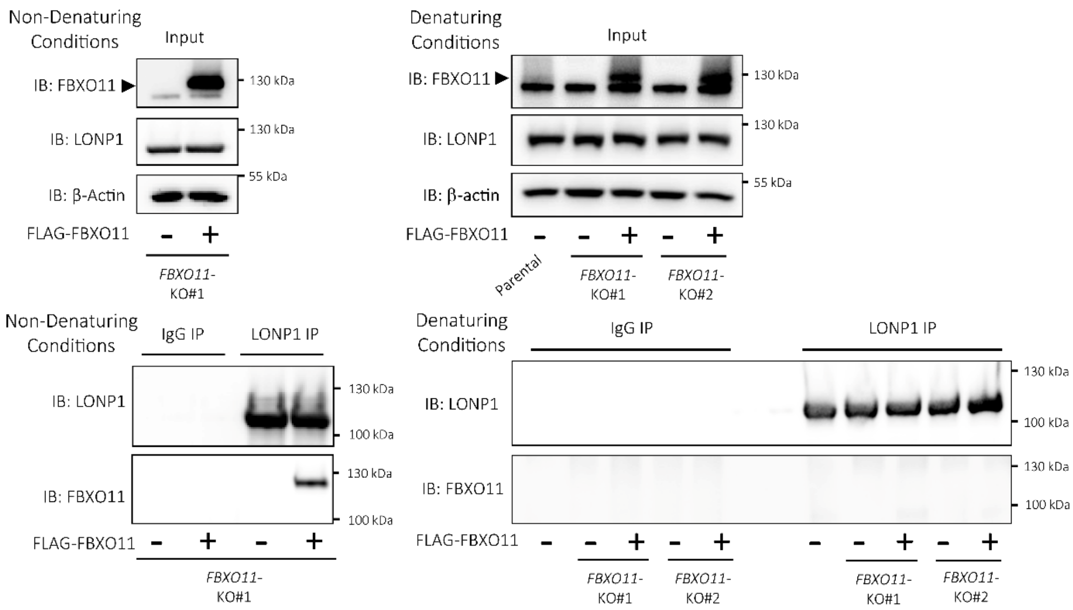

Supplemental Figure 10. FBXO11 and LONP1 do not interact under denaturing conditions used to identify LONP1 K63-Ub.

LONP1 IP followed by immunoblotting under denaturing lysis conditions to identify LONP1-specific post-translational modifications in **Figure 6K**. Note lack of interaction with FBXO11 under denaturing conditions.

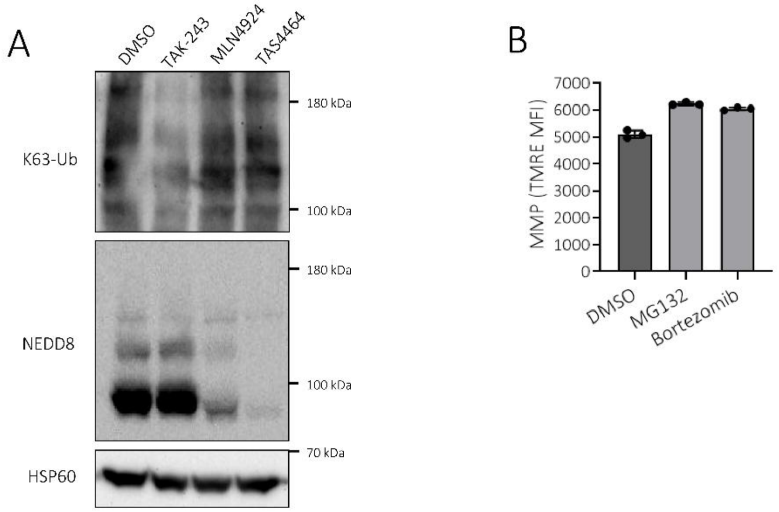

Supplemental Figure 11. Treatment with neddylation inhibitors does not block global K63-Ub.

A, Immunoblot for global K63-linked polyubiquitination (K63-Ub) and neddylation (NEDD8) following neddylation inhibitor treatment. Lysates were collected from K562 cells treated for 16-hours with DMSO, TAK-243 (0.1 µM) as a positive control, or neddylation inhibitors MLN4924 (50 nM) and TAS4464 (50 nM). **B**, MMP measured in K562 cells expressing FLAG-FBXO11 treated with vehicle (DMSO), or proteasome inhibitors (MG132 or Bortezomib) (*N* = 3).

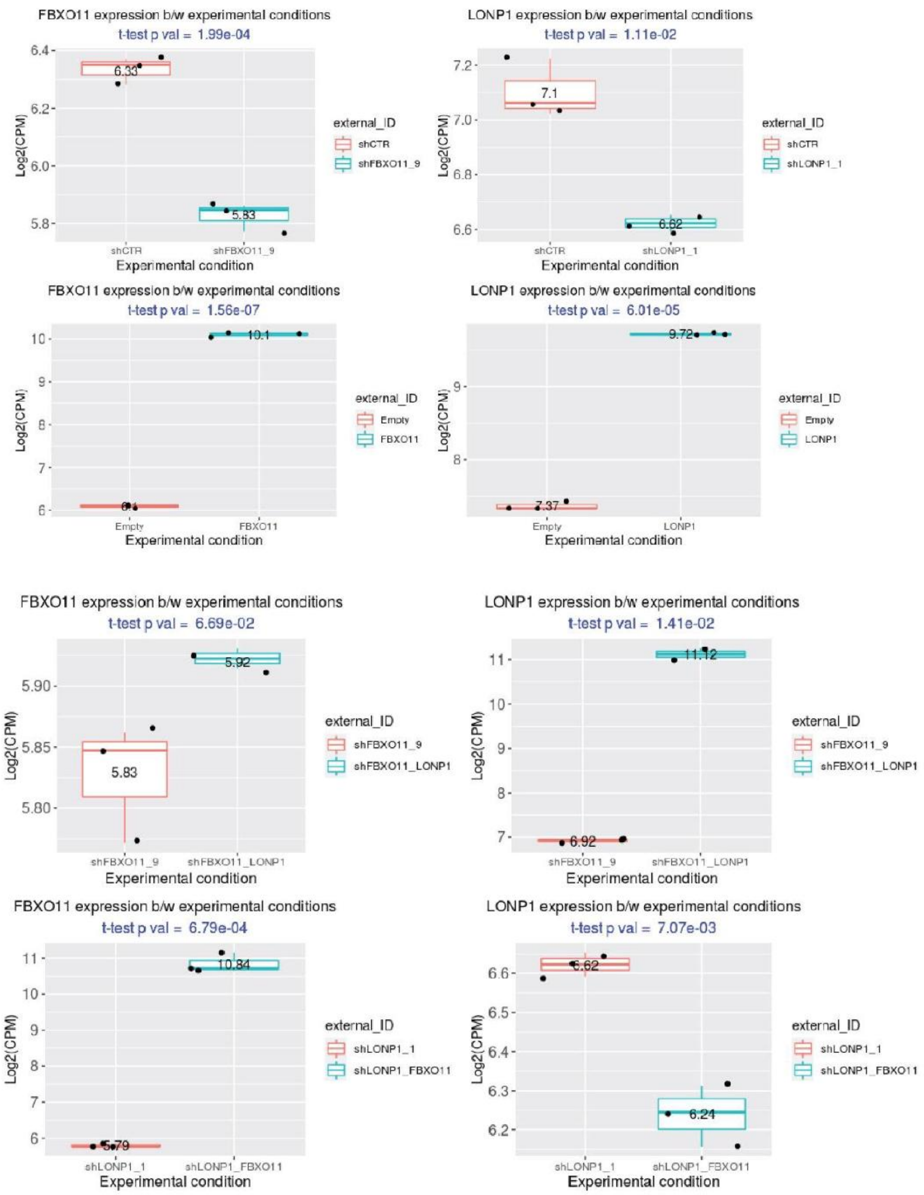

Supplemental Figure 12. **Confirmation of *FBXO11* and *LONP1* transcript over-expression and/or knockdown in RNA-seq analysis.**

Transcript expression of indicated genes expressed as log_2(CPM) (counts per million reads mapped) between (b/w) indicated experimental conditions in the CD34$^+$ HSPC RNA-seq experiment.

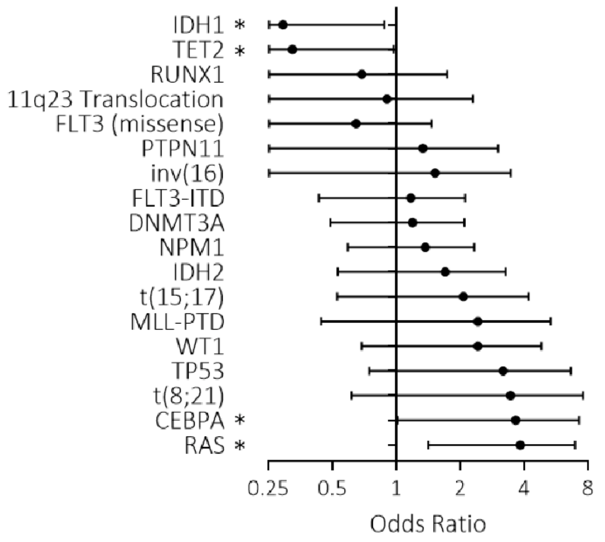

Supplemental Figure 13. **Ubiquitin pathway mutations co-occur with *RAS* and *CEBPA* mutations.** Shown are odds ratios of AML-associated mutations for AML PMP samples occurring in ubiquitin pathway-mutated samples compared to samples that are wild-type for ubiquitin pathway genes. Mutations with odds ratio > 1 are likely to co-occur with ubiquitin pathway mutations, and odds ratios < 1 are likely to occur mutually exclusively with ubiquitin pathway mutations. *P* values determined by Fisher's exact test, * *P*<0.05, bars represent 95% confidence intervals.

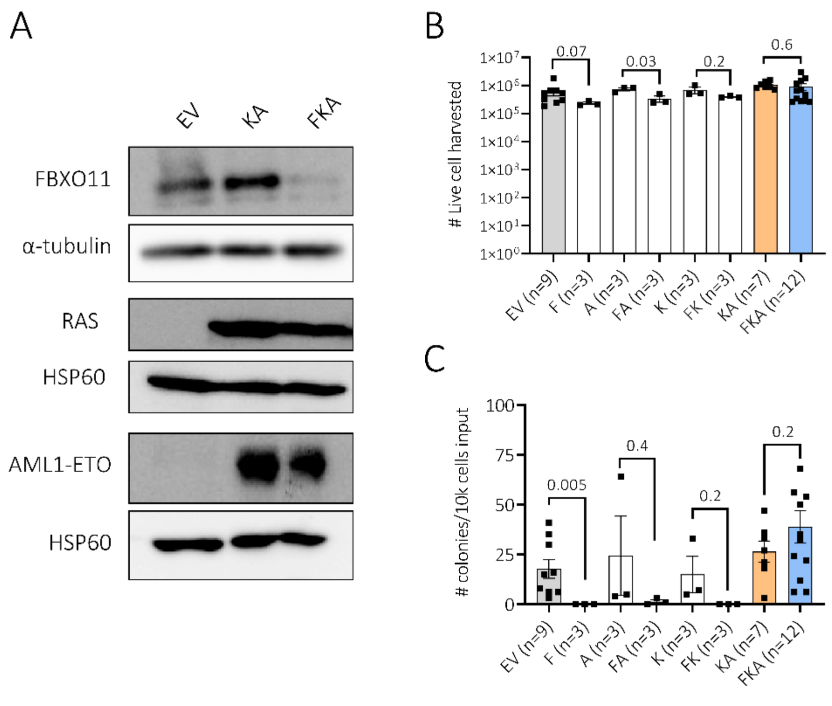

Supplemental Figure 14. Quantification of total cell numbers and CFCs in shFBXO11-, *AML1-ETO*- and *KRAS*^{G12D}- transduced CD34^{+} HSPC harvested after long-term culture.

A, Protein abundance of FBXO11, RAS, and AML1-ETO in xenotransplanted combinations after transduction. **B**, Total number of live cells harvested from long-term culture were counted using trypan blue staining. **C**, CFCs were counted on day 17 after plating (*N* = 3-12). *P* values represent two-tailed *t*-tests and error bars represent s.d. EV = Empty vector and control shRNA; F = shFBXO11*;* A = AML1-ETO; K = KRAS^{G12D}; FA = F + A; KA = K + A; FKA = F + K + A.

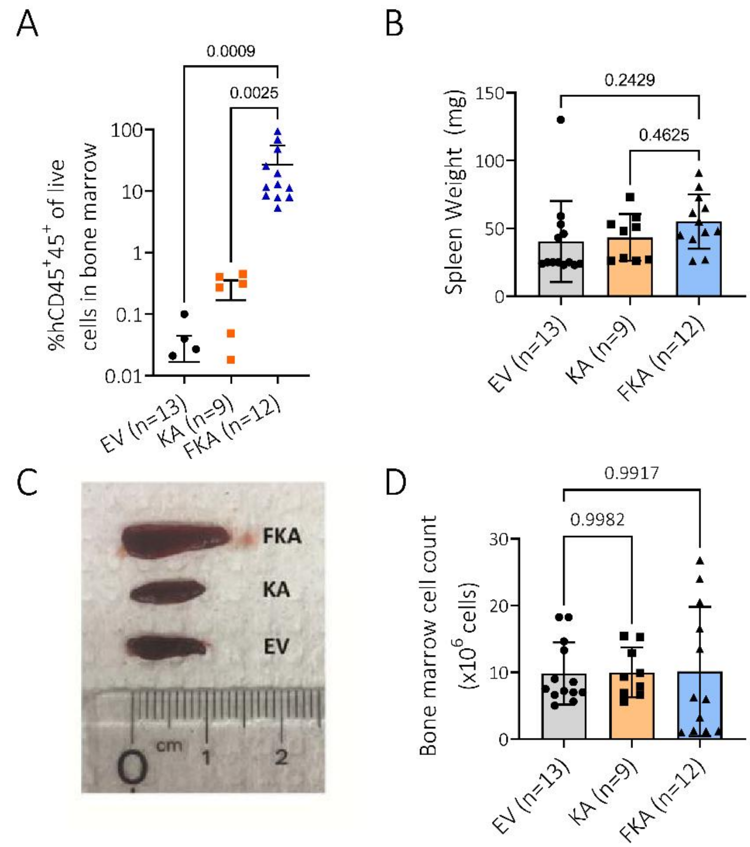

Supplemental Figure 15. Analysis of bone marrow and spleen cells from primary xenotransplanted mice at endpoint.

A, Percentages of double $CD45^+$ human hematopoietic cells using two distinct antibody clones in bone marrow at endpoint. **B**, Spleen weights were measured at endpoint (*N* = 9-13 mice). **C**, Representative spleens are shown. **D**, Total live bone marrow cell counts at endpoint (*N* = 9-13 mice). *P* values represent one-way ANOVA with Dunnett's test, error bars represent s.d.

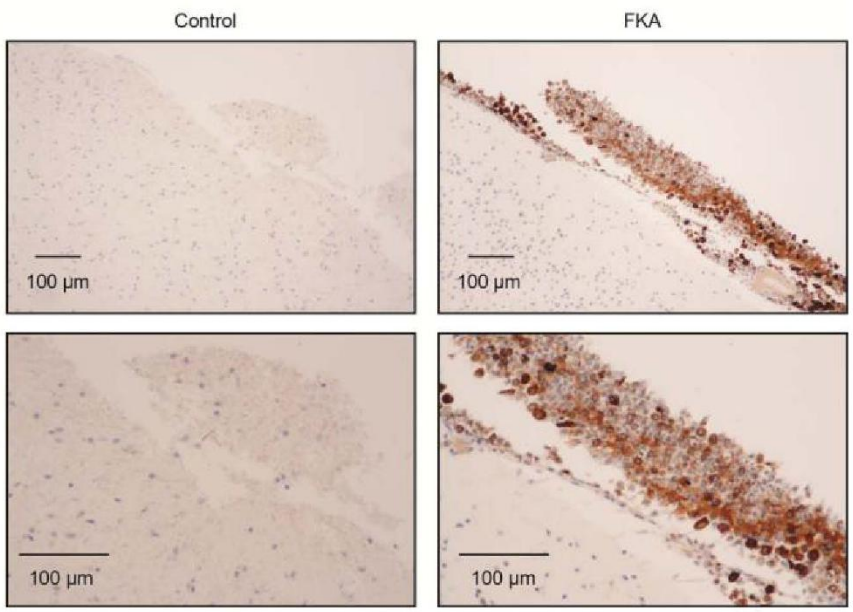

Supplemental Figure 16. Myeloid cell infiltration in the leptomeninges of FKA mice.
Myeloperoxidase stains performed on brain sections of a wild-type NRG-3GS mouse (Control) and an FKA mouse, showing myeloid cell infiltration.

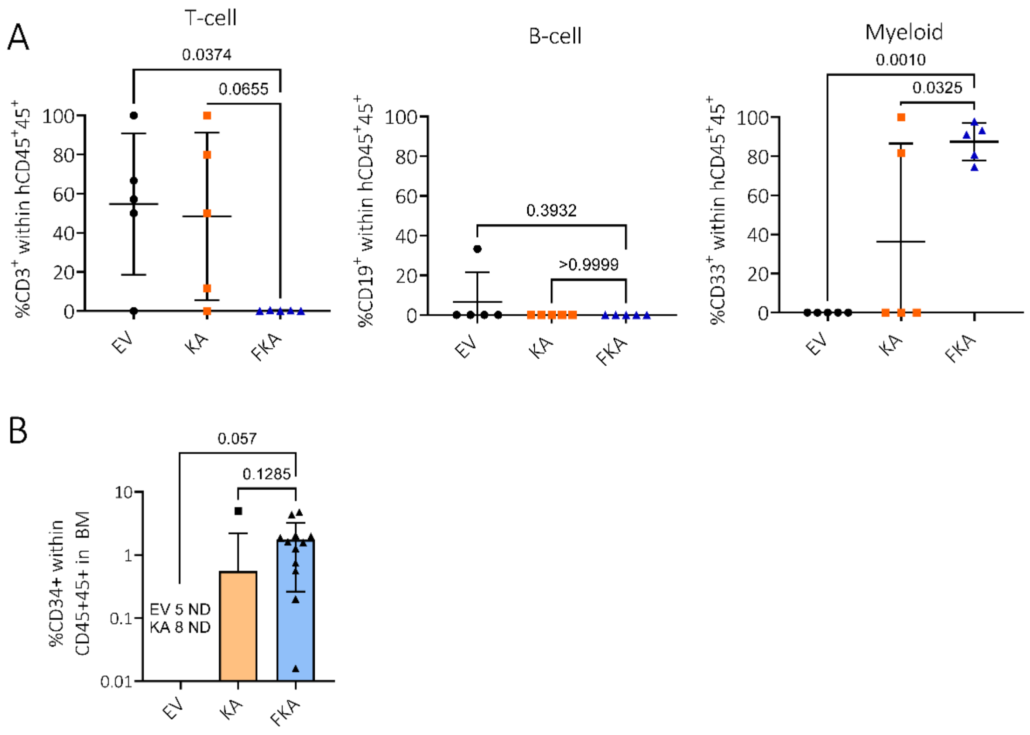

Supplemental Figure 17. Flow cytometry analysis of bone marrow cells harvested at endpoint.
A, Flow cytometry analysis of lymphoid and myeloid cell engraftment at endpoint following xenotransplantation (*N* = 5 mice). *P* values represent two-tailed *t*-tests and error bars represent s.d. **B**, Percentages of $CD34^+$ cells within the engrafted human hematopoietic cell population in bone marrow at endpoint (*N* = 5-12 mice, ND = not detected. *P* values represent one-way ANOVA with Dunnett's test, error bars represent s.d.

Supplemental References

1. Griffin NM, et al. Label-free, normalized quantification of complex mass spectrometry data for proteomic analysis. *Nat Biotechnol*. 2010;28(1):83–89.

2. Lavallée-Adam M, et al. PSEA-Quant: A Protein Set Enrichment Analysis on Label-Free and Label-Based Protein Quantification Data. *J Proteome Res*. 2014;13(12):5496–5509.

3. Clayton DA, Shadel GS. Isolation of Mitochondria from Tissue Culture Cells. *Cold Spring Harb Protoc*. 2014;2014(10):pdb.prot080002.

4. Abramson J, et al. Accurate structure prediction of biomolecular interactions with AlphaFold 3. *Nature*. 2024;630(8016):493–500.

5. Berendsen HJC, Van Der Spoel D, Van Drunen R. GROMACS: A message-passing parallel molecular dynamics implementation. *Comput Phys Commun*. 1995;91(1–3):43–56.

6. Eisenhaber F, et al. The double cubic lattice method: Efficient approaches to numerical integration of surface area and volume and to dot surface contouring of molecular assemblies. *J Comput Chem*. 1995;16(3):273–284.

7. Pettersen EF, et al. UCSF ChimeraX: Structure visualization for researchers, educators, and developers. *Protein Sci*. 2021;30(1):70–82.

8. Erdős G, Pajkos M, Dosztányi Z. IUPred3: prediction of protein disorder enhanced with unambiguous experimental annotation and visualization of evolutionary conservation. *Nucleic Acids Res*. 2021;49(W1):W297–W303.

9. Jha P, Wang X, Auwerx J. Analysis of Mitochondrial Respiratory Chain Supercomplexes Using Blue Native Polyacrylamide Gel Electrophoresis (BN-PAGE). *Curr Protoc Mouse Biol*. 2016;6(1):1–14.

10. Zeng AGX, et al. A cellular hierarchy framework for understanding heterogeneity and predicting drug response in acute myeloid leukemia. *Nat Med*. 2022;28(6):1212–1223.

11. Jaiswal S, et al. Age-Related Clonal Hematopoiesis Associated with Adverse Outcomes. *N Engl J Med*. 2014;371(26):2488–2498.

12. Genomic and Epigenomic Landscapes of Adult De Novo Acute Myeloid Leukemia. *N Engl J Med*. 2013;368(22):2059–2074.
