# Supplementary material for: Loss of FBXO11 establishes a stem cell program in acute myeloid leukemia by dysregulating LONP1
Source: J Clin Invest. 2025 Nov 25;136(2):e181943. doi: 10.1172/JCI181943 (PMC12807481; doi:10.1172/JCI181943)

Full unedited blots – Figure 2c

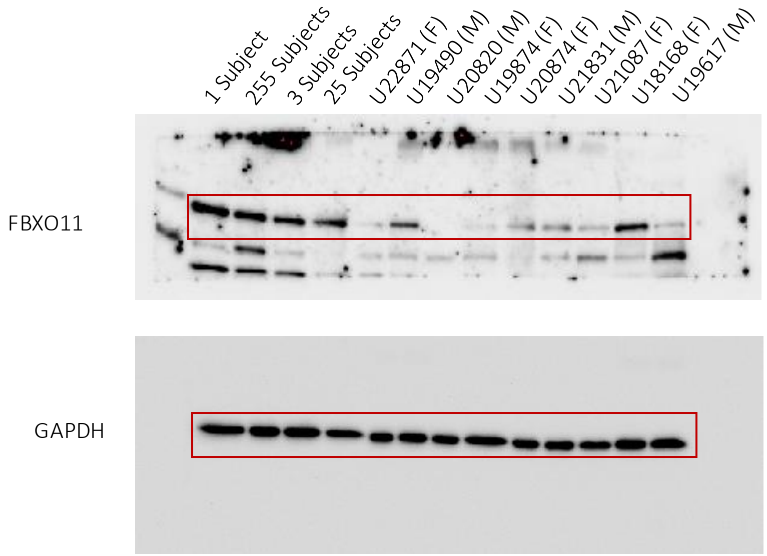

Full unedited blots – Figure 4d

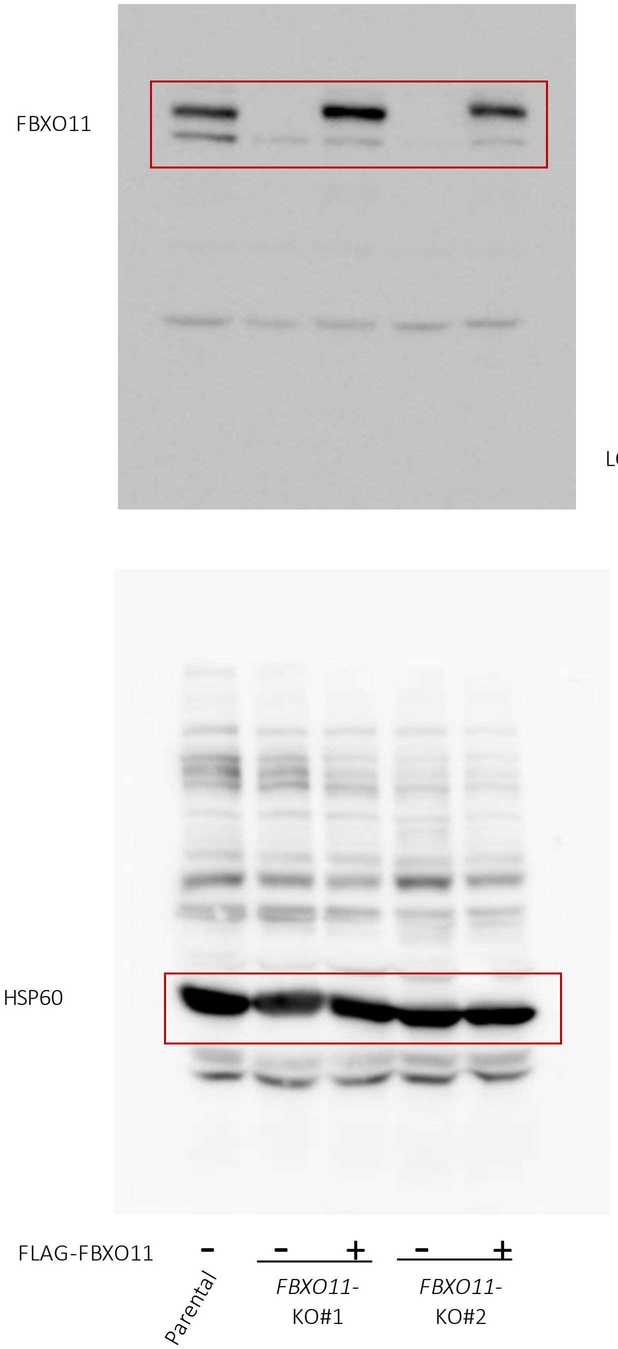

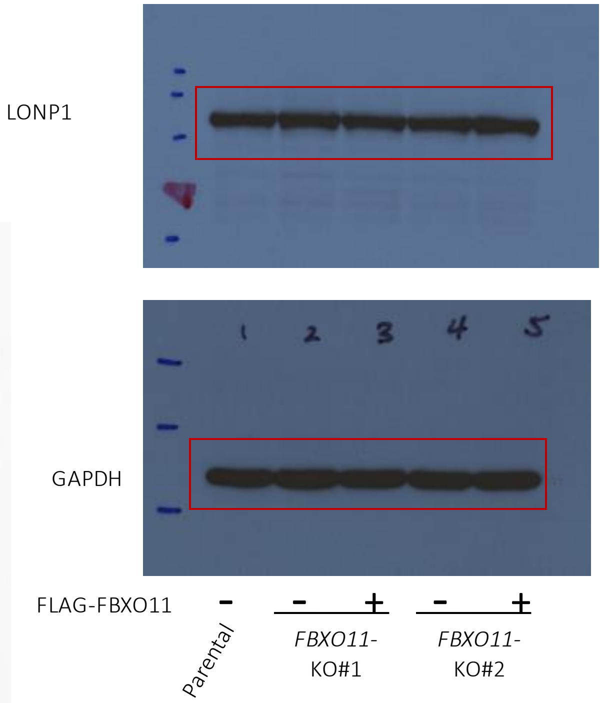

Full unedited blots – Figure 4h

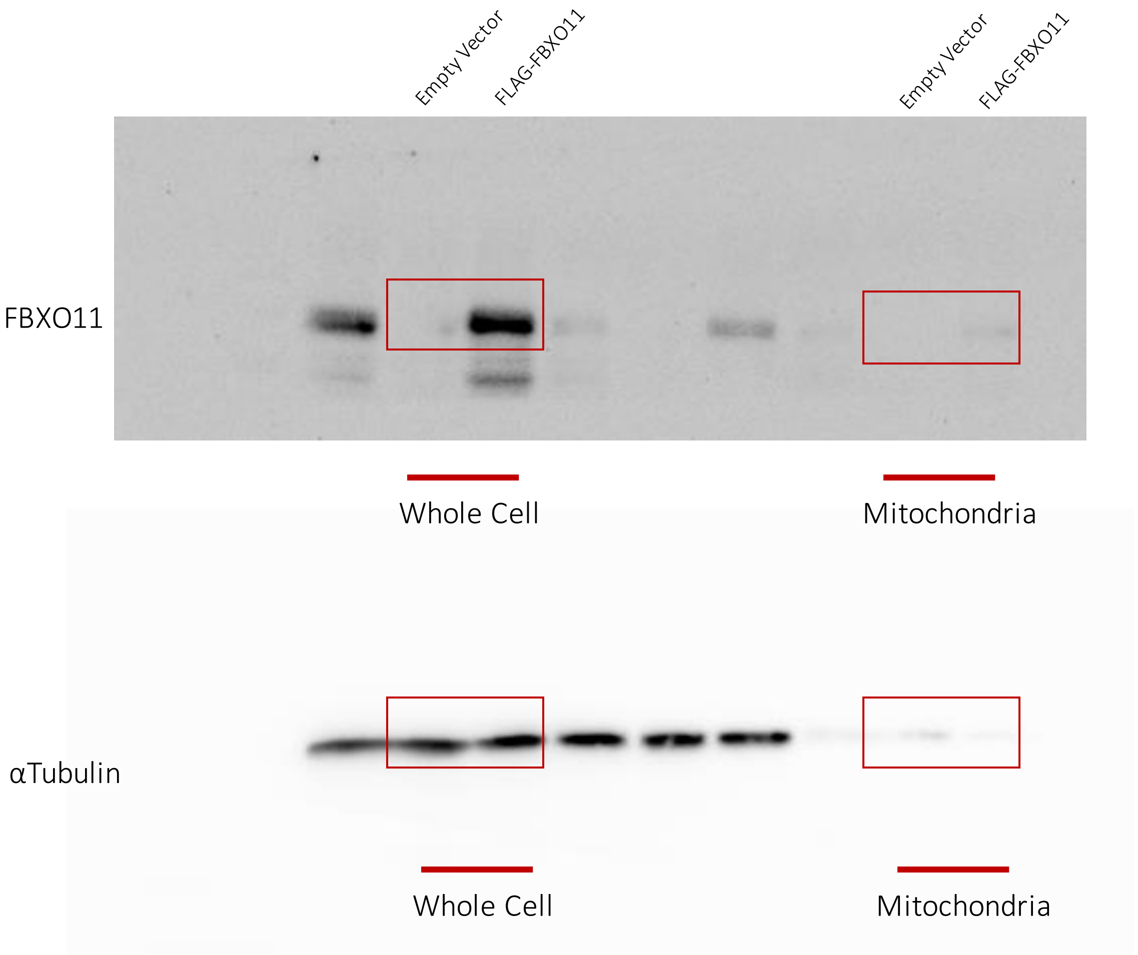

Full unedited blots – Figure 4h

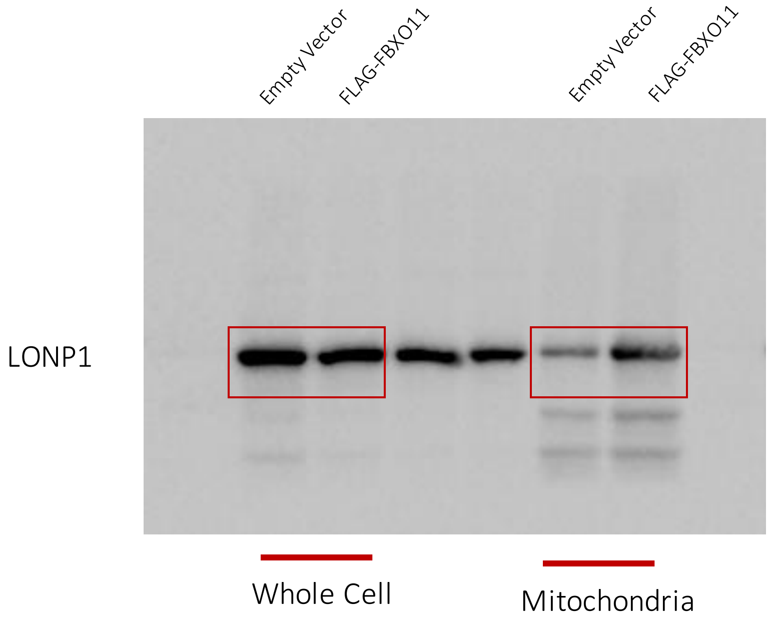

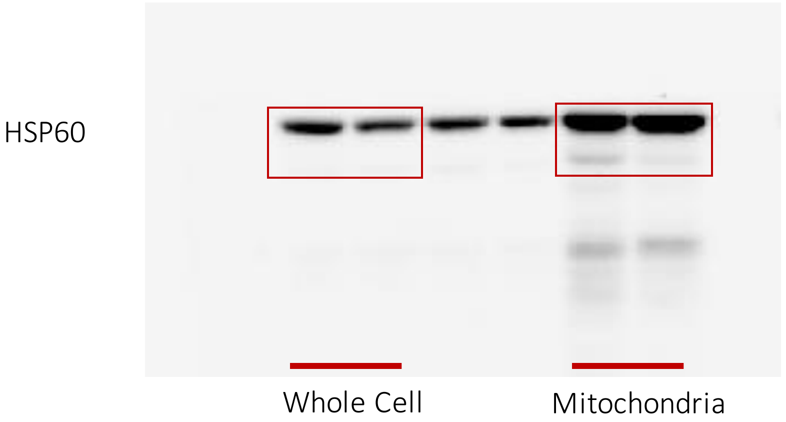

Full unedited blots – Figure 4i

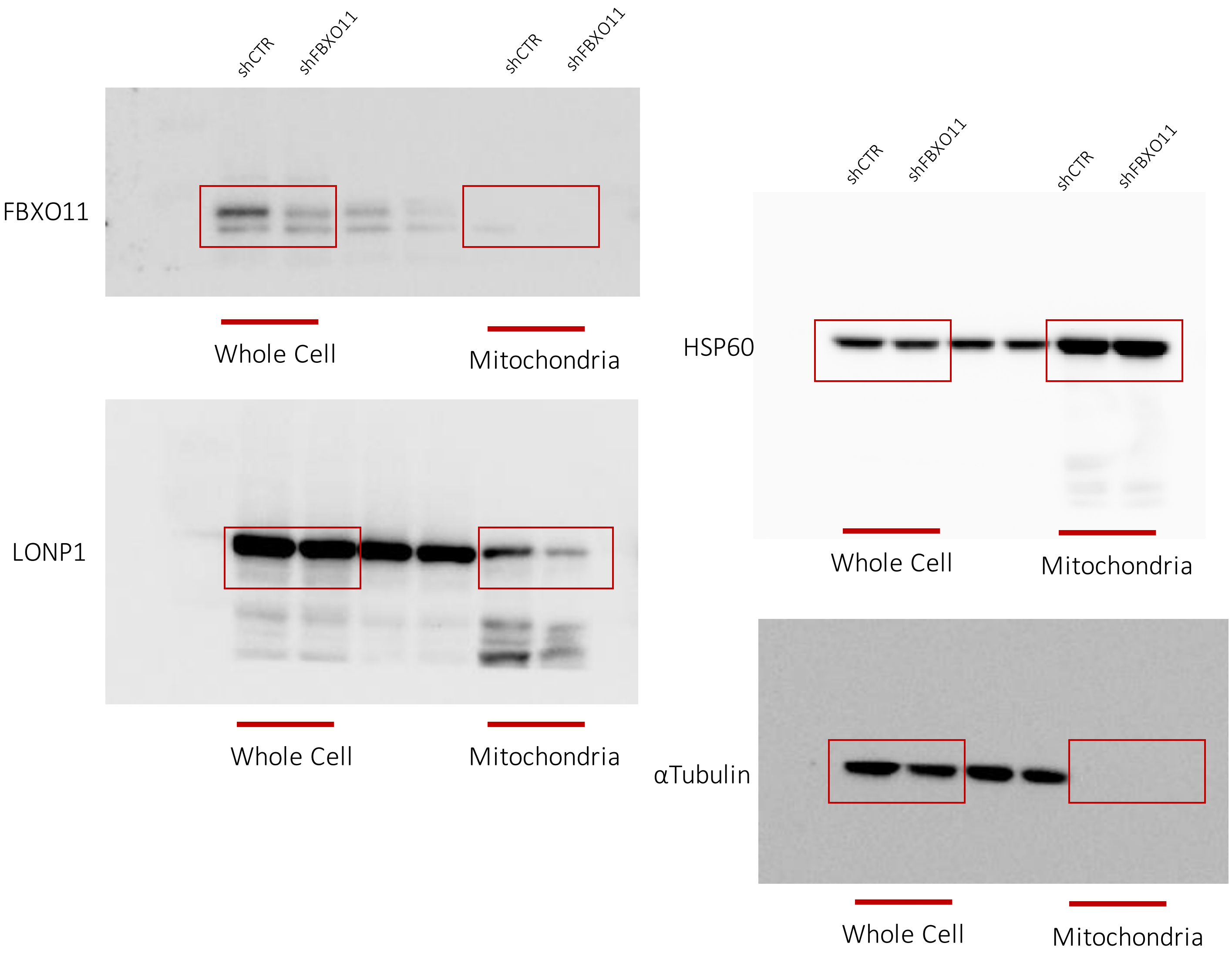

Full unedited blots – Figure 5d (input)

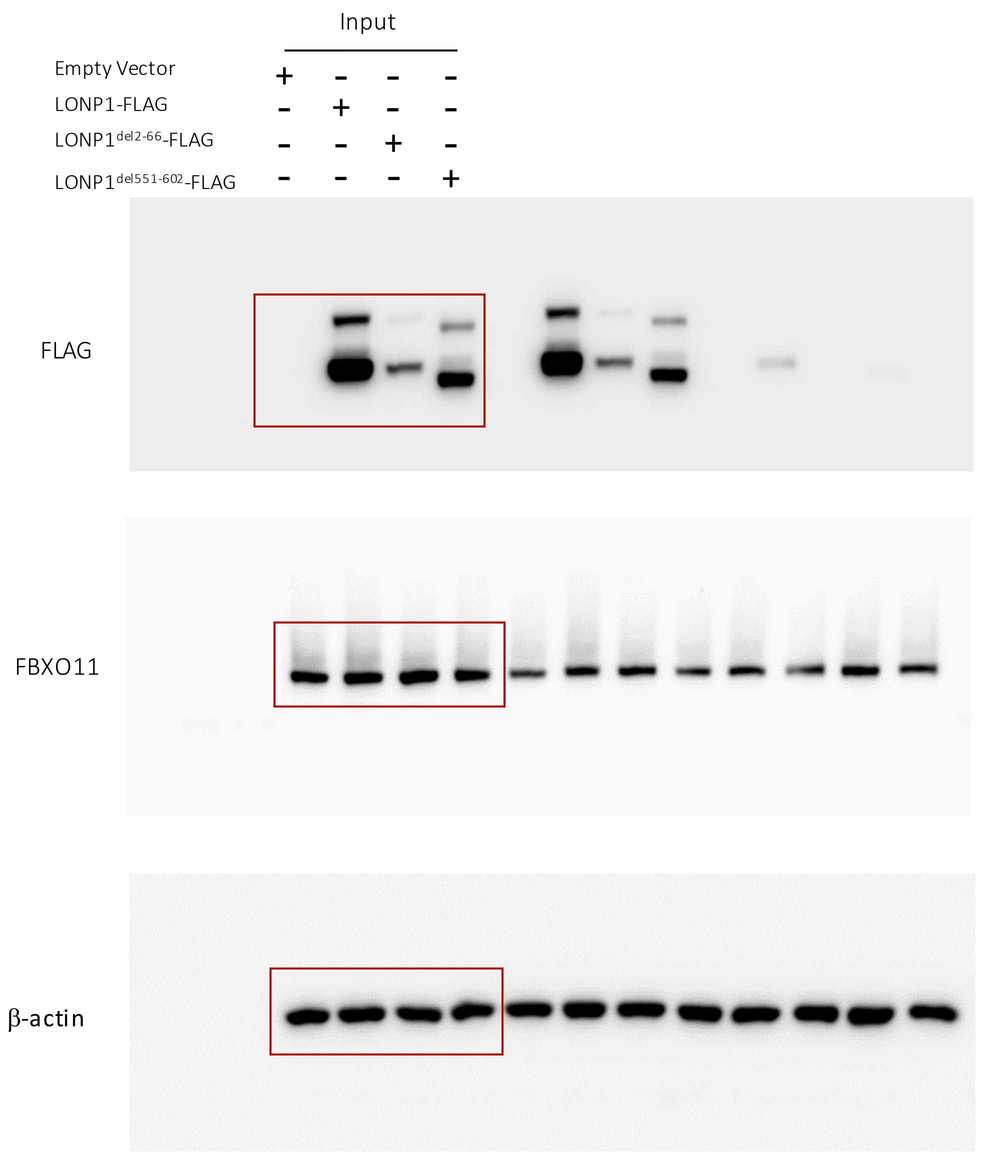

Full unedited blots – Figure 5d (IgG and FLAG IP)

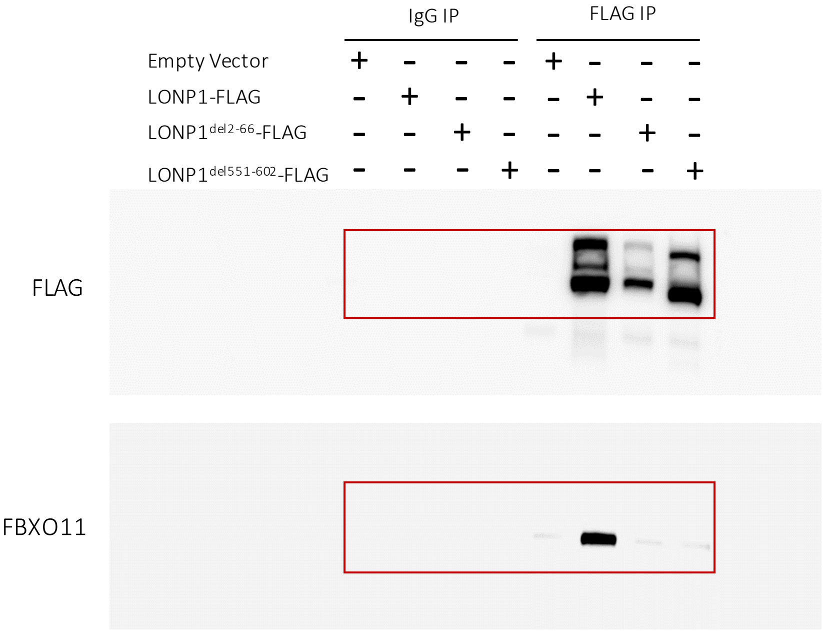

Full unedited blots – Figure 6j

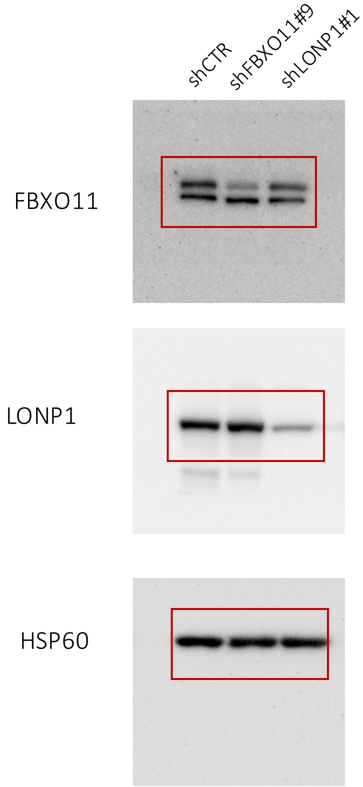

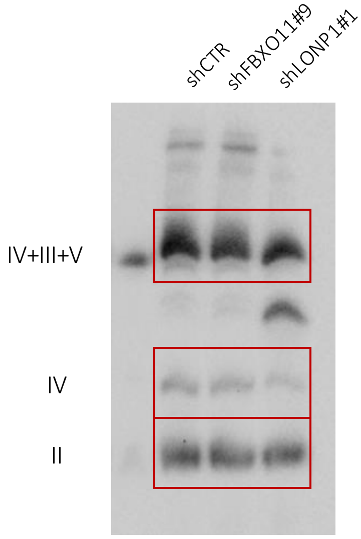

Full unedited blots – Figure 6k

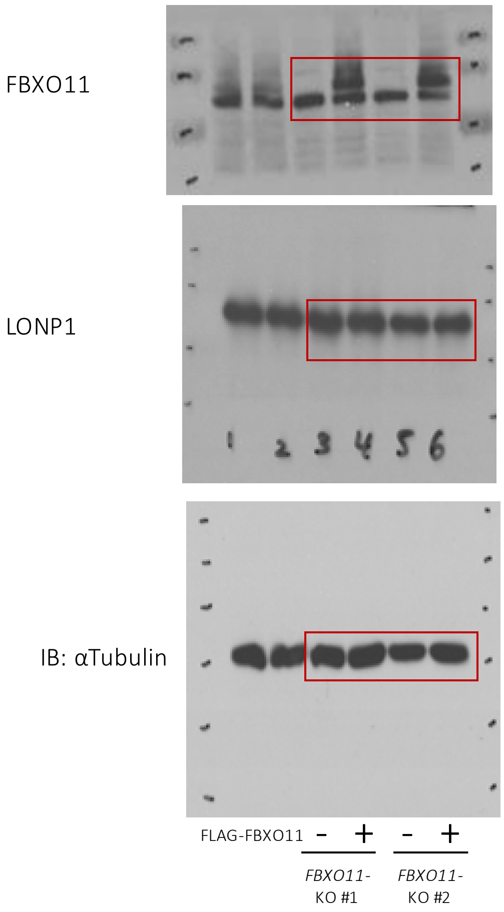

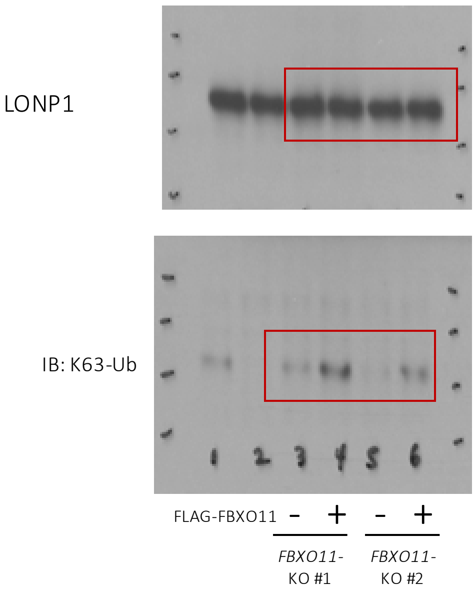

Full unedited blots – Supplemental Figure 2d

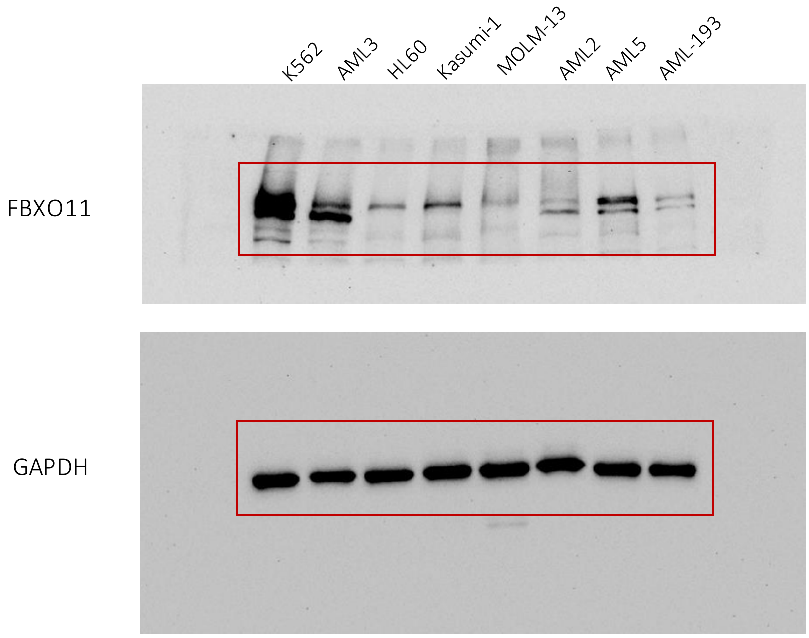

Full unedited blots – Supplemental Figure 2e

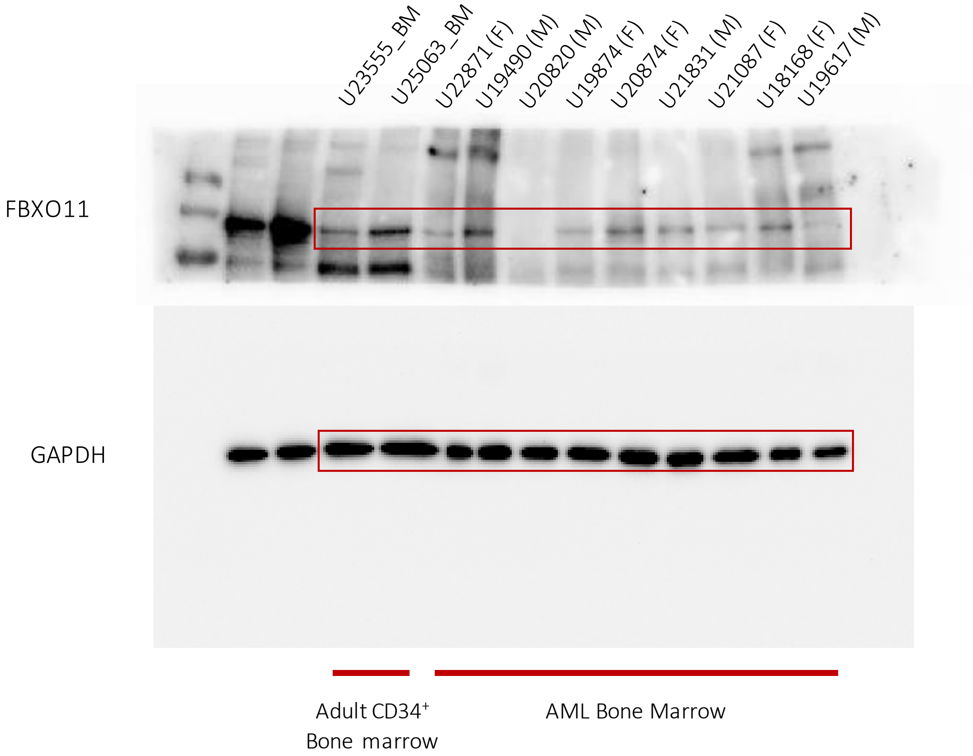

Full unedited blots – Supplemental Figure 3b

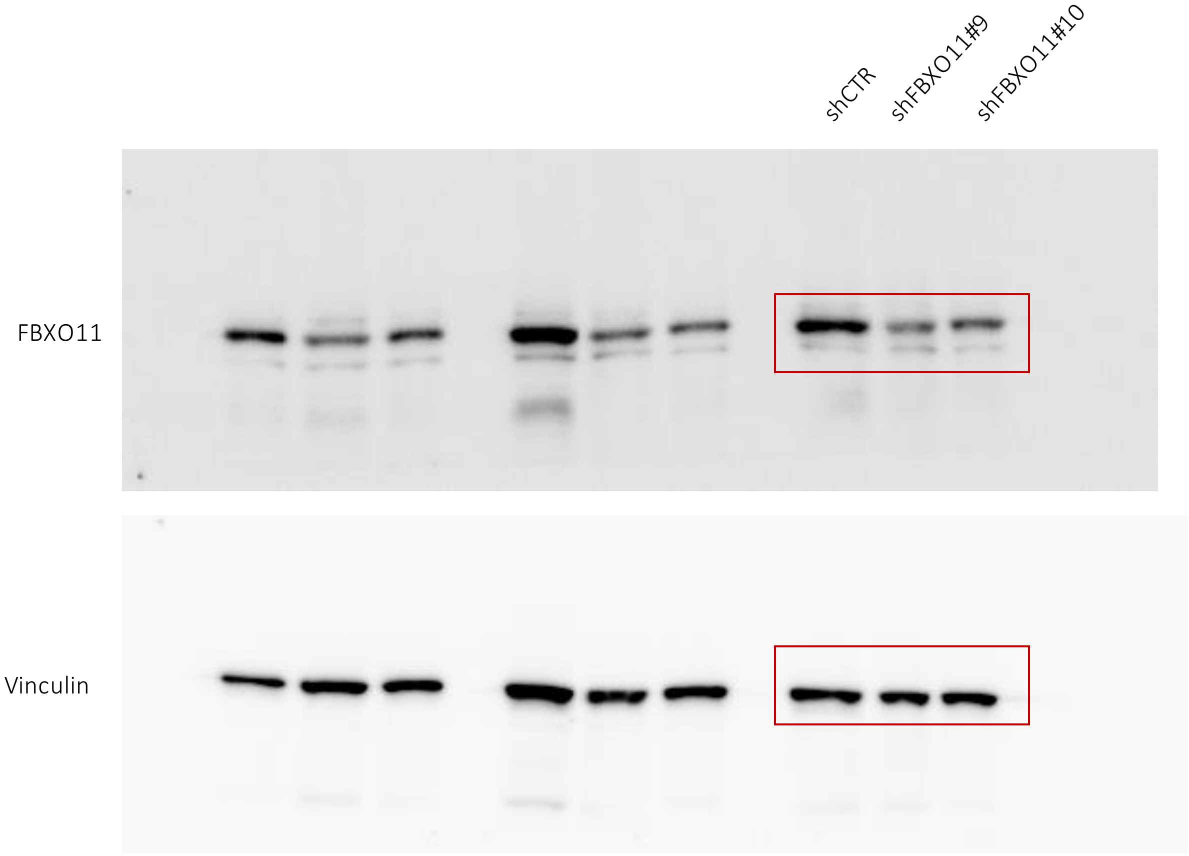

Full unedited blots – Supplemental Figure 5

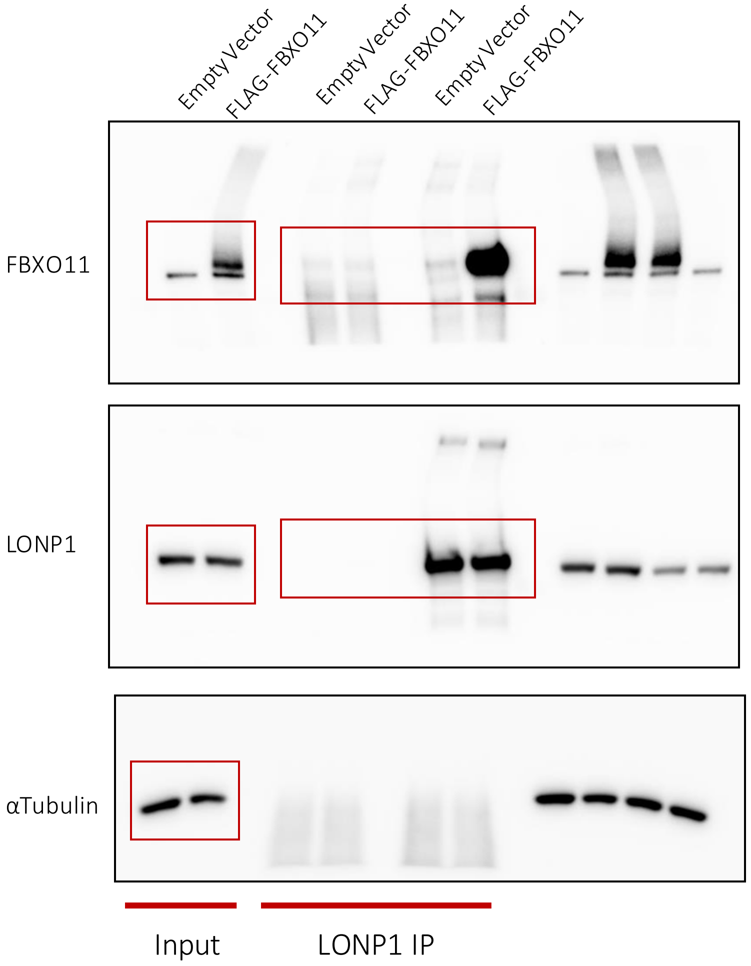

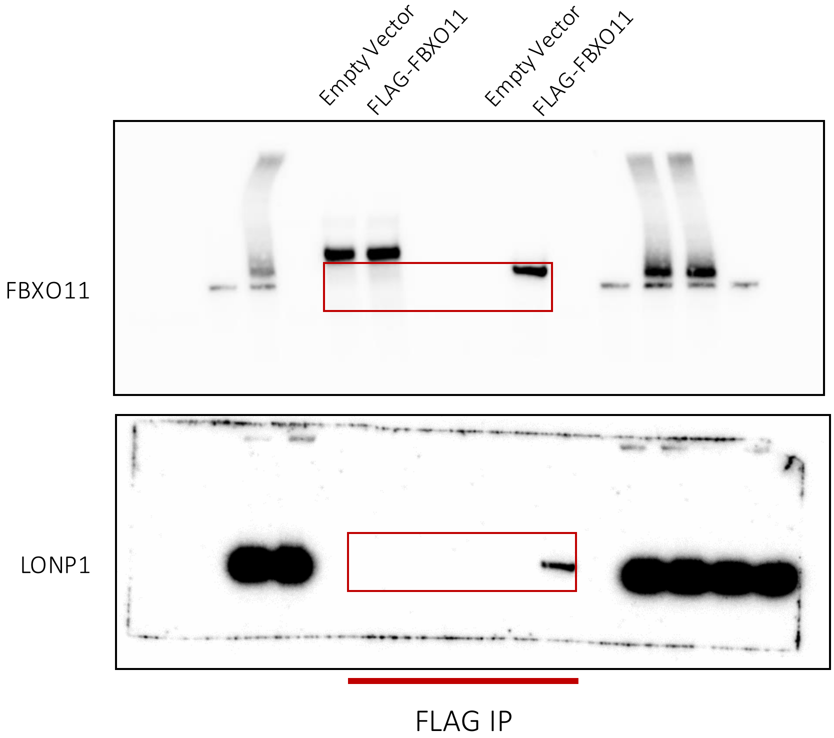

Full unedited blots – Supplemental Figure 7a

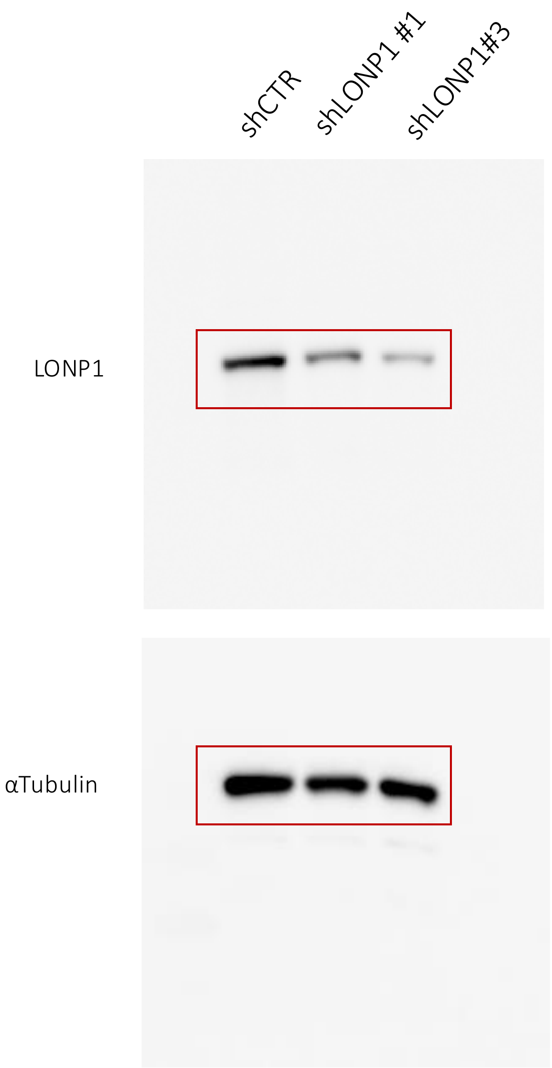

Full unedited blots – Supplemental Figure 7d

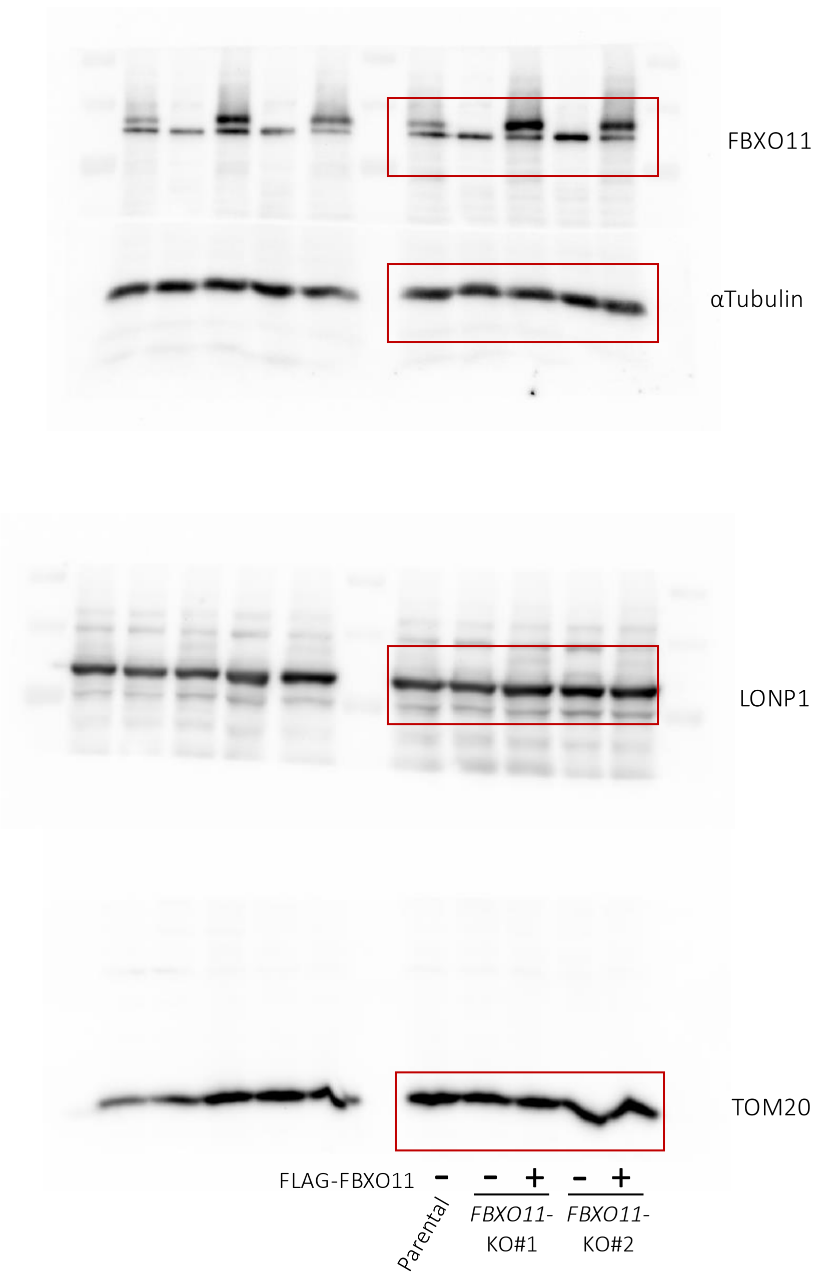

Full unedited blots – Supplemental Figure 8 - Input

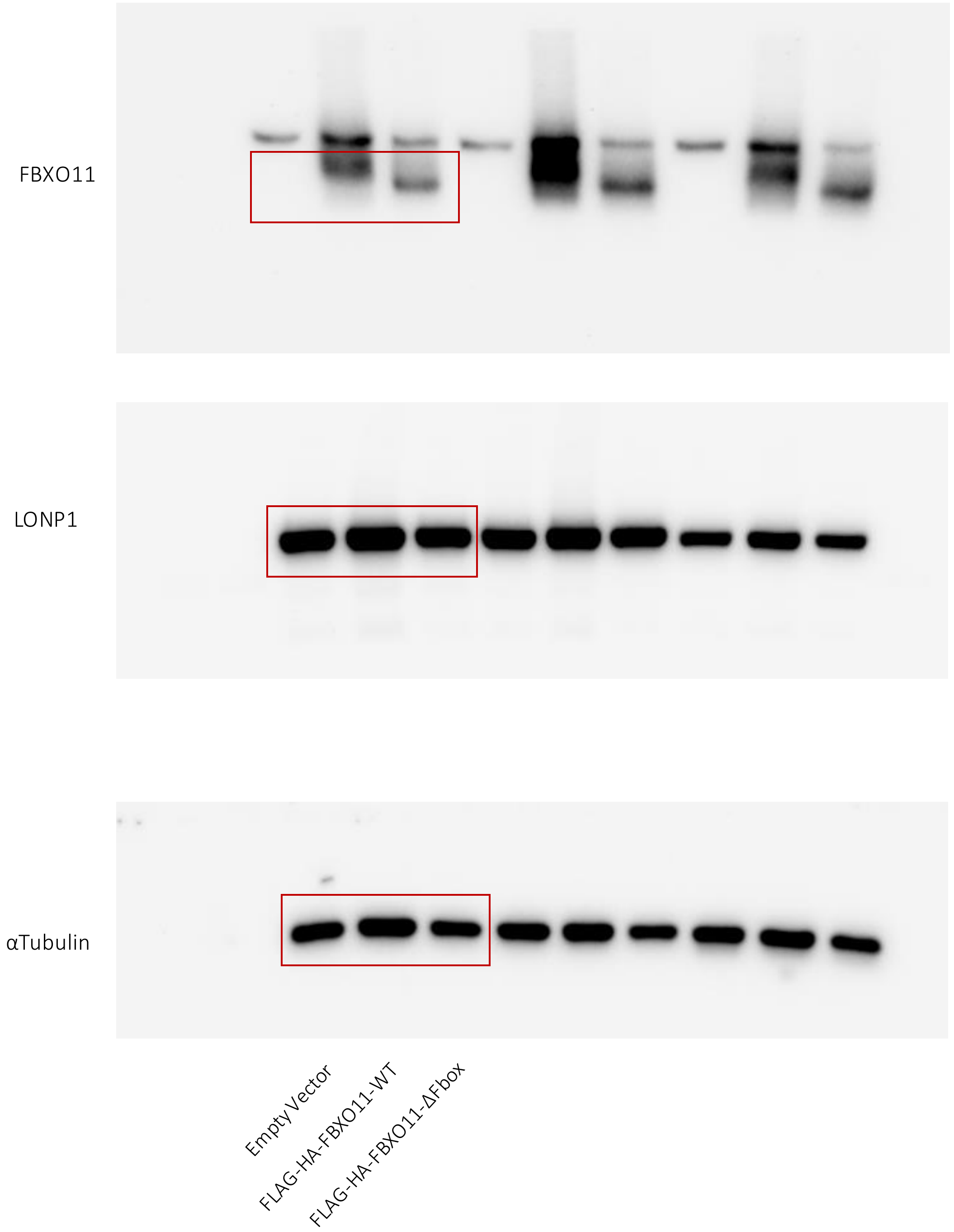

Full unedited blots – Supplemental Figure 8 (LONP1 IP)

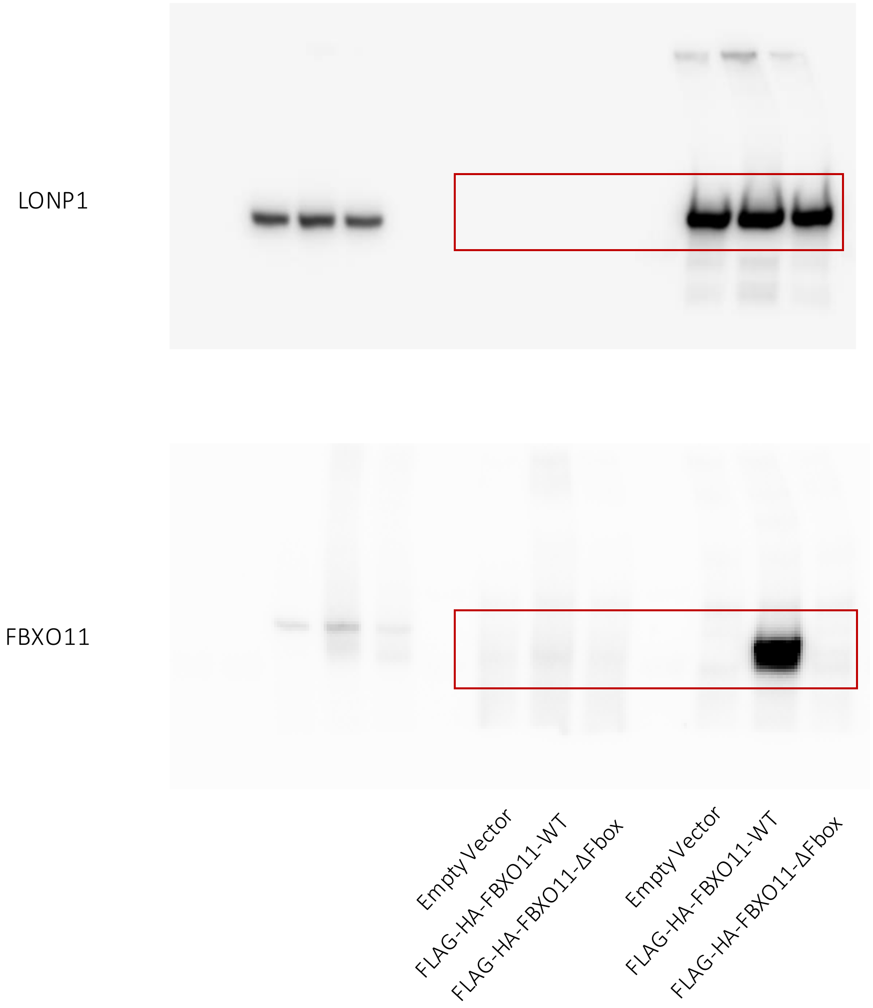

Full unedited blots – Supplemental Figure 8 (FLAG IP)

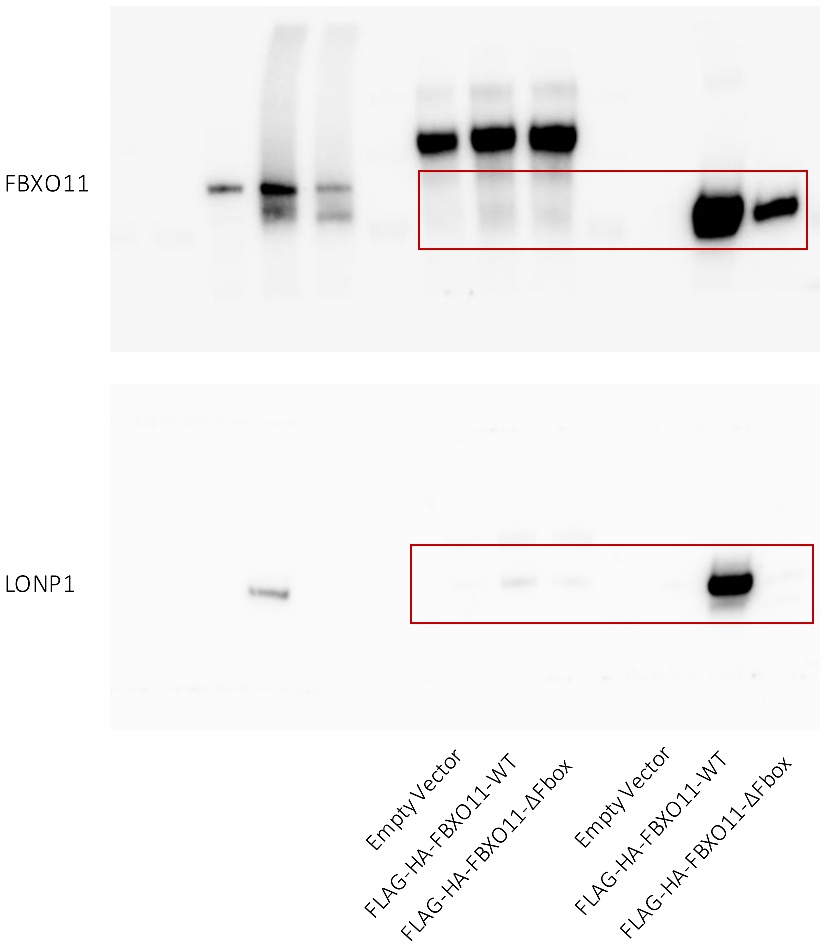

Full unedited blots – Supplemental Figure 9a – Whole cell/Mitochondria

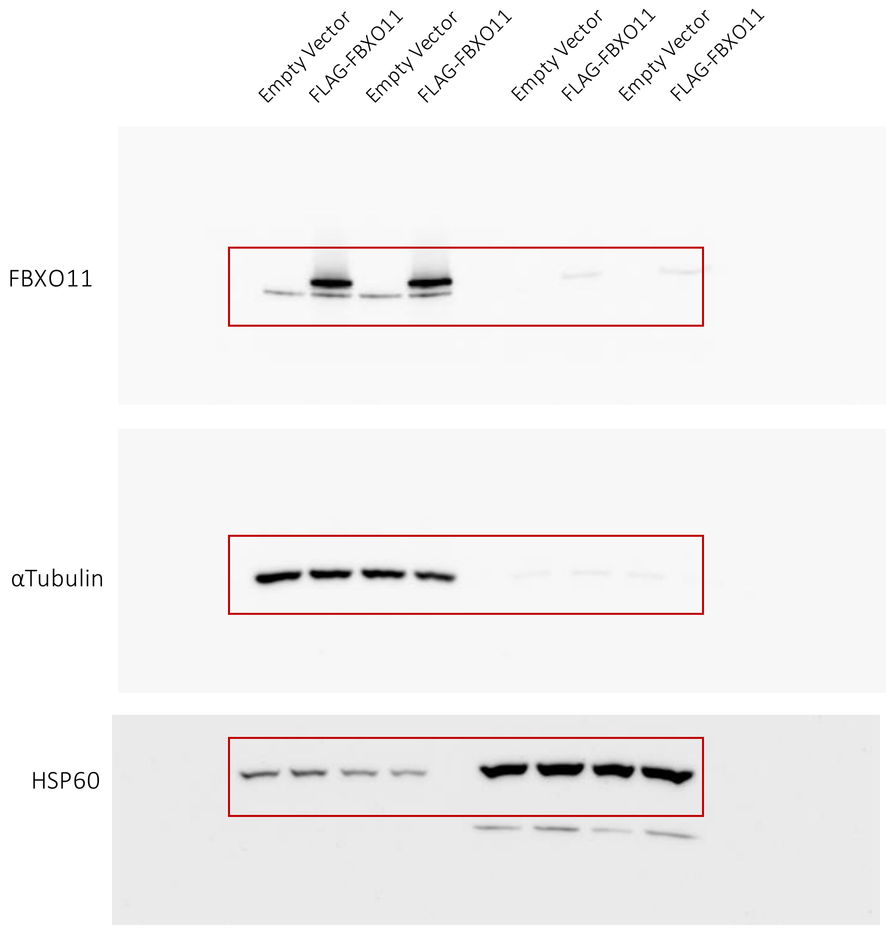

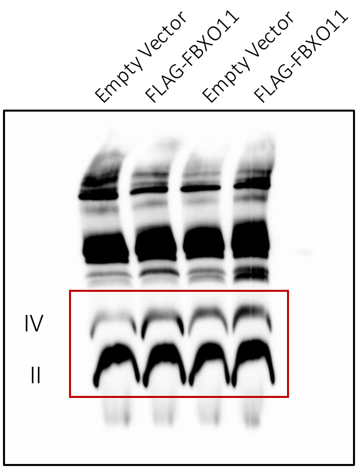

Full unedited blots – Supplemental Figure 9b - Non-Denaturing conditions

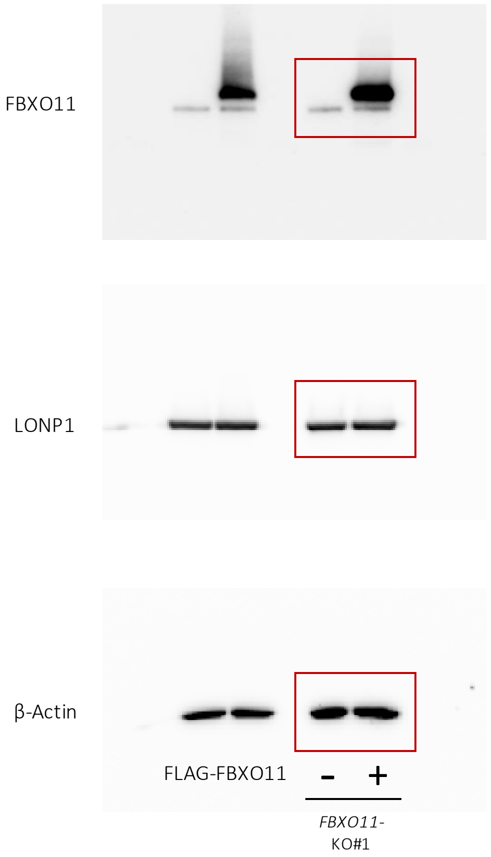

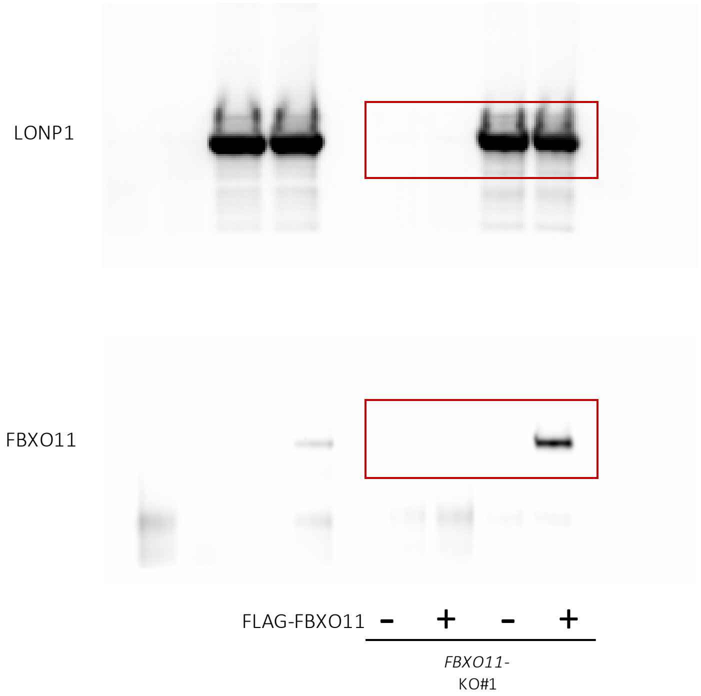

Full unedited blots – Supplemental Figure 9b – Denaturing conditions

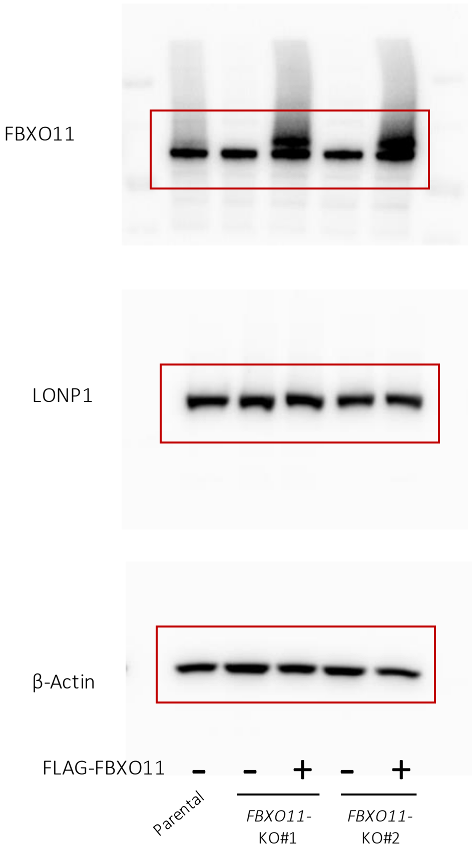

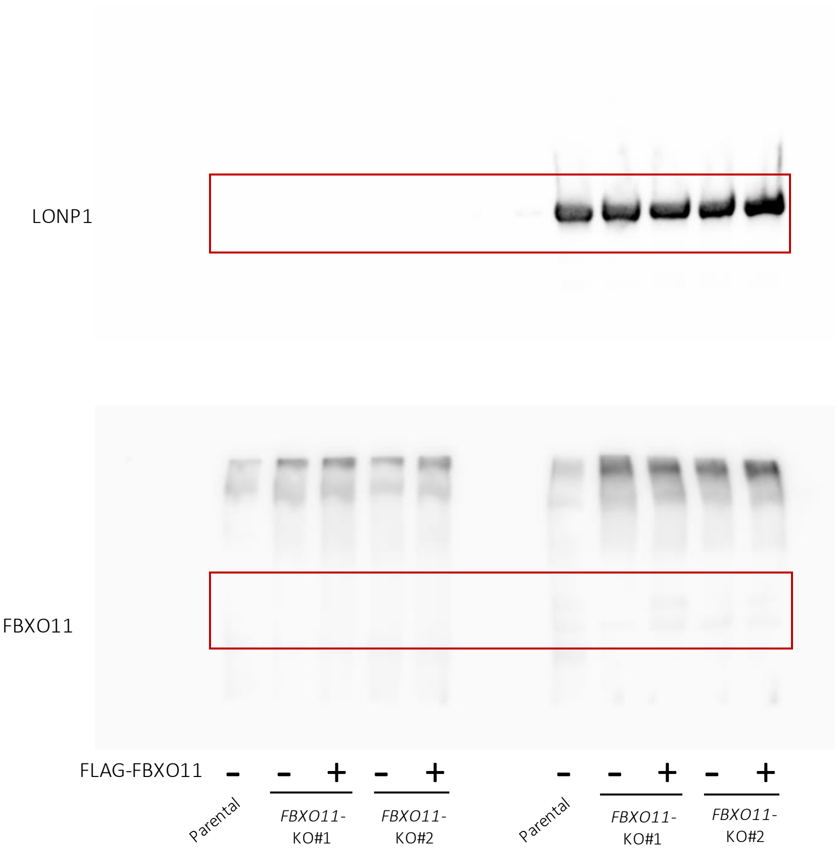

Full unedited blots – Supplemental Figure 9c

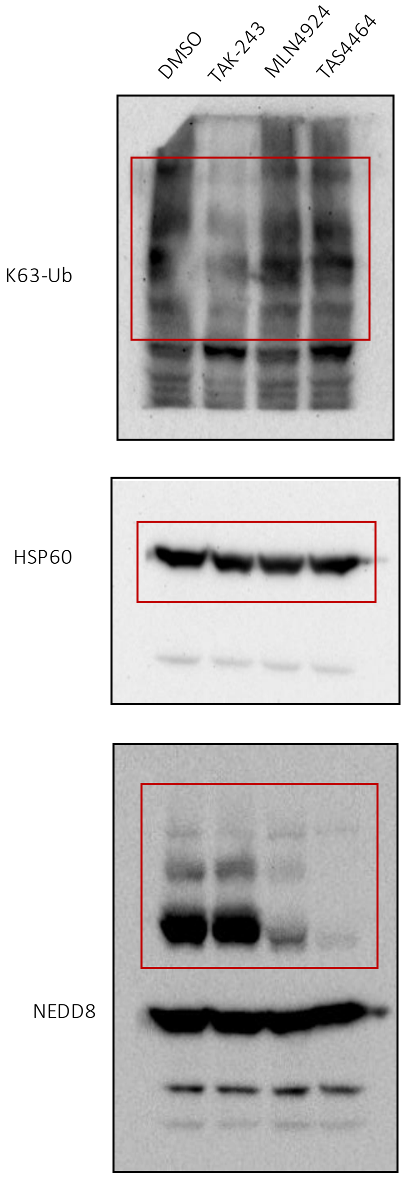

Full unedited blots – Supplemental Figure 12

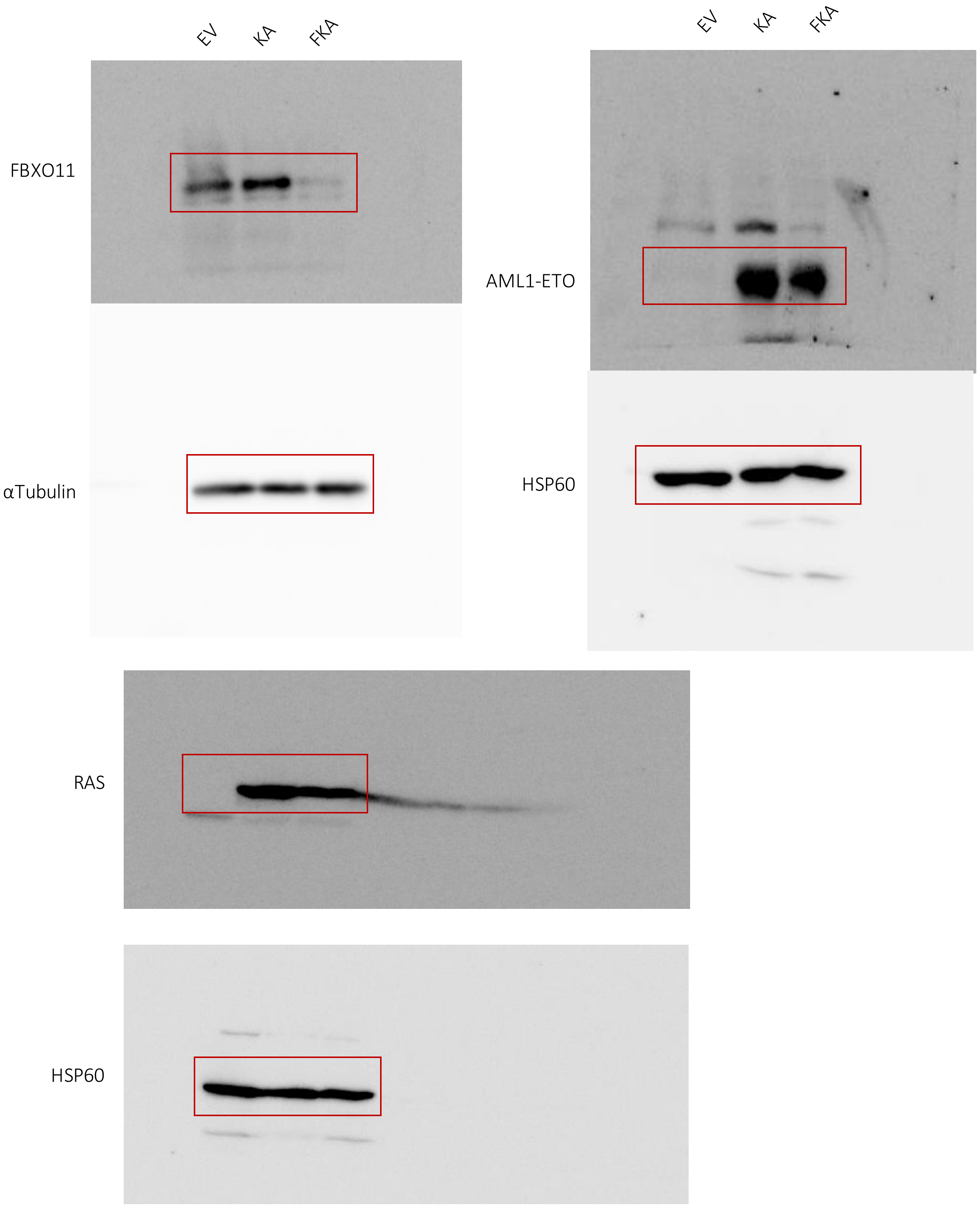

Supplement: Unedited blot and gel images [file jci-136-181943-s194.pdf]
